# Ubiquitination of ACSL4 by Parkin Suppresses Ferroptosis and Rescues Glucocorticoid‐Induced Bone Loss

**DOI:** 10.1002/advs.76586

**Published:** 2026-07-13

**Authors:** Li‐jiang Han, Jian‐sen Miao, Yi‐feng Shi, Yi‐ting Tu, Yu‐zhe Lin, Lin‐Jie Chen, Bing‐hao Lin, Hua‐lin Li, Xuan‐qi Zheng, Hong‐qiang Wu, Hai‐xiao Liu, Lei Yang, Ai‐min Wu, Zhen Lin, Jian Xiao, Gang Zheng, Chen Jin

**Affiliations:** ^1^ Key Laboratory of Orthopaedics of Zhejiang Province Department of Orthopedics The Second Affiliated Hospital and Yuying Children's Hospital of Wenzhou Medical University Wenzhou Zhejiang China; ^2^ Key Laboratory of Osteoporosis and Geriatric Bone Health of Wenzhou Wenzhou Zhejiang China; ^3^ Department of Orthopedics The First Affiliated Hospital of Ningbo University Ningbo Zhejiang China; ^4^ Department of Plastic and Aesthetic Surgery Nanfang Hospital Southern Medical University Guangzhou Guangdong China

**Keywords:** BMSCs, bone‐targeted mRNA delivery, ferroptosis, glucocorticoids‐induced osteoporosis, Parkin/ACSL4

## Abstract

Long‐term glucocorticoids (GCs) use induces glucocorticoids‐induced osteoporosis (GIOP), but the underlying mechanisms remain unclear. This study investigated the role of Parkin in regulating ACSL4‐mediated ferroptosis in GIOP and developed a bone‐targeted mRNA therapeutic strategy. Using dexamethasone (DEX)‐treated bone marrow mesenchymal stem cells (BMSCs) and a GIOP mouse model, we found that GCs induced typical ferroptosis in BMSCs. Parkin expression was significantly downregulated in GIOP models. Parkin knockdown exacerbated DEX‐induced lipid peroxidation, iron accumulation, and mitochondrial dysfunction, while inhibiting osteogenesis and promoting adipogenesis; Parkin overexpression reversed these phenotypes. Mechanistically, Parkin directly bound to ACSL4 and promoted its K48‐linked polyubiquitination and proteasomal degradation. Based on these findings, we constructed DSS6‐modified bone‐targeted lipid nanoparticles (LNPs) (Parkin‐LNP@DSS6), which efficiently encapsulated mRNA and exhibited excellent bone‐homing ability. Parkin‐LNP@DSS6 inhibited ferroptosis and restored osteogenic differentiation in vitro, and significantly improved bone microstructural parameters in GIOP mice. Transcriptome sequencing confirmed suppression of ferroptosis and fatty acid metabolism pathways. Collectively, this study reveals a novel mechanism by which Parkin inhibits BMSCs ferroptosis via K48 ubiquitination‐mediated degradation of ACSL4 to alleviate GIOP, and provides a promising bone‐targeted mRNA delivery strategy for GIOP and other ferroptosis‐related bone diseases.

## Graphical abstract

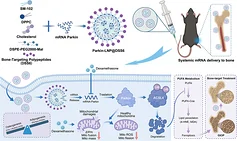

## 1 Introduction

Glucocorticoids (GCs) are widely used as anti‐inflammatory and immunosuppressive agents in clinical practice for the treatment of various autoimmune diseases, inflammatory disorders, and organ transplant rejection [1]. However, long‐term or high‐dose GCs use leads to serious skeletal complications, the most typical of which is glucocorticoids‐induced osteoporosis (GIOP) [2]. GIOP is one of the most common forms of secondary osteoporosis, characterized by decreased bone formation and relatively increased bone resorption, resulting in bone microstructural damage, increased bone fragility, and a significantly elevated risk of fractures [3]. Although bisphosphonates, teriparatide, and other drugs are currently available for the management of GIOP, their efficacy is limited, and long‐term use is associated with adverse effects. Therefore, elucidating the pathogenesis of GIOP and developing novel therapeutic strategies are of great clinical significance.

Bone marrow mesenchymal stem cells (BMSCs) are an important source of osteoblasts, and their functional status directly determines the efficiency of bone formation [4]. Studies have demonstrated that GCs induce excessive apoptosis, autophagic dysregulation, and impaired osteogenic differentiation of BMSCs, concomitantly promoting adipogenic differentiation, which shifts the differentiation balance toward adipogenesis and aggravates bone loss [5, 6]. In recent years, ferroptosis, a newly identified form of regulated cell death, has gradually attracted attention in the field of bone metabolism. Ferroptosis is an iron‐dependent form of cell death characterized by excessive accumulation of lipid peroxides, and it is morphologically and mechanistically distinct from classical apoptosis, necrosis, and autophagy [7, 8]. Excessively reactive ferrous iron accelerates the generation of reactive oxygen species (ROS) via the Fenton reaction, and these ROS interact with polyunsaturated fatty acids (PUFAs) in lipid membranes to induce lipid peroxidation, thereby disrupting cell membrane integrity and fluidity [9]. Accumulating evidence has implicated ferroptosis in the pathogenesis of skeletal disorders such as osteoporosis and osteoarthritis [10, 11]. However, the precise mechanism by which GCs induce ferroptosis in BMSCs and thereby promote the development and progression of GIOP remains unclear.

Parkin (PARK2) is an E3 ubiquitin ligase originally identified as being associated with the pathogenesis of Parkinson's disease. Recent studies have revealed that Parkin exerts important protective functions in cardiovascular diseases, neurodegenerative diseases, metabolic diseases, and tumors by ubiquitinating substrate proteins, regulating mitophagy, and maintaining mitochondrial function [12, 13]. Previous studies have demonstrated that Parkin inhibits iron overload‐induced cardiomyocyte ferroptosis by ubiquitinating long‐chain acyl‐CoA synthetase 4 (ACSL4) [14]. ACSL4 is a key regulator of the ferroptosis pathway; it catalyzes the conversion of PUFAs into fatty acyl‐CoAs and promotes their incorporation into membrane phospholipids, particularly phosphatidylethanolamine (PE), thereby providing substrates for lipid peroxidation [15]. Nevertheless, whether the Parkin‐ACSL4 signaling axis regulates ferroptosis in BMSCs and whether it participates in the pathological process of GIOP have not yet been reported.

Based on the above background, we hypothesized that GCs downregulate Parkin expression, leading to ACSL4 accumulation and aberrant PUFA‐phospholipid metabolism, which in turn induces ferroptosis in BMSCs and ultimately promotes the development of GIOP. Moreover, restoring Parkin levels via bone‐targeted delivery of Parkin mRNA could inhibit ferroptosis and alleviate GIOP. To test this hypothesis, we first confirmed that GCs induce ferroptosis in BMSCs both in vitro and in vivo. We further found that Parkin expression is downregulated in GIOP models, and that Parkin overexpression inhibits ferroptosis by regulating mitochondrial morphology and dynamics, remodeling PUFA‐phospholipid metabolism, binding to ACSL4, and promoting its K48‐linked ubiquitination and degradation. On this basis, we constructed bone‐targeted lipid nanoparticles (Parkin‐LNPs@DSS6) for delivering Parkin mRNA, validated their protective effect on BMSCs in vitro, and demonstrated that they effectively ameliorate bone loss and bone microstructural damage in a GIOP animal model. Finally, transcriptomic profiling revealed key signaling pathways involved in the therapeutic effect of Parkin on GIOP.

In summary, this study reveals for the first time a central role of the Parkin‐ACSL4 regulatory axis in GIOP‐associated BMSCs ferroptosis and proposes a novel therapeutic strategy based on bone‐targeted LNP‐mediated delivery of Parkin mRNA. These findings not only deepen our understanding of the pathogenesis of GIOP but also provide new insights for the treatment of other skeletal diseases associated with ferroptosis.

## 2 Materials and Methods

### 2.1 Ethical Approval

The Animal Ethics Committee of Wenzhou Medical University approved all animal procedures (No. wydw2026‐0299). The experiments were conducted in compliance with the NIH guidelines, and all possible measures were taken to reduce animal suffering.

### 2.2 Reagents and Antibodies

Dexamethasone (DEX) (HY‐14648), erastin (ferroptosis inducer), ferrostatin‐1 (Fer‐1) (selective ferroptosis inhibitor), and MG132 (proteasome inhibitor) were obtained from MedChemExpress (Shanghai, China). Detailed information of the primary antibodies is provided in Table S1. DCFH‐DA and JC‐1 fluorescent dye were obtained from Beyotime (Shanghai, China). Other reagents and their sources were as follows: C11‐BODIPY and MitoSox (Invitrogen, CA, USA), FerroOrange (Tongren, Beijing, China), phosphate‐buffered saline (PBS), fetal bovine serum (FBS), 0.25% trypsin, and dulbecco's modified eagle's dedium (DMEM) (Gibco, New York, USA). All additional chemicals were obtained from Sigma–Aldrich (St. Louis, MO, USA) and Elabscience (Wuhan, China).

### 2.3 Cell Lines and Cell Culture

BMSCs were isolated as previously described [16]. Mouse cranial osteoblast precursor cell line (MC3T3) cells were purchased from the American Type Culture Collection (ATCC), and HEK293T cells were obtained from Procellsystem. All cells were cultured under standard conditions in DMEM supplemented with 10% FBS and 1% penicillin‐streptomycin at 37°C in a humidified atmosphere containing 5% CO_2_. Cultures were routinely monitored to ensure logarithmic growth and regularly tested for mycoplasma contamination. In subsequent in vitro experiments, cells were treated with 200 µm DEX for 48 h.

### 2.4 Flow Cytometry Analysis

Cell apoptosis and necrosis were assessed using an Annexin V‐FITC/PI double‐staining kit (Beyotime, Shanghai, China) according to the manufacturer's protocol. Briefly, BMSCs were harvested after the indicated treatments, washed twice with cold PBS, and resuspended in 500 µL of binding buffer. The cells were then incubated with 5 µL of Annexin V‐FITC and 5 µL of propidium iodide (PI) for 15 min at room temperature in the dark. Annexin V‐FITC emits green fluorescence (FITC channel, excitation 488 nm, emission 530 nm), and PI emits red fluorescence (PE channel, excitation 488 nm, emission 585 nm). The stained cells were immediately analyzed using a flow cytometer (BD Biosciences, USA). At least 10 000 events were acquired per sample. Annexin V‐FITC single‐positive cells were considered early apoptotic, whereas Annexin V‐FITC/PI double‐positive cells were considered late apoptotic or necrotic. Data were analyzed using FlowJo software (FlowJo software, Oregon, USA).

### 2.5 Measurement of Malondialdehyde (MDA) Content

Lipid peroxidation was evaluated using an MDA assay kit (BC0025, Solarbio, Shanghai, China). Cell or tissue samples were mixed with 1 mL of extraction buffer and sonicated (20 W, alternating 3s on and 10 s off, 30 cycles). The homogenate was centrifuged at 4°C and 8000 rpm for 10 min, and the resulting supernatant was incubated with the MDA detection solution. The mixture was then heated in a boiling water bath for 60 min, followed by centrifugation at 10 000×g for 10 min. An aliquot of 200 µL of the supernatant was transferred to a 96‐well plate, and the absorbance was measured at 450, 532, and 600 nm using a microplate reader.

### 2.6 Reverse Transcription Quantitative Real‐Time Polymerase Chain Reaction (RT‐qPCR)

Total RNA was extracted from treated NP cells using TRIzol reagent (Invitrogen, USA) according to the manufacturer's instructions. The concentration and purity of the extracted RNA were assessed. After quality confirmation, first‐strand cDNA was synthesized by reverse transcription using HiScript III RT SuperMix (Vazyme, China). qPCR reactions were performed using AceQ SYBR Green Master Mix (Vazyme, China) on a CFX96 Touch Real‐Time PCR System (Bio‐Rad, USA). Technical replicates were included for each sample, and GAPDH was used as an internal control to normalize gene expression levels. Primer sequences are provided in Table S2.

### 2.7 Western Blot

Cells were lysed for 30 min using RIPA lysis buffer supplemented with 1% PMSF to extract total protein. The lysate was centrifuged at 12 000 rpm for 15 min, and the supernatant was carefully collected for further analysis. Protein concentrations were determined using a BCA assay kit (Beyotime, China). According to the experimental requirements, 20–50 µg of protein from each sample were separated by 6%–12% SDS‐PAGE, and the resolved proteins were electrotransferred onto PVDF membranes (Millipore, USA). After transfer, the membranes were blocked with 5% skim milk powder solution at room temperature for 1 h to prevent non‐specific binding. Subsequently, the membranes were incubated with the corresponding primary antibodies at 4°C overnight. The next day, the membranes were washed three times with Tris‐buffered saline containing 0.1% Tween‐20 (TBST), 10 min each wash, followed by incubation with HRP‐conjugated secondary antibodies at room temperature for 1 h. After washing, enhanced chemiluminescence substrate (Thermo Fisher, USA) was used for signal development, and the chemiluminescent signals were captured using a ChemiDoc MP Imaging System (Bio‐Rad, USA).

### 2.8 Iron Content Analysis

Total iron content in cells was determined using an iron assay kit (Elabscience, Wuhan, China). Cell samples were prepared by mixing 10^6^ cells with 500 µL of lysis buffer, followed by homogenization and complete dissolution at 4°C. The mixture was then centrifuged at 10 000×g for 10 min, and the supernatant was collected. Tissue total iron content was measured using another iron assay kit (Elabscience, Wuhan, China). For animal samples, 0.2 g of fresh mouse heart tissue was homogenized in 500 µL of lysis buffer at a low temperature. After centrifugation at 10 000×g for 10 min at 4°C, the supernatant was discarded. Subsequently, 300 µL of standard solutions at various concentrations were added to the corresponding 1.5 mL EP tubes. For the test samples, 300 µL of the sample was added to the respective EP tubes. Then, 150 µL of chromogenic solution was added to each tube, and the mixture was incubated at 37°C for 40 min, followed by centrifugation at 12 000×g for 10 min. Finally, 300 µL of the supernatant from each tube was transferred to the corresponding wells of a microplate, and the absorbance at 593 nm was measured using a microplate reader.

### 2.9 Cell Transfection

For transient overexpression, BMSCs were seeded in 6‐well plates at a density of 2 × 10^5^ cells per well and cultured overnight to reach 70%–80% confluence. Plasmids encoding Parkin (OE‐Parkin), ACSL4 (OE‐ACSL4), or the empty vector control (OE‐NC) were transfected using Lipofectamine 3000 (Invitrogen, USA) according to the manufacturer's instructions. Briefly, 2 µg of plasmid DNA was diluted in 125 µL of Opti‐MEM (Gibco, USA) and mixed with 125 µL of Opti‐MEM containing 4 µL of Lipofectamine 3000 and 5 µL of P3000 reagent. The mixture was incubated for 15 min at room temperature and then added dropwise to the cells. After 6 h, the medium was replaced with fresh complete culture medium. Cells were harvested for further experiments 48 h after transfection. For stable Parkin knockdown, lentiviral vectors expressing short hairpin RNA (shRNA) targeting Parkin (sh‐Parkin) or a negative control (sh‐NC) were constructed. BMSCs were infected with lentivirus at a multiplicity of infection (MOI) of 50 in the presence of 5 µg/mL polybrene (Sigma–Aldrich, USA). After 24 h, the medium was replaced with fresh complete medium containing 2 µg/mL puromycin (Invivogen, USA) to select stable transductants for 7 days.

### 2.10 Fluorescence Staining for Lipid Peroxidation and Iron

To detect lipid peroxidation and intracellular Fe^2^^+^ levels, BMSCs were seeded on glass coverslips in 24‐well plates and subjected to the indicated treatments. For C11‐BODIPY (581/591) staining (Invitrogen, USA), cells were incubated with 1 µmol/L C11‐BODIPY in phenol‐red‐free DMEM for 30 min at 37°C in the dark, washed twice with PBS, and then fixed with 4% paraformaldehyde for 10 min. For FerroOrange staining (DOJINDO, Japan), cells were incubated with 1 µmol/L FerroOrange in phenol‐red‐free DMEM for 30 min at 37°C in the dark, washed twice with PBS. Nuclei were counterstained with DAPI (1 µg/mL) for 5 min. Fluorescence images were captured using a confocal microscope (Leica, Germany). Image analysis was performed using ImageJ software.

### 2.11 Alkaline Phosphatase (ALP) and Alizarin Red S (ARS) Staining

For ALP staining, BMSCs cultured in osteogenic induction medium for 7 days were fixed with 4% PFA and stained with BCIP/NBT kit (Beyotime, Shanghai, China). For ARS staining, cells induced for 14–21 days were fixed with 70% ethanol or 4% PFA, stained with 1%–2% ARS for 10–20 min, and destained with 10% cetylpyridinium chloride for quantification at 562 nm. Images were captured by light microscopy.

### 2.12 Oil Red O Staining for Adipogenic Differentiation

BMSCs were cultured in adipogenic induction medium for 10–14 days. Cells were then fixed with 4% PFA for 30 min, washed with PBS, and stained at room temperature for 20 min with 0.5% Oil Red O solution (Sigma–Aldrich) dissolved in 60% isopropanol. After staining, cells were gently rinsed with deionized water to remove excess dye. Lipid droplets were visualized using a light microscope.

### 2.13 ROS and Mitochondrial ROS Detection

Intracellular and mitochondrial ROS levels were measured using DCFH‐DA (Beyotime, China) and MitoSOX Red (Invitrogen, USA), respectively. Briefly, treated BMSCs were incubated with 10 µmol/L DCFH‐DA in serum‐free DMEM for 30 min at 37°C in the dark, or with 5 µmol/L MitoSOX Red in HBSS under the same conditions. After two washes with PBS, fluorescence signals were visualized using a confocal microscope (Leica, Germany) at excitation/emission wavelengths of 488/525 nm (DCFH‐DA) and 510/580 nm (MitoSOX). The mean fluorescence intensity was quantified using ImageJ software.

### 2.14 Transmission Electron Microscopy (TEM)

TEM was used to examine cellular ultrastructure. Cells were fixed overnight at 4°C, followed by post‐fixation in 1% osmium tetroxide for 30 min. The samples were then dehydrated through a graded ethanol series and embedded in resin. Ultrathin sections were stained with uranyl acetate and lead citrate, and observed under a Hitachi field‐emission transmission electron microscope.

### 2.15 Surface Plasmon Resonance (SPR) Analysis

SPR analysis was performed using a Biacore T200 instrument (Biacore, Uppsala, Sweden) with recombinant Parkin and ACSL4 proteins. The amino groups of the proteins were coupled to the activated carboxyl groups on a CM5 sensor chip. Briefly, ACSL4 was diluted to 20 µg/mL in 200 µL of 10 mmol/L sodium acetate and injected over the activated chip surface for immobilization. Residual activated sites were blocked with 1 mol/L ethanolamine. To analyze the interaction, various concentrations of Parkin protein (0.625, 1.25, 2.5, 5, 10, 20, and 40 µg/mL) in 20 mmol/L Tris‐HCl buffer were flowed over the ACSL4‐immobilized chip.

### 2.16 Immunoprecipitation (IP)

Cells were lysed in 500 µL of NP‐40 lysis buffer at 4°C for 30 min. After centrifugation, a 50‐µL aliquot of the lysate was saved as the input sample, and the remaining supernatant was used for IP. The samples were incubated with 2 µg of anti‐GFP or anti‐Parkin antibody and 50 µL of Protein‐A/G PLUS‐Agarose (Santa Cruz Biotechnology Co., Ltd., Shanghai, China) overnight at 4°C with gentle rotation. The beads were then washed twice with 1 mL of NP‐40 lysis buffer, mixed with 25 µL of sample buffer, and boiled for 10 min. The released proteins were separated by 12% SDS‐PAGE and detected by immunoblotting using an anti‐ACSL4 antibody as described above.

### 2.17 Single‐Cell RNA Sequencing Analysis

The scRNA‐seq dataset GSE261072 was analyzed using Seurat. Cells with low or high gene counts or high mitochondrial content were filtered. After log‐normalization, UMAP was used for visualization. DEGs between the control and GCs groups were identified (|log_2_FC| ≥ 0.5, adjusted *p* < 0.05). A ferroptosis module score was calculated using the AddModuleScore function. Feature plots were generated for ferroptosis‐associated genes.

### 2.18 Molecular Docking

Molecular docking was performed using HADDOCK, an information‐driven flexible docking method for modeling biomolecular complexes, as described previously [17]. HADDOCK integrates various experimental and/or bioinformatics data to guide the modeling process, allowing more sophisticated handling of conformational flexibility and focusing the search on relevant regions of the interaction space. PyMOL was used for result analysis and visualization.

### 2.19 Preparation of Bone‐Targeted Parkin‐LNP@DSS6

Parkin mRNA was purchased from CYNBIO (Shanghai, China). LNPs were formulated using the following lipid components: SM‐102, DPPC (1,2‐dipalmitoyl‐sn‐glycero‐3‐phosphocholine), cholesterol, and DSPE‐PEG2000‐Mal (1,2‐distearoyl‐sn‐glycero‐3‐phosphoethanolamine‐N‐[maleimide(polyethylene glycol)‐2000]), at a molar ratio of 50:10:38.5:1.5 (SM‐102: DPPC: cholesterol: DSPE‐PEG2000‐Mal). The bone‐targeting polypeptide DSS6 was conjugated to the surface of LNPs via thiol‐maleimide chemistry [18]. Briefly, DSS6 was dissolved in PBS and added to the pre‐formed LNPs at a molar ratio of 10:1 (DSS6 to DSPE‐PEG2000‐Mal), followed by incubation at room temperature for 2 h with gentle shaking. For Parkin‐LNP@DSS6 preparation, 25 µL of Parkin mRNA (1000 ng/µL) dissolved in 50 µL of 20 mm Tris‐HCl buffer was rapidly mixed with the lipid mixture using a microfluidic device at an aqueous‐to‐organic volume ratio of 3:1. One minute after mixing, the resulting suspension was incubated at room temperature for 15 min. Subsequently, 1.5 volumes of 1× PBS were added to reduce the ethanol concentration. The LNPs were purified using a desalting column (Thermo Fisher Scientific) equilibrated with PBS. The purified LNPs were filtered through a 0.22‐µm filter and stored at 4°C for further use.

### 2.20 TEM for LNP Morphology

The morphology of LNPs (Vector‐LNP and Parkin‐LNP@DSS6) was examined using transmission electron microscopy. Briefly, a drop of the freshly prepared LNP suspension (diluted to an appropriate concentration with distilled water) was placed onto a carbon‐coated copper grid and allowed to adsorb for 2–3 min. The excess liquid was gently removed with filter paper. The grid was then negatively stained with 2% uranyl acetate solution for 30 s to 1 min, followed by air‐drying at room temperature. Samples were observed using a transmission electron microscope (Hitachi, Japan) operating at an accelerating voltage of 80–120 kV. Representative images were captured at appropriate magnifications to visualize particle shape, size, and surface morphology.

### 2.21 Transcriptomic Analysis

Femoral tissues (distal femur) were collected from control mice and GIOP model mice. Four biological replicates per group (*n* = 4) were used. Total RNA was extracted using TRIzol reagent. Libraries were prepared and sequenced on an Illumina NovaSeq 6000 platform (paired‐end 150 bp). Raw reads were trimmed and aligned to the mouse reference genome (GRCm38) using HISAT2. Differentially expressed genes (DEGs) were identified using DESeq2 with criteria of false discovery rate (FDR) < 0.05 and |log_2_(fold change)| ≥ 1. Gene Ontology (GO) and Kyoto Encyclopedia of Genes and Genomes (KEGG) enrichment analyses were performed using clusterProfiler. Gene set enrichment analysis (GSEA) was conducted with MSigDB hallmark gene sets (FDR < 0.05). Femoral tissues from GIOP mice and GIOP mice treated with Parkin‐LNP@DSS6 (*n* = 4 per group) were processed identically. RNA extraction, library preparation, sequencing, and bioinformatic analyses were performed following the same procedures as described above. DEGs were defined as |log_2_FC| ≥ 1 and FDR < 0.05.

### 2.22 Agarose Gel Electrophoresis for mRNA Encapsulation Efficiency

mRNA encapsulation efficiency was assessed by agarose gel electrophoresis. LNPs containing Parkin mRNA were prepared at various cationic liposome‐to‐mRNA weight ratios (w/w: 1:0, 1:1, 2:1, 4:1, 6:1, 8:1, and 16:1). After complexation, the samples were loaded onto a 1% (w/v) agarose gel pre‐stained with GelRed (Beyotime, China). Electrophoresis was performed in 1× TAE buffer at 100 V for 30 min. Free mRNA was used as a positive control. The gel was visualized under a UV transilluminator (Bio‐Rad, USA). Complete mRNA encapsulation was defined as the absence of a free mRNA band when the liposome‐to‐mRNA ratio exceeded a certain threshold (8:1, w/w).

### 2.23 Dynamic Light Scattering (DLS) for Particle Size and Zeta Potential

The hydrodynamic diameter (size) and zeta potential of LNPs (Vector‐LNP and Parkin‐LNP@DSS6) were measured using a Zetasizer Nano ZS instrument (Malvern Panalytical, UK). Freshly prepared LNP samples were diluted 1:100 with distilled water and transferred into a disposable cuvette (for size measurement) or a folded capillary cell (for zeta potential measurement). Measurements were performed at 25°C with a scattering angle of 173°. Each sample was measured in triplicate, and the results are presented as mean ± standard deviation (SD).

### 2.24 In Vivo Near‐Infrared Fluorescence Imaging

To evaluate the biodistribution of LNPs, indocyanine green (ICG) was labeled onto Vector‐LNP and Parkin‐LNP@DSS6. ICG‐labeled LNPs (Vector‐LNP^ICG^ and Parkin‐LNP@DSS6^ICG^) were intravenously injected into mice (*n* = 9 per group, 3 mice per time point) at a dose of 1 mg/kg ICG equivalent. At 6, 24, and 48 h post‐injection, mice were anesthetized with isoflurane (1%–2% in oxygen) and subjected to whole‐body imaging using an in vivo near‐infrared fluorescence imaging system (PerkinElmer, USA). ICG fluorescence was excited at 745 nm and detected at 820 nm. For ex vivo imaging, mice were euthanized at the indicated time points, and major organs (hindlimbs, spleen, liver, kidneys, and heart) were collected. Fluorescence intensity was measured using the instrument's software. All images were processed with the same normalization settings.

### 2.25 Micro‐Computerized Tomography (Micro‐CT)

The distal femur and femoral head of mice were analyzed using a micro‐CT machine and the accompanying software. Images were acquired at 70 kV, 200 µA, with a spatial resolution of 14.8 µm. Three‐dimensional images were reconstructed from the acquired data. For the trabecular compartment of the distal femur, the volume of interest (VOI) was defined as 100 CT slices starting 2 mm below the growth plate and extending distally. For the femoral head, the VOI was selected as the entire trabecular bone region within the epiphysis, excluding the cortical bone. The built‐in software of the micro‐CT system was used to evaluate the following trabecular bone parameters: bone volume fraction (BV/TV), trabecular thickness (Tb.Th, mm), trabecular number (Tb.N, 1/mm), and trabecular separation (Tb.Sp, mm).

### 2.26 Calcein‐Alizarin Red S Double Labeling

For dynamic bone histomorphometry, animals received intraperitoneal injections of calcein (30 mg/kg) and Alizarin Red S (30 mg/kg) at 28 and 4 days before sacrifice, respectively, according to a standard double‐labeling protocol. After euthanasia, femurs were dissected, fixed in 70% ethanol, dehydrated through a graded ethanol series, and embedded in methyl methacrylate. Coronal sections (5 µm thick) were cut using a microtome and examined without counterstaining under a fluorescence microscope (excitation/emission: 488/520 nm for calcein and 560/580 nm for Alizarin Red S). The following dynamic parameters were measured in the trabecular bone region (0.5–2.0 mm distal to the growth plate) using ImageJ software: mineral apposition rate (MAR, µm/day) = (distance between double labels / time interval between injections); mineralizing surface per bone surface (MS/BS, %) = (mineralizing surface length / total bone surface length) × 100% [19].

### 2.27 Histology and Immunofluorescence (IF) Assessment

Fixed femoral samples were decalcified at 10% EDTA for 28 days in preparation for histological and immunohistochemical (IHC) analysis. A 70%–100% ethanol series was followed by xylene clearing and paraffin embedding prior to sectioning. 4‐µm‐thick tissue slices were stained with H&E depending on standard methods. Primary antibodies against OCN, Perilipin, and 4‐HNE were incubated with slices for IF staining. A horseradish peroxidase detection system was utilized in line with the package recommendations to determine the immunoreactivity of the sections (Burlingame, CA, USA).

### 2.28 Statistical Analysis

Data are presented as mean ± SD. All experiments were independently repeated at least three times. Statistical analyses were performed using SPSS software (Armonk, NY, USA). Comparisons among multiple groups were carried out using one‐way analysis of variance (ANOVA) followed by Tukey's post hoc test. A *p*‐value < 0.05 was considered statistically significant.

## 3 Results

### 3.1 GCs Induce Ferroptosis of BMSCs Both In Vivo and In Vitro, and Aggravate the Death of BMSCs Derived From GIOP

To investigate the mechanisms underlying the development and progression of GIOP, we analyzed the single‐cell dataset GSE261072. As shown in Figure 1A, the cellular composition of mouse femurs in the GCs‐treated group exhibited marked changes compared with the control group, indicating that GCs treatment significantly altered the bone microenvironment. The major cell populations identified included mesenchymal lineage, erythroblasts, macrophages, neutrophils, HSCs, and cDCs (Figure 1A). Subsequently, GO enrichment analysis was performed on differentially expressed genes derived from all cell types, and revealed that lipid metabolism‐related pathways, such as lipid transport, lipid oxidation, and fatty acid metabolic processes, were significantly upregulated in the GCs group (Figure 1B). In addition, module score analysis of ferroptosis using the single‐cell database revealed a higher ferroptosis level in the GCs group, and feature plot analysis indicated that several classical ferroptosis factors also exhibited trends of alteration (Figure S1A,B). To further assess whether the observed lipid‐metabolic and ferroptosis‐related changes were specifically relevant to MSCs, we isolated the mesenchymal lineage cluster and performed differential expression analysis within this population. A bubble plot demonstrates that key ferroptosis regulators (Gpx4, Slc7a11, Nfe2l2, NCOA4) and lipid metabolism enzymes (Lpcat3, Por, Slc11a2) are significantly altered in MSCs upon GCs treatment (Figure S1C). DEX is a widely prescribed GCs used clinically as an immunosuppressive and anti‐inflammatory agent. Flow cytometry confirmed that DEX increased the percentage of Annexin V‐positive apoptotic BMSCs in a dose‐dependent manner (Figure S1D,E). As expected, DEX significantly suppressed osteogenic differentiation, mineralization, and osteogenic protein expression, while promoting adipogenic differentiation and adipogenic protein expression (Figure S2A–J). We then examined the protein expression levels of PTGS2 and ACSL4 in BMSCs treated with different concentrations of DEX. As characteristic markers of ferroptosis, both *Ptgs2* and *Acsl4* were significantly upregulated in a concentration‐dependent manner, and this finding was further supported by qPCR analysis (Figure 1C–E). MDA is a major product of lipid peroxidation, and the core features of ferroptosis are the excessive accumulation of lipid peroxides and iron deposition. As shown in Figure 1F,G, DEX treatment increased cellular MDA and iron levels in a dose‐dependent manner. Subsequently, the effect of various cell death inhibitors on ferroptosis mRNA (*Ptgs2*, *Acsl4*) and MDA levels was evaluated in BMSCs treated with 200 µm DEX. The results showed that the ferroptosis inhibitor Fer‐1 reversed the changes in MDA content as well as the mRNA levels of *Ptgs2* and *Acsl4*. In contrast, other forms of cell death inhibitors, including necrostatin‐1 (NEC‐1) (an inhibitor of necroptosis), Z‐VAD (an inhibitor of apoptosis), and 3‐methyladenine (3‐MA) (an inhibitor of autophagy), did not significantly alleviate cellular lipid peroxidation (Figure 1H,I). The GIOP mouse model was established as previously reported by our research group (Figure 1J) [3]. To assess iron content changes in GIOP mice, BMSCs were isolated from the control and GIOP groups, and iron levels were measured (Figure 1K). The results showed a significant increase in iron content in BMSCs from the GIOP group compared with the control group, while Fer‐1 treatment significantly reduced the iron level in vivo (Figure 1L). In subsequent MDA assays, we found that additional DEX treatment of BMSCs derived from GIOP mice further exacerbated MDA production, and this effect was alleviated by the ferroptosis inhibitor Fer‐1 (Figure 1M). Finally, micro‐CT and histological analysis were performed on the femurs of mice. As shown in Figure 1N and Figure S3A–D, the GIOP group exhibited a significant decrease in BV/TV, Tb.Th, and Tb.N, accompanied by an increase in Tb.Sp compared with the control group (Figure 1N and Figure S3A–D). These microstructural deteriorations were markedly ameliorated by treatment with the ferroptosis inhibitor Fer‐1 (Figure 1N and Figure S3A–D). H&E staining revealed a reduction in trabecular bone and a marked increase in bone marrow adiposity in the GIOP group (Figure 1O and Figure S3E). Meanwhile, tissue fluorescence results showed that ACSL4, a regulator of polyunsaturated fatty acid oxidation, was also significantly increased in the GIOP group; however, these phenotypes were alleviated by the use of Fer‐1 (Figure 1P and Figure S3F). In conclusion, these findings indicate that GCs promote ferroptosis in BMSCs both in vitro and in vivo, highlighting a critical role of ferroptosis in GIOP development.

**FIGURE 1:**
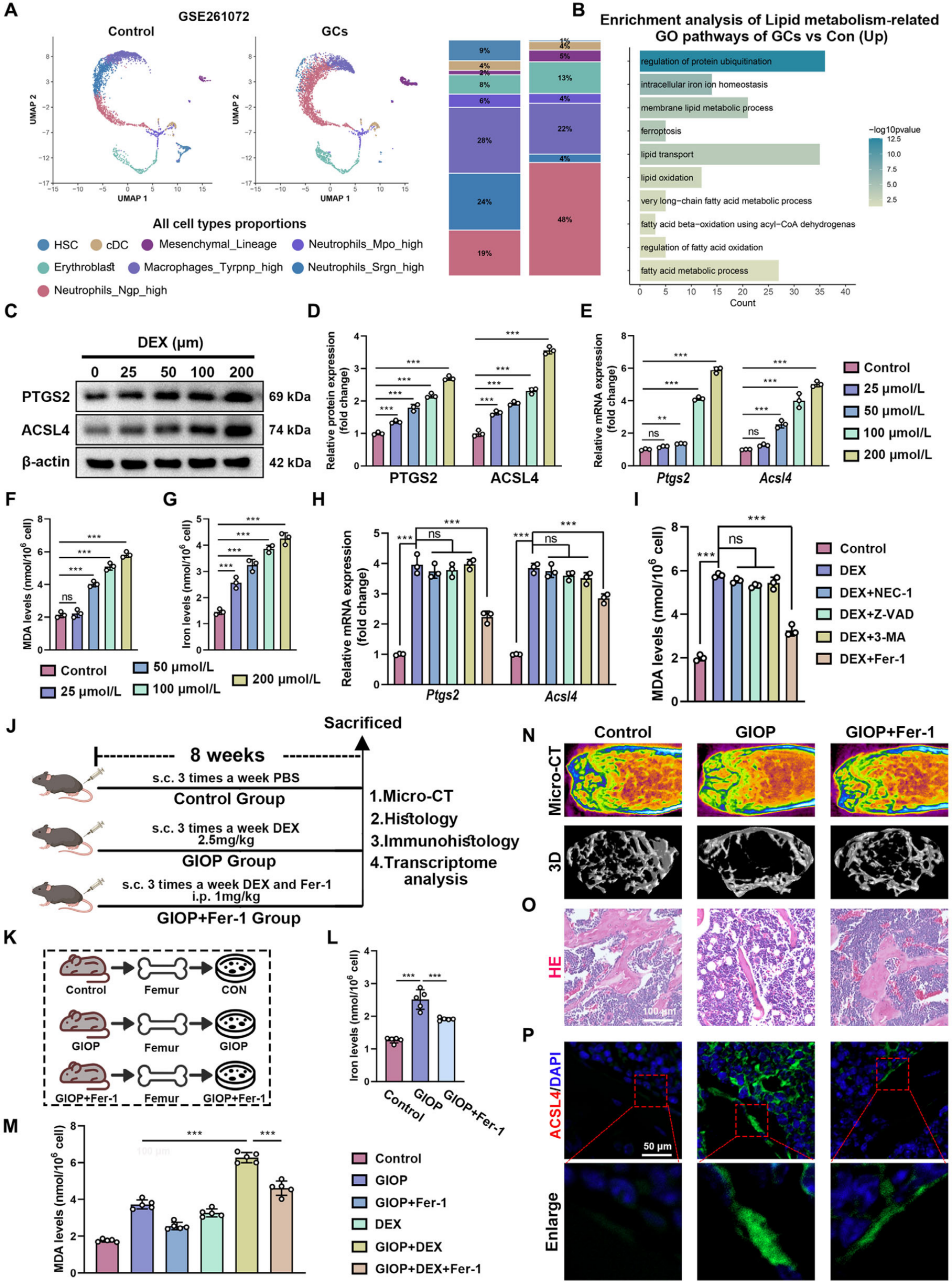
GCs induce ferroptosis in BMSCs and aggravate GIOP bone loss. (A) UMAP showing altered femur cell composition after GCs treatment. (B) GO enrichment of lipid metabolism‐related pathways in GCs group. (C–E) DEX dose‐dependently upregulates *Ptgs2* and *Acsl4* at protein and mRNA levels. (F, G) DEX dose‐dependently increases MDA and iron levels in BMSCs. (H, I) Fer‐1, but not other death inhibitors, reverses DEX‐induced MDA elevation and *Ptgs2* and *Acsl4* mRNA. (J) GIOP mouse model schematic. (K) Schematic diagram of primary BMSCs isolation from each group. (L, M) Iron and MDA are elevated in GIOP BMSCs, suppressed by Fer‐1. (N) Representative micro‐CT images (2D and 3D) of distal femur tissues from each group. (O) Representative images of H&E staining of distal femur tissues from each group. (P) Representative images of ACSL4 fluorescence staining of distal femur tissues from each group. Data are presented as the mean ± SD from 3 independent experiments. Statistical significance is denoted as ^*^ *p* < 0.05, ^**^ *p* < 0.01, ^***^ *p* < 0.001; ns indicates no significant difference.

### 3.2 Parkin Protects Against DEX‐Induced Ferroptosis In Vitro

To further explore the molecular mechanisms underlying GIOP development, we performed transcriptomic analysis on mouse femoral bone samples obtained from the CON and GIOP groups. The volcano plot in Figure 2A shows that a total of 57 180 genes were detected, among which 801 genes were significantly upregulated, and 397 genes were significantly downregulated after screening (Figure 2A). Subsequently, GO enrichment analysis of the differentially expressed genes was conducted. Figure 2B reveals significant differences between the two groups in biological processes such as mitochondrion, metabolism of lipids, and fatty acid metabolism (Figure 2B). Moreover, GSEA analysis showed that the ferroptosis pathway was significantly enriched in the GIOP group (Figure 2C). These transcriptomic results further support the findings presented in our first result section. Among the differentially expressed genes, we focused on Parkin, a well‐characterized E3 ubiquitin ligase with anti‐ferroptotic functions. Notably, the level of Parkin mRNA was markedly reduced in the GIOP group, and its protein and mRNA levels were decreased in BMSCs with DEX treatment (Figure 2A,D,E). Consistently, Parkin expression was also found to be significantly reduced in the femoral tissues of the GIOP group (Figure 2F,G). To accurately verify the regulatory role of Parkin in DEX‐induced ferroptosis of BMSCs, we knocked down Parkin using lentivirus‐mediated sh‐RNA (sh‐Parkin) and overexpressed Parkin using a specific plasmid (OE‐Parkin) in BMSCs (Figure S4A–D). Fluorescence staining with the lipid peroxidation probe C11 and the iron probe FerroOrange showed that knockdown of Parkin expression in vitro exacerbated intracellular lipid peroxidation levels and iron accumulation, while overexpression of Parkin significantly alleviated this trend (Figure 2H–K). MDA detection also showed that OE‐Parkin significantly reduced the elevation of MDA levels induced by DEX (Figure 2L). Subsequently, we assessed the differentiation potential of BMSCs using osteogenic mineralization staining and adipogenic staining. ALP, ARS, and Oil Red O staining revealed that knockdown of Parkin in vitro impaired osteogenic differentiation and shifted BMSCs toward adipogenic differentiation. Similarly, overexpression of Parkin significantly reversed this trend (Figure 2M–R). Finally, we examined the expression of ferroptosis‐, osteogenesis‐, and adipogenesis‐related proteins. Western blot analysis showed that Parkin knockdown promoted the expression of ferroptosis‐ and adipogenesis‐related proteins (PTGS2, ACSL4, Perilipin, and PPARγ), while inhibiting the expression of anti‐ferroptosis genes and osteogenic proteins (FTH1, GPX4, COL1, RUNX2, and OCN) (Figure 2S,T and Figure S5A–D). Taken together, these findings demonstrate that Parkin inhibits DEX‐induced ferroptosis in BMSCs.

**FIGURE 2:**
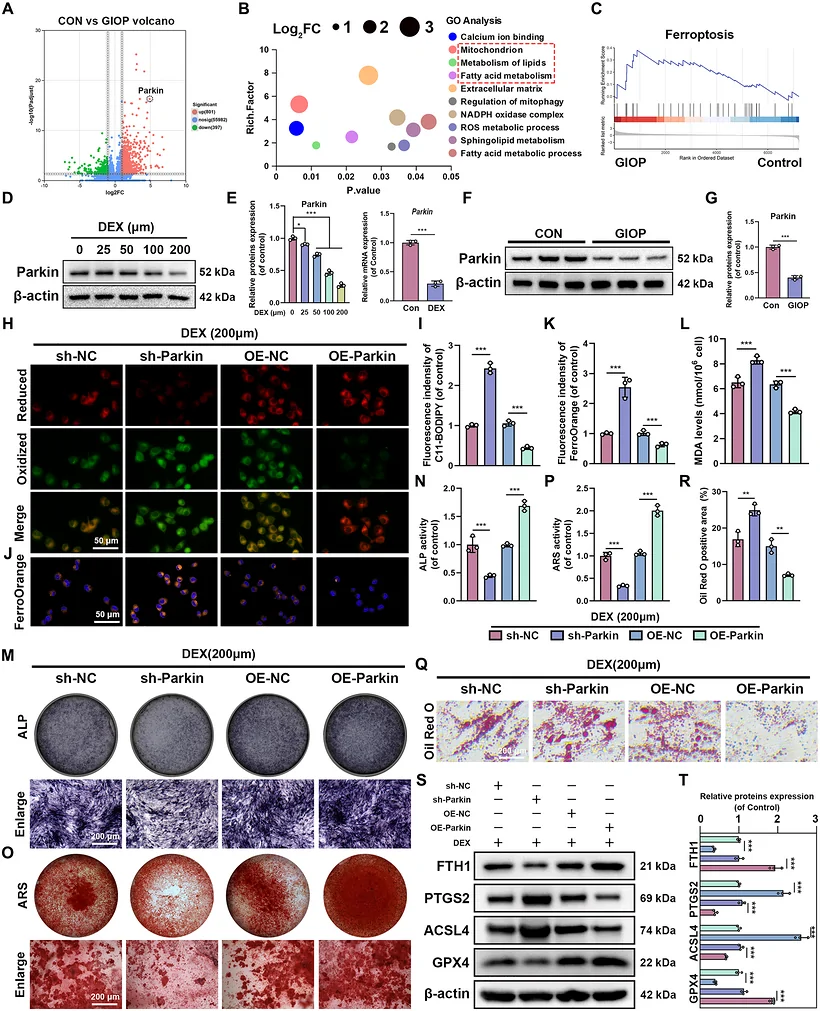
Parkin protects against DEX‐induced ferroptosis in BMSCs. (A) Volcano plot showing differentially expressed genes between control and GIOP groups (*n* = 4 per group). (B) GO enrichment analysis of DEGs, highlighting differences in mitochondrion, lipid metabolism, and fatty acid metabolism. (C) GSEA confirming significant enrichment of the ferroptosis pathway in the GIOP group. (D, E) DEX treatment reduces Parkin protein and mRNA levels in BMSCs in a dose‐dependent manner. (F, G) The Parkin protein level assay between the control and GIOP group by Western blot. To verify the effect of Parkin on ferroptosis and osteogenic/adipogenic differentiation of BMSCs, we set up the following four groups: DEX+sh‐NC, DEX+sh‐Parkin, DEX+OE‐NC, and DEX+OE‐Parkin. (H, I) Fluorescence images and quantitative analysis of C11 staining for lipid peroxidation in different treatment groups. (J, K) Fluorescence images and quantitative analysis of FerroOrange staining for Fe^2+^ in different treatment groups. (L) MDA quantification in different treatment groups. (M–R) ALP/ARS (osteogenic differentiation) and Oil Red O (adipogenic differentiation) staining and quantitative analysis in different treatment groups. (S, T) Western blot analysis of ferroptosis‐related (FTH1, PTGS2, ACSL4, GPX4) in different treatment groups. Data are presented as the mean ± SD from 3 independent experiments. Statistical significance is denoted as ^*^ *p* < 0.05, ^**^ *p* < 0.01, ^***^ *p* < 0.001; ns indicates no significant difference.

### 3.3 Parkin Regulates Mitochondrial Morphology and Dynamics Proteins in DEX‐Treated BMSCs

Given the critical role of mitochondrial functional integrity and morphological dynamics in the execution of ferroptosis, we next investigated whether Parkin regulates mitochondrial quality in DEX‐treated BMSCs. As mitochondria are the primary organelles responsible for intracellular ROS production, we found that sh‐Parkin exacerbated DEX‐induced cellular and mitochondrial ROS generation, whereas OE‐Parkin significantly alleviated this effect (Figure 3A–D). JC‐1 staining showed that DEX treatment reduced mitochondrial membrane potential, an effect that was aggravated by Parkin knockdown and rescued by Parkin overexpression (Figure 3E,F). Tom20 staining revealed that DEX induced mitochondrial fragmentation, which was exacerbated upon Parkin knockdown, whereas Parkin overexpression preserved an intact, elongated mitochondrial network (Figure 3G,H). To further explore the molecular basis of the observed mitochondrial morphological changes, we examined the expression levels of key mitochondrial dynamics‐related proteins by Western blot analysis. Consistent with the Tom20 staining results, DEX treatment significantly altered the expression of proteins governing mitochondrial fission and fusion. Specifically, DEX reduced the levels of the fusion‐promoting proteins OPA1, MFN1, and MFN2, while increasing the level of the fission‐promoting protein DRP1. sh‐Parkin further exacerbated these alterations, leading to a more pronounced decrease in fusion proteins and an increase in DRP1. In contrast, OE‐Parkin effectively reversed these changes, restoring OPA1, MFN1, and MFN2 expression and reducing DRP1 levels (Figure 3I,J). These results indicate that Parkin protects against DEX‐induced mitochondrial fragmentation by rebalancing the expression of fusion and fission regulators. Transmission electron microscopy is the gold standard for morphological detection of ferroptosis [20]. As shown in Figure 3K, DEX treatment induced typical ferroptotic mitochondrial features, including shrinkage and cristae loss. Parkin knockdown aggravated these abnormalities, while Parkin overexpression significantly alleviated them, indicating that Parkin preserves mitochondrial ultrastructure against DEX‐induced ferroptotic damage. Taken together, these results demonstrate that Parkin preserves mitochondrial functional integrity and normal morphology in DEX‐treated BMSCs by reducing mitochondrial ROS, restoring membrane potential, rebalancing mitochondrial dynamics, and preventing ferroptosis‐associated ultrastructural damage.

**FIGURE 3:**
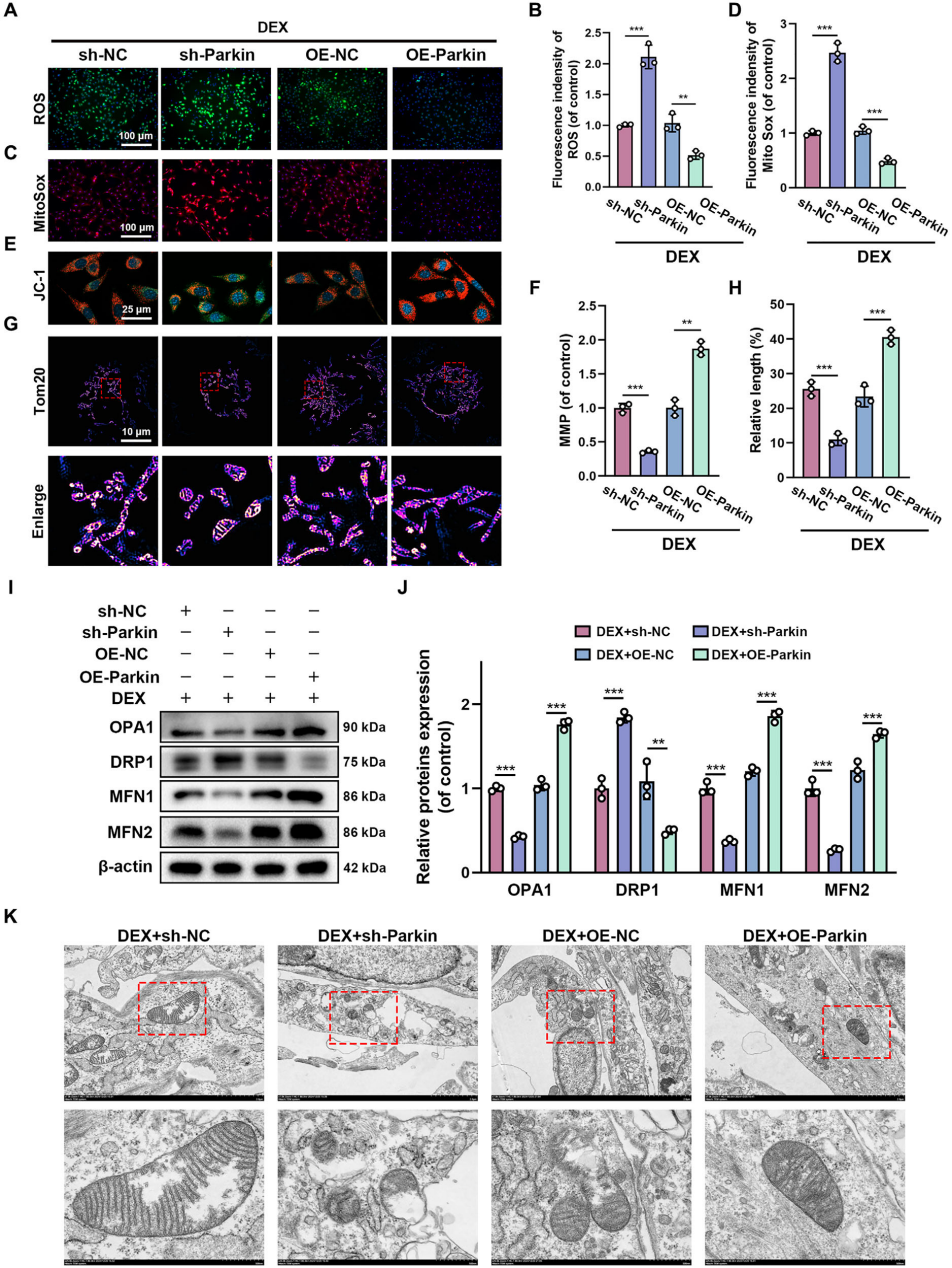
Parkin regulates mitochondrial morphology and dynamics in DEX‐treated BMSCs. To verify the effect of Parkin on mitochondria, we set up the following four groups: DEX+sh‐NC, DEX+sh‐Parkin, DEX+OE‐NC, and DEX+OE‐Parkin. (A, B) Representative images and quantitative analysis of DCFH‐DA staining in different groups. (C, D) Representative images and quantitative analysis of MitoSox staining in different groups. (E–F) Representative images and quantitative analysis of JC‐1 staining in different groups. (G, H) Representative images and quantitative analysis of Tom20 staining in different groups. (I, J) Western blots and quantitative analysis of fusion (OPA1, MFN1/2) and fission (DRP1) proteins. (K) Representative images of mitodrial TEM in different groups. Data are presented as the mean ± SD from 3 independent experiments. Statistical significance is denoted as ^*^ *p* < 0.05, ^**^ *p* < 0.01, ^***^ *p* < 0.001; ns indicates no significant difference.

### 3.4 Parkin Suppresses DEX‐Induced Ferroptosis via Modulation of PUFA‐Phospholipid Metabolism

Ferroptosis is driven by lipid peroxidation, and PUFA‐containing phospholipids in the cell membrane are the primary lipids susceptible to oxidation (Figure 4A) [21]. Notably, previous studies have demonstrated that phospholipids bearing PUFAs are more prone to oxidation than those containing saturated fatty acids. Based on this, we hypothesized that Parkin may inhibit DEX‐induced ferroptosis in BMSCs by regulating PUFA‐phospholipid metabolism. Consistent with the single‐cell GO enrichment analysis and transcriptomic KEGG analysis showing significant alterations in linolenic acid metabolism and glycerophospholipid metabolism in GIOP, we next focused on PUFA‐lipid metabolism in ferroptosis (Figure 4B). First, we examined the mRNA expression levels of key genes involved in the PUFA‐lipid metabolism pathway. qPCR results showed that overexpression of Parkin significantly reduced the mRNA levels of these key genes in the lipid metabolism pathway (Figure 4C). ACSL4 is known to regulate lipid biosynthesis, particularly through the preferential oxidation of phosphatidylethanolamine (PE) containing eicosatetraenoic acid. Therefore, we speculated that Parkin may regulate DEX‐induced PUFA content by targeting ACSL4. Consistent with our expectation, both DEX and the ferroptosis inducer Erastin markedly upregulated ACSL4 expression (Figure 4D,E). Similarly, we overexpressed ACSL4 to further verify its relationship with Parkin (Figure S4E,F). As shown in Figure 4F, compared with the DEX+OE‐NC group, DEX+OE‐Parkin markedly reduced the level of MDA. However, this reduction was significantly reversed by the simultaneous overexpression of ACSL4 (Figure 4F). Subsequently, we validated the effects of both molecules on DEX‐induced osteogenic and adipogenic differentiation of BMSCs. ALP/ARS and Oil Red O staining images showed that the positive effects of Parkin were inhibited by ACSL4 overexpression; that is, following OE‐ACSL4 treatment, osteogenic differentiation of BMSCs was weakened, while adipogenic differentiation was enhanced (Figure 4G–L). Finally, SPR experiments also confirmed an interaction between Parkin and ACSL4 (Figure 4M).

**FIGURE 4:**
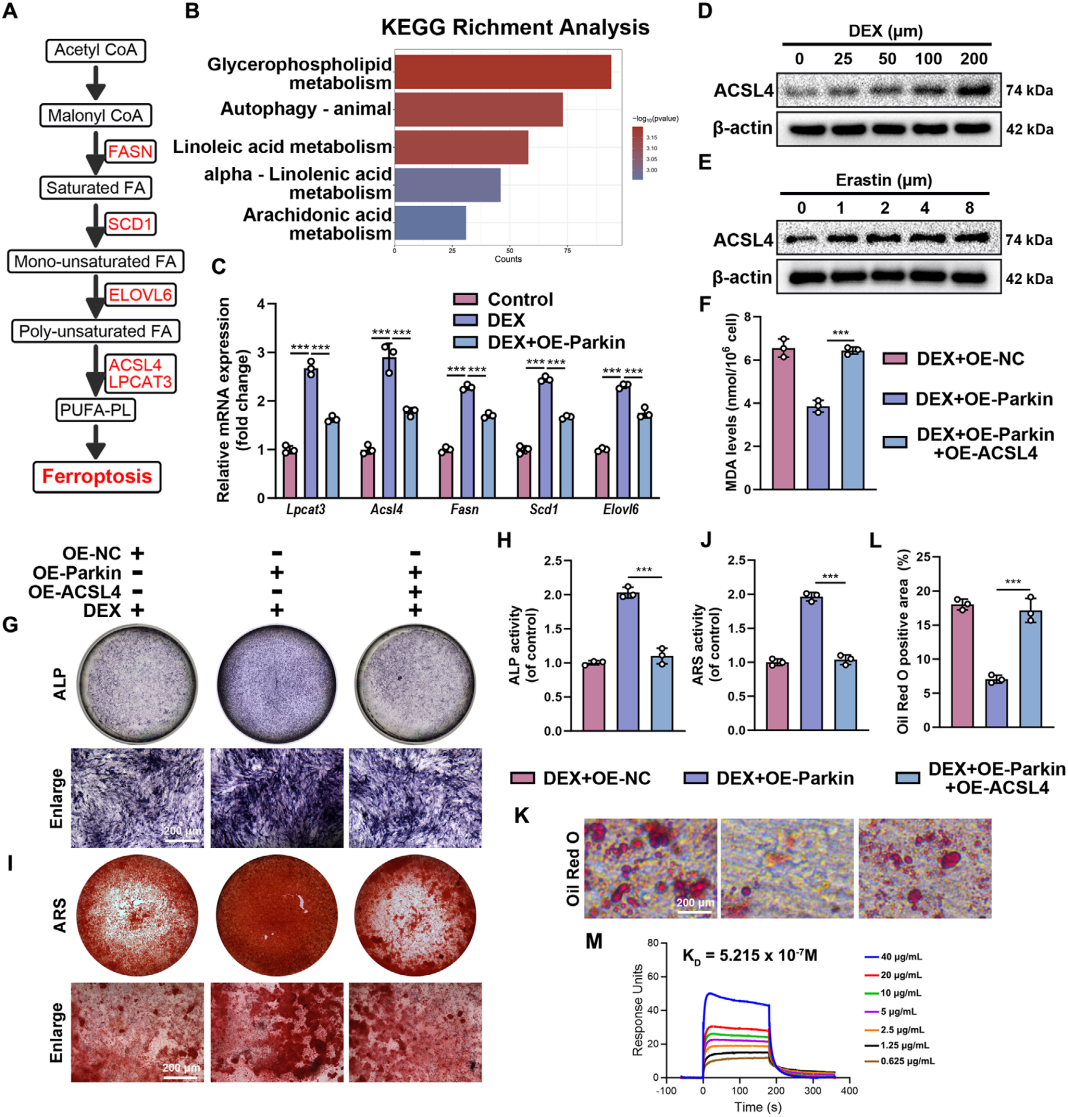
Parkin regulates PUFA‐phospholipid metabolism to inhibit ferroptosis. (A) Schematic illustration of PUFA‐phospholipid metabolism and its role in ferroptosis. (B) KEGG enrichment analysis showing pathways related to PUFA‐lipid metabolism in GIOP. (C) qPCR analysis of key lipid metabolism genes (*Lpcat3*, *Acsl4*, *Fasn*, *Scd1*, *Elovl6*). (D, E) Western blot showing ACSL4 upregulation by DEX and the ferroptosis inducer Erastin. (F) MDA levels in different groups. (G–L) ALP/ARS (osteogenic differentiation) and Oil Red O (adipogenic differentiation) staining and quantitative analysis in different treatment groups. (M) SPR sensorgrams confirming direct binding between Parkin and ACSL4 (K_D_ = 5.215 × 10^−^^7^ m). Data are presented as the mean ± SD from 3 independent experiments. Statistical significance is denoted as ^*^ *p* < 0.05, ^**^ *p* < 0.01, ^***^ *p* < 0.001; ns indicates no significant difference.

### 3.5 Parkin Binds to ACSL4 and Facilitates its Ubiquitination and Degradation

In subsequent experiments, we investigated the molecular mechanism by which Parkin acts on ACSL4. To verify whether exogenous Parkin interacts with ACSL4, we transfected HEK‐293T tool cells with Myc‐ACSL4, GFP‐Parkin, or GFP plasmids. The results showed that Parkin indeed interacts with ACSL4 (Figure 5A,B). To validate this in osteoblasts, we selected MC3T3‐E1 cells for subsequent IP experiments. We then examined whether endogenous Parkin interacts with endogenous ACSL4. When Parkin was overexpressed in MC3T3‐E1 cells, endogenous Parkin significantly bound to ACSL4, and Parkin overexpression led to increased levels of ACSL4 IP from MC3T3‐E1 cells (Figure 5C,D). Furthermore, in DEX‐treated MC3T3‐E1 cells, the amount of ACSL4 immunoprecipitated by Parkin was markedly reduced (Figure 5E,F). Previous studies have shown that Parkin functions as an E3 ubiquitin ligase in the ubiquitin‐proteasome‐dependent pathway. We investigated whether Parkin ubiquitinates ACSL4. Both endogenous and exogenous ubiquitination assays revealed that Parkin overexpression significantly enhanced the ubiquitination and degradation of ACSL4, whereas treatment with the proteasome inhibitor MG132 inhibited ubiquitination‐mediated ACSL4 degradation (Figure 5G–J and Figure S6A,B). Subsequently, we examined whether DEX suppresses ACSL4 ubiquitination. MC3T3‐E1 cells transfected with a vector expressing HA‐ubiquitin were treated with DEX. IP analysis showed that DEX treatment led to reduced ubiquitination levels of ACSL4, an effect that was reversed by treatment with the proteasome inhibitor MG132 (Figure S6C,D). Consistently, similar results were obtained in BMSCs, where Parkin overexpression promoted ACSL4 ubiquitination and degradation, further confirming the regulatory role of Parkin in ACSL4 ubiquitination (Figure S7A–J). K48‐linked and K63‐linked polyubiquitination are two common forms of polyubiquitination. Therefore, we investigated which type of polyubiquitination is triggered by the Parkin protein. The results showed that Parkin polyubiquitinates ACSL4 via wild‐type or K48 ubiquitin, but not K63 ubiquitin (Figure 5K,L). These findings indicate that Parkin induces polyubiquitination of ACSL4 primarily through K48‐linked ubiquitin chains. In contrast, the Parkin E3 ligase‐defective mutant negatively affected the ubiquitination process of ACSL4 and failed to effectively resist DEX‐induced ferroptosis (Figure 5M and Figure S6E). Finally, molecular docking further confirmed this: Parkin interacts with ACSL4 primarily through hydrogen bonding and other interactions involving residues such as GLN‐344, SER‐387, and GLN‐251 on Parkin and GLN‐578, LYS‐512, and LSY‐44 on ACSL4, with a strong binding energy of −12.5 kcal/mol (Figure 5N).

**FIGURE 5:**
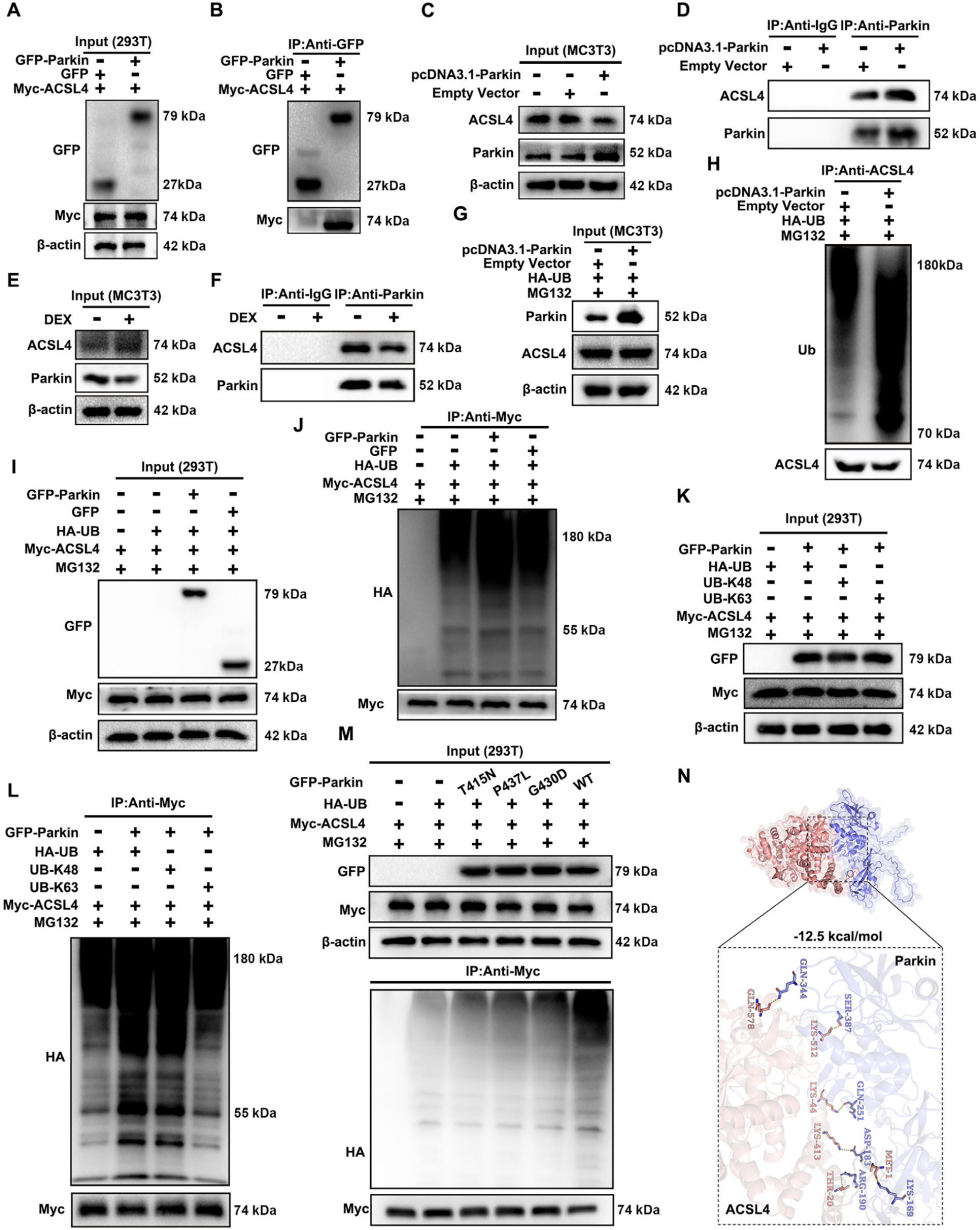
Parkin directly binds to ACSL4 and promotes its K48‐linked ubiquitination and proteasomal degradation. (A, B) Co‐IP in HEK‐293T cells confirming exogenous interaction between Parkin and ACSL4. (C, D) Co‐IP in MC3T3 cells validating endogenous interaction between Parkin and ACSL4. (E, F) DEX treatment weakens the Parkin‐ACSL4 interaction. (G, H) MC3T3 cells were transfected with HA‐ubiquitin along with Parkin overexpression plasmid or control PcDNA3.1, respectively. Ubiquitination of ACSL4 was analyzed by IP in MC3T3 cells. (I, J) HEK293T cells were transfected with Myc‐ACSL4 along with HA‐ubiquitin and GFP‐Parkin or GFP plasmids. The ubiquitination level of ACSL4 was analyzed by IP. (K, L) Parkin catalyzed polyubiquitination of the K48 linkage of ACSL4. HEK293T cells were transfected with Myc‐ACSL4 along with HA‐ubiquitin and GFP‐Parkin or K48 ubiquitin or K63 ubiquitin. Ubiquitination of ACSL4 was analyzed by IP. (M) HEK293 cells were transfected with Myc‐ACSL4 along with HA‐ubiquitin or GFP‐Parkin‐WT or GFP‐T415N or GFP‐P437L or GFP‐G430D plasmids. The ubiquitination level of ACSL4 was analyzed by IP. (N) Molecular docking reveals key interacting residues between Parkin and ACSL4.

### 3.6 Development and In Vitro Effects of Bone‐Targeted LNP Delivering Parkin mRNA

To date, mRNA‐based therapies have demonstrated great potential as an alternative platform for delivering therapeutic proteins. Due to their positive charge and resistance to nuclease degradation, LNPs have become the primary nanocarrier platform for therapeutic mRNA delivery [22]. Furthermore, precise drug delivery and the minimization of off‐target effects are critical for enhancing the clinical application potential of nucleic acid therapies. Previous studies have shown that coupling DSS6 to the surface of LNPs enables targeted delivery to bone tissue. Based on this, we propose to assemble LNPs with selective cell‐targeting functionality by fusing DSS6 with liposomes, thereby achieving specific targeting of bone tissue for therapeutic purposes in a GIOP model (Figure 6A). Subsequently, characterization analysis was performed on empty LNPs (Vector‐LNPs) and Parkin‐LNPs assembled with DSS6 (Parkin‐LNPs@DSS6) to evaluate their particle morphology and changes in zeta potential. TEM analysis revealed that, compared with Vector‐LNPs, the hybrid Parkin‐LNPs@DSS6 exhibited a larger particle size, and both types of particles displayed a nearly spherical morphology (Figure 6B,C). The hybridization of mRNA and DSS6 within the LNPs resulted in a shift of the zeta potential toward neutrality (Figure 6D). To evaluate the ability of mRNA transcripts to overcome challenges such as nuclease sensitivity, difficulties in antigen presentation, and effective carrier‐mediated delivery in vivo, agarose gel electrophoresis was used to examine the binding of mRNA to LNPs. The results confirmed that when the cationic liposome‐to‐mRNA ratio (w/w) exceeded 8:1, the mRNA was completely encapsulated (Figure 6E). Then, we further investigated their homing ability toward BMSCs in vivo. To evaluate the biodistribution of Parkin‐LNP@DSS6, ICG‐labeled Vector‐LNP (Vector‐LNP^ICG^) and Parkin‐LNP@DSS6 (Parkin‐LNP@DSS6^ICG^) were injected into 18 mice (divided into 2 groups, 9 mice per group). All mice were euthanized at 6, 24, or 48 h post‐injection, and their hindlimb, spleen, liver, kidney, and heart tissues were collected to measure fluorescence intensity. Parkin‐LNP@DSS6^ICG^ accumulated significantly in the lower limbs at 6 h and remained markedly accumulated after 48 h, demonstrating strong bone‐homing ability (Figure 6F and Figure S8A–C). In contrast, due to the lack of DSS6‐mediated targeting, Vector‐LNP showed the lowest penetration. High accumulation in the liver and kidneys at each time point indicated that these nanoparticles rely on hepatic mechanisms for the clearance of Parkin‐LNP@DSS6. The bone‐homing capability of DSS6‐modified LNPs was further supported by an in vitro hydroxyapatite (HA) binding assay, showing that DSS6‐LNP bound to HA with approximately 4.2‐ to 5.6‐fold higher efficiency than Vector‐LNP at 6–48 h (Figure S8D). Next, we verified whether the composite LNPs exert similar effects in vitro under DEX induction. As expected, Vector‐LNP had no regulatory effect on the Parkin‐ACSL4 axis, whereas Parkin‐LNP@DSS6 significantly increased the expression level of Parkin protein while markedly reducing the expression of ACSL4 (Figure 6G–I). Western blot analysis of the ferroptosis‐specific proteins PTGS2 and GPX4 showed that treatment with Parkin‐LNP@DSS6 significantly decreased PTGS2 protein levels and significantly increased the expression of the anti‐ferroptosis protein GPX4 (Figure 6J,K). Fluorescence images of the ferric ion probe FerroOrange and the lipid peroxidation probe C11 further supported this result, showing that Parkin‐LNP@DSS6 significantly reduced the level of DEX‐induced ferroptosis in BMSCs (Figure 6L–O). Subsequently, western blot and staining analyses were performed to assess the differentiation function of BMSCs. The results showed that Parkin‐LNP@DSS6 significantly increased the osteogenic proteins expression, restored the osteogenic differentiation and mineralization capacity of BMSCs inhibited by DEX, while attenuating their adipogenic differentiation (Figure 6P–W). Finally, in Co‐IP experiments, in vitro treatment with Parkin‐LNP@DSS6 also significantly promoted the ubiquitin‐mediated degradation of ACSL4 (Figure 6X,Y). In summary, we successfully constructed composite LNPs (Parkin‐LNP@DSS6) containing Parkin mRNA and capable of targeting bone tissue, which exhibit anti‐ferroptotic effects and restore the osteogenic differentiation and mineralization capacity of BMSCs under DEX induction.

**FIGURE 6:**
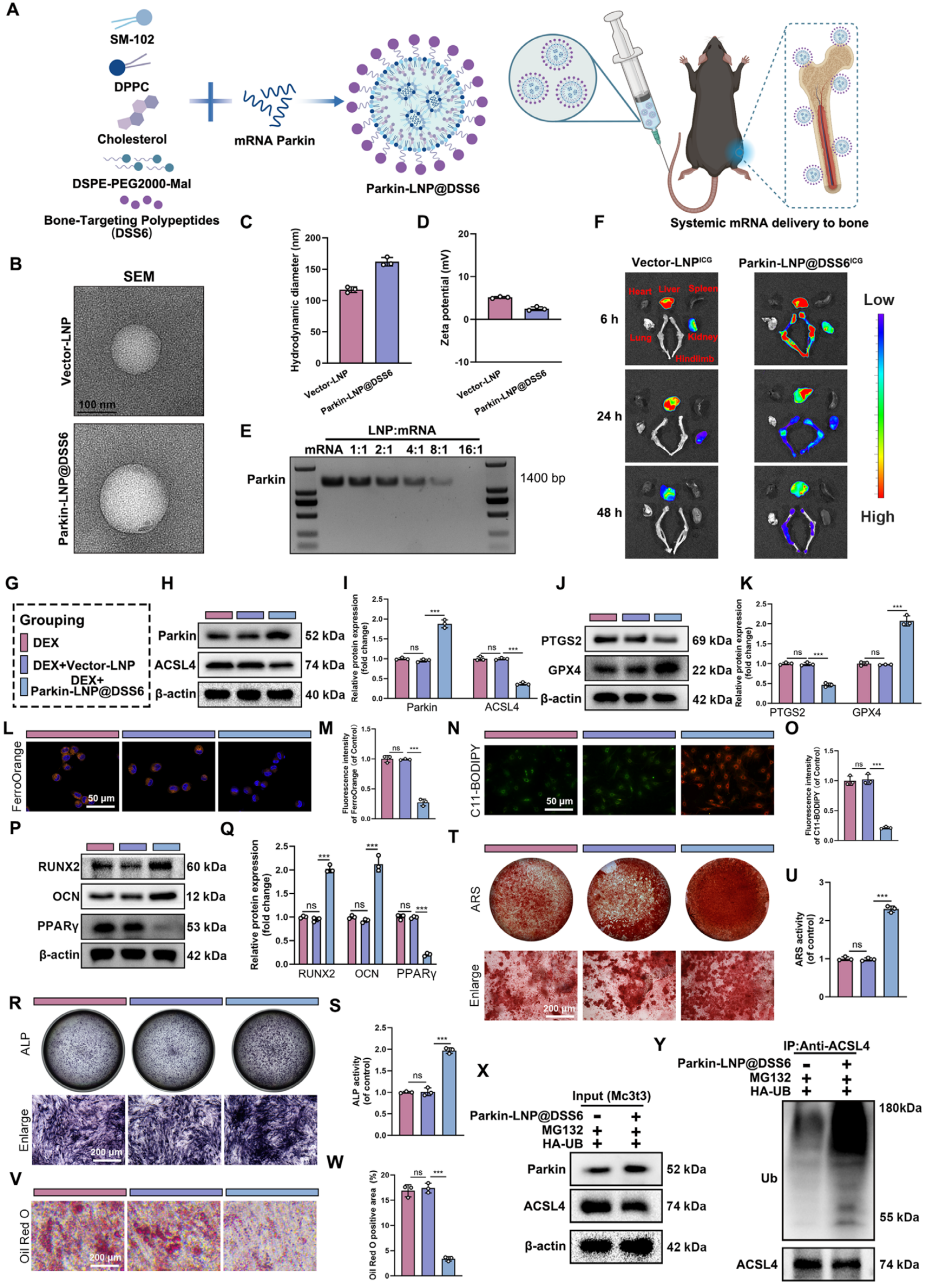
Development and in vitro effects of bone‐targeted Parkin‐LNP@DSS6. (A) Schematic diagram of the composition of Parkin‐LNP@DSS6. (B) TEM images showing the morphology of Vector‐LNP and Parkin‐LNP@DSS6. (C) Quantitative analysis of particle size of Vector‐LNP and Parkin‐LNP@DSS6. (D) Zeta potential measurements of Vector‐LNP and Parkin‐LNP@DSS6. (E) Agarose gel electrophoresis of the cationic liposome‐to‐mRNA ratio. (F) In vivo biodistribution of ICG‐labeled LNPs at 6, 24, and 48 h post‐injection. (G) In vitro experimental groups. (H–K) Western blot analysis of Parkin, ACSL4, PTGS2, and GPX4 in different groups. (L, M) Fluorescence images and quantitative analysis of FerroOrange staining for Fe^2+^ in different treatment groups. (N, O) Fluorescence images and quantitative analysis of C11 staining for lipid peroxidation in different treatment groups. (P, Q) Western blot analysis of RUNX2, OCN, and PPARγ in different treatment groups. (R–W) ALP/ARS (osteogenic differentiation) and Oil Red O (adipogenic differentiation) staining and quantitative analysis in different treatment groups. (X, Y) Ubiquitination of ACSL4 was analyzed by IP with Parkin‐LNP@DSS6 treatment in vitro. Data are presented as the mean ± SD from 3 independent experiments. Statistical significance is denoted as ^*^ *p* < 0.05, ^**^ *p* < 0.01, ^***^ *p* < 0.001; ns indicates no significant difference.

### 3.7 Bone‐Targeted LNP Delivering Parkin mRNA Ameliorates GIOP

To further investigate protective and therapeutic effects in vivo, researchers administered PBS, Fer‐1, or LNPs to DEX‐induced GIOP model mice and sham‐operated mice. Mice in the sham group and the GIOP‐only group received intravenous injections of PBS, Vector‐LNP, or Parkin‐LNP@DSS6 every three days for 4 weeks. Fer‐1 was administered intraperitoneally following the same dosing regimen (every three days for 4 weeks) to evaluate its therapeutic efficacy (Figure 7A). The specific grouping is shown in Figure 7B. Micro‐CT results showed that, compared with the sham and GIOP groups, the empty LNP group (Vector‐LNP) exhibited no significant therapeutic effect on bone microarchitecture. Fer‐1 treatment slightly improved the bone microarchitecture in the GIOP group, whereas the group receiving intravenous injection of Parkin‐LNP@DSS6 showed a significantly improved bone quality phenotype, characterized by higher BV/TV, Tb.Th, and Tb.N, along with a lower Tb.Sp (Figure 7C–H). H&E staining indicated that GIOP increased the formation of lipid vacuoles in vivo, whereas Parkin‐LNP@DSS6 (but not Vector‐LNP) reversed this trend (Figure 7I,J). Furthermore, compared with other treatment regimens, calcein double‐labeling fluorescence staining of femurs showed that the Parkin‐LNP@DSS6 treatment group promoted new bone formation in mouse femurs, enhanced the expression of the osteogenic key protein OCN, and inhibited the production of the adipogenic protein Perilipin (Figure 7K–P). 4‐HNE is a classic product of lipid metabolism [23]. Fluorescence staining of femoral tissue showed that Parkin‐LNP@DSS6 significantly suppressed the elevated lipid metabolism level in the GIOP group and reduced 4‐HNE expression (Figure 7Q,R). Long‐term hormone therapy not only causes GIOP but also leads to steroid‐induced osteonecrosis of the femoral head (SONFH) [24]. We also validated the role of Parkin‐LNP@DSS6 in SONFH. Micro‐CT analysis showed that the bone mineral content in the subchondral region of the femoral head was significantly reduced, and the trabecular bone structure was deteriorated in the GIOP group. In contrast, both the GIOP+Fer‐1 group and the GIOP+Parkin‐LNP@DSS6 group exhibited significant improvements in these pathological parameters. Qualitative parameters, including Tb.N, Tb.Th, and BV/TV were significantly higher in these two groups than in the dexamethasone group, while Tb.Sp was reduced (Figure S9A–F). Figure S10 shows that Parkin‐LNP@DSS6 treatment did not cause any apparent histological changes in the heart, lung, liver, spleen, or kidney compared with the control group. Moreover, serum alanine aminotransferase (ALT), aspartate aminotransferase (AST) and blood urea nitrogen (BUN) levels remained unchanged, indicating no detectable hepatotoxicity. These data confirm the biosafety of Parkin‐LNP@DSS6 in vivo. Collectively, these experimental results demonstrate that the Parkin‐LNP@DSS6 selective delivery system achieves stable bone‐targeted delivery and exerts anti‐GIOP effects.

**FIGURE 7:**
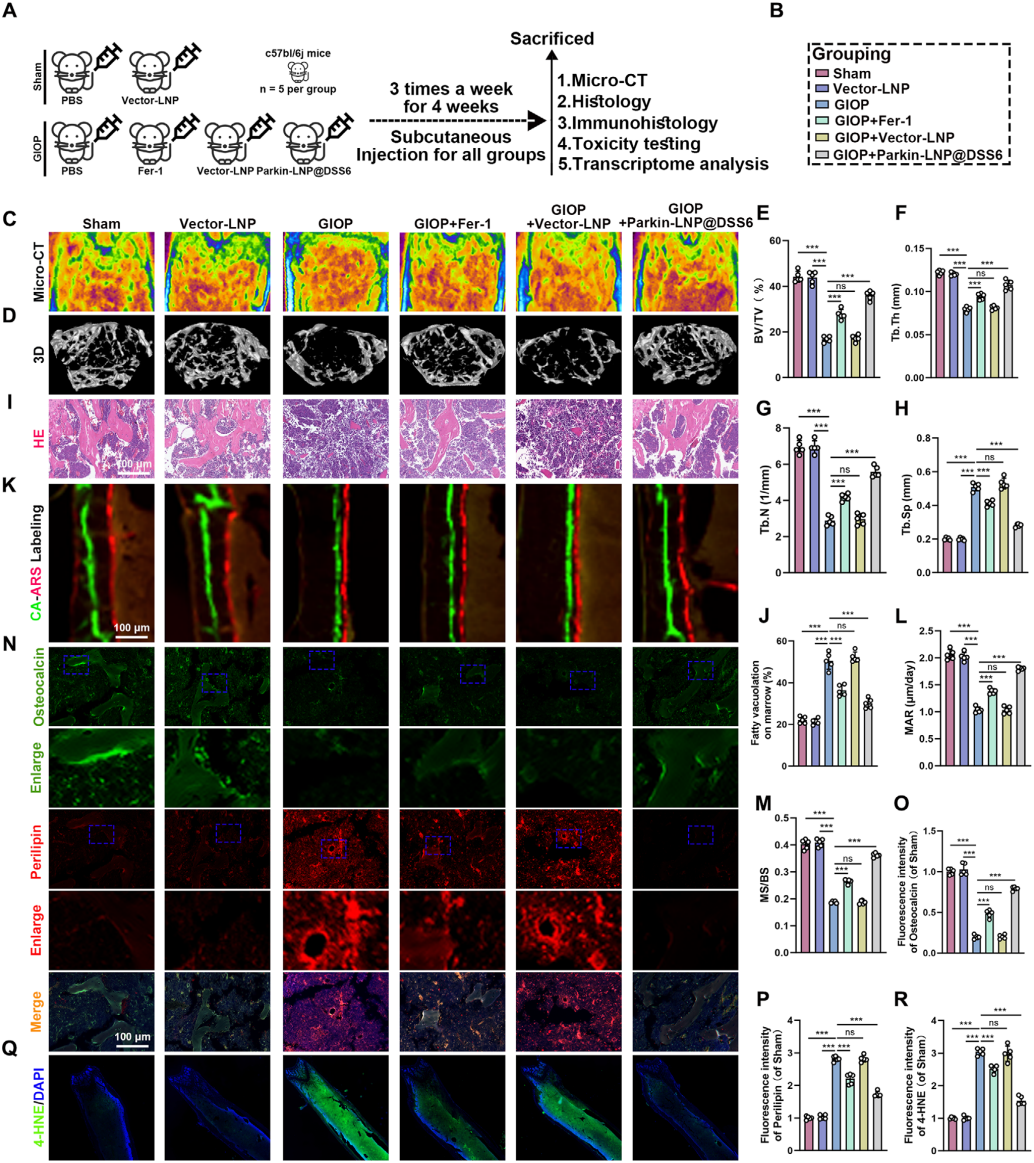
Bone‐targeted Parkin‐LNP@DSS6 ameliorates GIOP in vivo. To evaluate the in vivo effects of the composite LNP, the following six groups were established: Sham, Vector‐LNP, GIOP, GIOP+Fer‐1, GIOP+Vector‐LNP, and GIOP+Parkin‐LNP@DSS6. (A) Schematic illustration of the in vivo experimental design and treatment regimen. (B) Grouping information for sham, GIOP model, and treatment groups. (C–H) Representative micro‐CT images (2D and 3D) and quantitative analysis of trabecular bone parameters (BV/TV, Tb.Th, Tb.N, Tb.Sp) in distal femurs. (I, J) Representative images and quantitative analysis of H&E staining of distal femurs from each group. (K–M) Representative images and quantitative analysis of calcein double‐labeling fluorescence staining of distal femurs from each group. (N–P) Representative images and quantitative analysis of OCN and Perilipin fluorescence staining of distal femurs from each group. (Q, R) Representative images and quantitative analysis of 4‐HNE fluorescence staining of distal femurs from each group. Data are presented as the mean ± SD from 5 independent experiments. Statistical significance is denoted as ^*^ *p* < 0.05, ^**^ *p* < 0.01, ^***^ *p* < 0.001; ns indicates no significant difference.

### 3.8 Transcriptomic Profiling of Bone‐Targeted Parkin mRNA LNP Treatment in GIOP

The above analyses indicate that this composite LNP significantly inhibits DEX‐induced ferroptosis in BMSCs both in vivo and in vitro. To explore its mechanism of action, we collected femoral tissue samples from the GIOP+Parkin‐LNP@DSS6 group and the GIOP group and performed transcriptome sequencing analysis. The volcano plot showed significantly differentially expressed genes between the two groups, and the gene heatmap revealed 325 upregulated genes and 149 downregulated genes among the differentially expressed genes (Figure 8A,B). Subsequently, we performed KEGG and GO enrichment analyses on the differentially expressed genes. KEGG enrichment analysis showed significant differences between the two groups in signaling pathways such as mitophagy, ferroptosis, and the p53 signaling pathway, while GO enrichment analysis revealed differences in biological processes, including lipid transport and cellular lipid metabolic processes (Figure 8C,D). The GO Circular Plot illustrated specific differentially expressed genes involved in these significantly distinct biological processes (Figure 8E). To validate these findings, we performed GSEA, the results of which were consistent with the KEGG and GO analyses, showing that the treatment group significantly inhibited ferroptosis and fatty acid metabolism while promoting osteoblast formation (Figure 8F–H). Finally, we also performed tissue Western blot analysis on femoral tissues, and the results showed that GIOP+Parkin‐LNP@DSS6 treatment inhibited the production of ACSL4 and PTGS2 while promoting the expression of Parkin and OCN, which is consistent with our in vitro findings (Figure 8I,J). Overall, these results demonstrate that Parkin‐LNP@DSS6, as a composite LNP, represents a promising strategy for the treatment of GIOP. It regulates fatty acid metabolism by modulating the Parkin/ACSL4 pathway, thereby inhibiting ferroptosis, providing new insights and approaches for the treatment of hormone‐related bone metabolic diseases (Figure 9).

**FIGURE 8:**
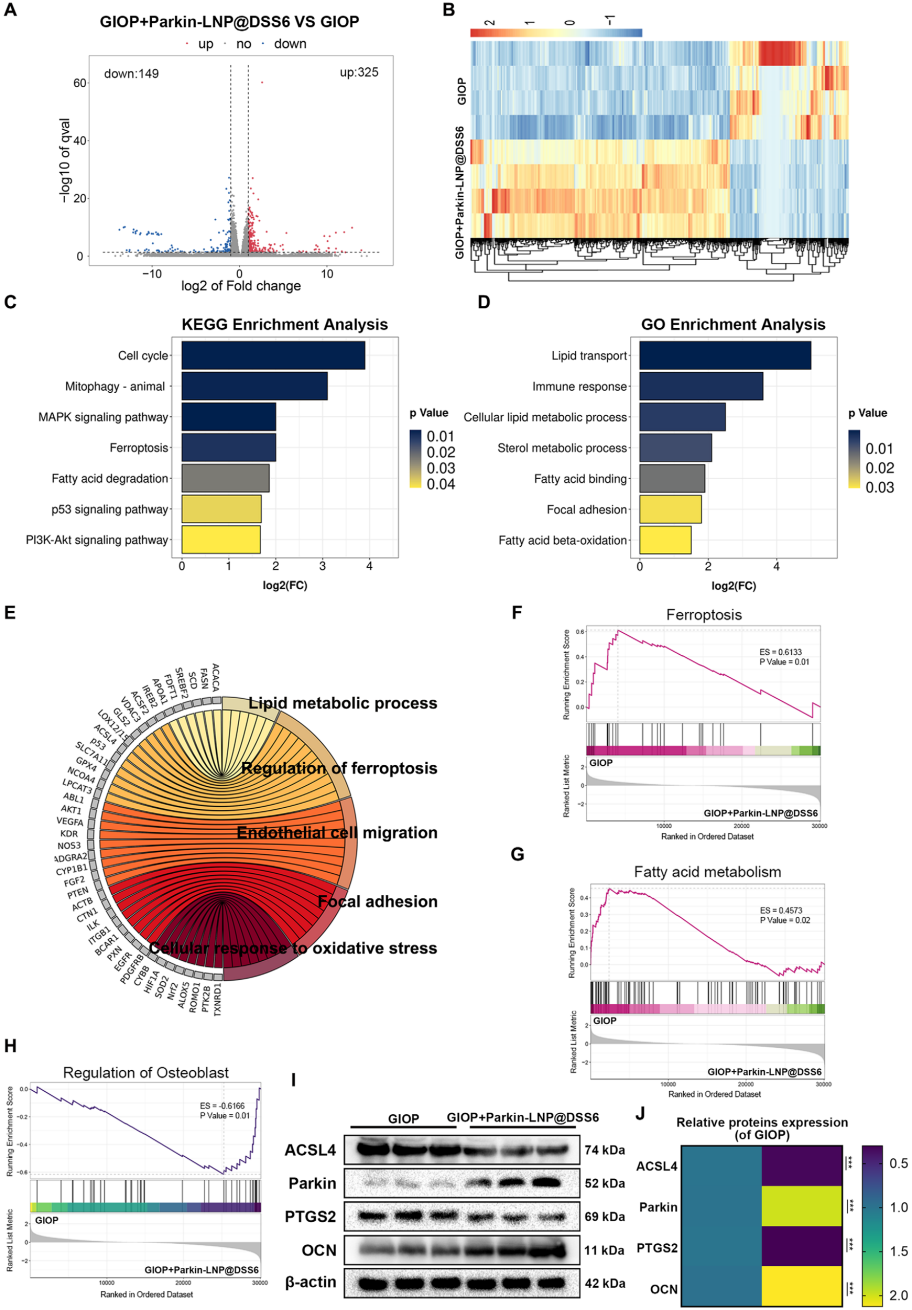
Transcriptomic profiling reveals that Parkin‐LNP@DSS6 suppresses ferroptosis and fatty acid metabolism in GIOP. Transcriptomic analysis was performed on femoral tissues from the GIOP group and the GIOP+Parkin‐LNP@DSS6 group (*n* = 4). (A) Volcano plot showing DEGs between the GIOP and GIOP+Parkin‐LNP@DSS6 groups. (B) Heatmap of DEGs: 149 upregulated and 325 downregulated genes in the treatment group. (C) KEGG enrichment analysis of DEGs. (D) GO enrichment analysis of DEGs. (E) GO Circular Plot illustrating specific DEGs involved in the significantly enriched biological processes. (F–H) GSEA analysis of GIOP and GIOP+Parkin‐LNP@DSS6 groups. (I, J) Western blot analysis of ACSL4, Parkin, PTGS2, and OCN between the GIOP and GIOP+Parkin‐LNP@DSS6 groups. Data are presented as the mean ± SD from 3 independent experiments. Statistical significance is denoted as ^*^ *p* < 0.05, ^**^ *p* < 0.01, ^***^ *p* < 0.001; ns indicates no significant difference.

**FIGURE 9:**
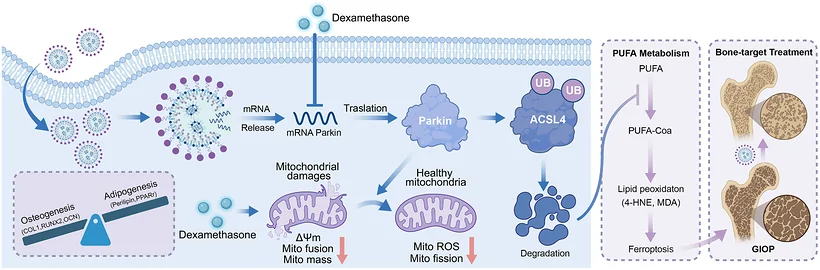
GCs reduce Parkin, leading to ACSL4 accumulation and PUFA‐phospholipid‐driven ferroptosis in BMSCs, which impairs osteogenesis and promotes adipogenesis, causing GIOP. Parkin restoration (via OE‐Parkin or Parkin‐LNP@DSS6) ubiquitinates and degrades ACSL4, inhibiting ferroptosis, rescuing bone formation, and rescues GIOP bone loss.

## 4 Discussion

This study reveals for the first time the key molecular mechanism by which Parkin targets and degrades ACSL4 through K48‐linked polyubiquitination, thereby inhibiting DEX‐induced ferroptosis in BMSCs and effectively alleviating GIOP. On this basis, we successfully constructed DSS6‐modified bone‐targeted lipid nanoparticles (Parkin‐LNP@DSS6) for delivering Parkin mRNA and validated their therapeutic potential in anti‐ferroptosis, pro‐osteogenic differentiation, and bone microstructural repair in both in vivo and in vitro models. These findings not only deepen our understanding of the pathogenesis of GIOP but also provide a novel therapeutic strategy for hormone‐related bone metabolic diseases.

Chronic or high‐dose GCs therapy is a leading cause of secondary osteoporosis, imposing a significant burden on patients’ daily living and substantially increasing their susceptibility to osteoporotic fractures [25]. Traditional views hold that GCs primarily induce bone loss by inhibiting osteogenic differentiation of BMSCs, promoting their adipogenic differentiation, and inducing apoptosis and autophagic dysregulation [26]. However, accumulating evidence in recent years indicates that ferroptosis, an iron‐dependent, lipid peroxidation‐driven form of regulated cell death, plays an important role in various bone metabolic diseases. For example, Jiang et al. found that ferroptosis is involved in the progression of postmenopausal osteoporosis, while Liu et al. reported that ferroptosis plays a key role in cartilage degeneration in osteoarthritis [11, 27]. Furthermore, although our team previously discovered that inhibiting DEX‐induced ferroptosis in osteocytes by stabilizing SLC7A11 delays GIOP progression, the ferroptosis induced by DEX in BMSCs and its related mechanisms had not yet been elucidated [3].

This study systematically demonstrates for the first time that GCs induce typical ferroptosis in BMSCs, characterized by upregulation of PTGS2 and ACSL4, accumulation of MDA and iron ions, mitochondrial shrinkage, and cristae reduction, all of which were significantly reversed by the ferroptosis inhibitor Fer‐1. Moreover, ACSL4 expression was elevated in femoral tissues of GIOP model mice, accompanied by bone microstructural destruction and bone marrow adiposity. These results integrate ferroptosis into the pathological network of GIOP, expanding the mechanistic understanding of this disease.

Parkin, an E3 ubiquitin ligase initially identified in association with Parkinson's disease, has recently been shown to exert broad protective functions in cardiovascular diseases, tumors, and metabolic diseases [12]. Specifically regarding ferroptosis regulation, Li et al. reported that Parkin inhibits cardiomyocyte ferroptosis by promoting ACSL4 ubiquitination, while Wang et al. found that Parkin‐mediated mitophagy alleviates neuronal ferroptosis [14, 28]. However, the role of Parkin in bone metabolism, particularly in GIOP, has not been previously reported. In this study, we found that Parkin expression was significantly downregulated in both bone tissues of GIOP mice and DEX‐treated BMSCs. Functional experiments demonstrated that Parkin knockdown exacerbated DEX‐induced lipid peroxidation, iron accumulation, and loss of mitochondrial membrane potential, while also impairing the osteogenic capacity and enhancing the adipogenic differentiation of BMSCs. In contrast, Parkin overexpression significantly reversed these phenotypes. Notably, we further discovered that Parkin maintains mitochondrial network integrity by regulating mitochondrial dynamics proteins (restoring OPA1, MFN1, and MFN2 expression while reducing DRP1 levels), which is consistent with the mitochondrial protective functions of Parkin reported in other cell types but is reported here for the first time in BMSCs. ACSL4 is a core rate‐limiting enzyme in the ferroptosis pathway, catalyzing the CoAylation of PUFAs and their incorporation into membrane phospholipids, thereby providing substrates for lipid peroxidation [29]. Wu et al. and Ding et al. have successively demonstrated that ACSL4 is an essential molecule for ferroptosis execution [30, 31]. In this study, through multiple lines of evidence, including Co‐IP, ubiquitination assays, and molecular docking, we confirmed that Parkin directly binds to ACSL4 and promotes its proteasome‐dependent degradation via K48‐linked polyubiquitination. Consistently, the Parkin E3 ligase activity‐deficient mutant failed to effectively promote ACSL4 degradation or resist DEX‐induced ferroptosis. Furthermore, DEX treatment significantly weakened the interaction between Parkin and ACSL4 and reduced the ubiquitination level of ACSL4, suggesting that GCs may promote ACSL4 accumulation and thereby drive ferroptosis by downregulating Parkin expression or interfering with its enzymatic activity. ACSL4 overexpression reversed the protective effects of Parkin on ferroptosis and osteogenic differentiation, further establishing the central regulatory role of the Parkin‐ACSL4 axis in GIOP‐associated BMSC ferroptosis.

Based on the above mechanism, we developed DSS6‐modified bone‐targeted Parkin mRNA‐LNPs. In recent years, mRNA‐LNP technology has gained significant attention due to the success of COVID‐19 vaccines, yet its application in bone tissue engineering is still in its infancy [32]. Previous studies have achieved bone‐targeted delivery of small molecules or siRNA using DSS6 modification, but bone‐targeted delivery of mRNA has rarely been reported. In this study, Parkin‐LNP@DSS6 exhibited a nearly spherical morphology, completely encapsulated the mRNA, and demonstrated excellent bone‐homing ability in vivo. In DEX‐induced BMSCs, this nanoparticle significantly upregulated Parkin, downregulated ACSL4, inhibited ferroptosis, and restored osteogenic differentiation capacity. In a GIOP mouse model, intravenous injection of Parkin‐LNP@DSS6 significantly improved bone microstructural parameters (BV/TV, Tb.N, Tb.Th), reduced bone marrow lipid vacuoles and the lipid peroxidation product 4‐HNE, and promoted new bone formation. Notably, this treatment also exhibited protective effects against SONFH, suggesting its potential broad‐spectrum applicability. Transcriptome sequencing further confirmed that the ferroptosis and fatty acid metabolism pathways were significantly inhibited in the treatment group, while osteogenesis‐related pathways were enriched, which is highly consistent with our in vitro results.

Several limitations exist in this study. Despite bone‐targeting modification, a portion of Parkin‐LNP@DSS6 accumulates in the liver and kidneys, indicating potential off‐target effects that need further mitigation. The therapeutic observation was limited to four weeks; long‐term efficacy, safety, and immunogenicity of repeated dosing remain unexplored. Additionally, clinical translation of mRNA‐LNPs faces hurdles including manufacturing scalability, storage conditions, and regulatory requirements. Future work should address these issues to facilitate clinical development. Additionally, although osteoclasts play an important role in bone remodeling, the present study focused on BMSCs because GCs‐induced bone loss is primarily driven by impaired bone formation. The potential impact of Parkin‐LNP@DSS6 on osteoclast ferroptosis and bone resorption was not investigated and warrants further study.

In summary, this study elucidates a novel mechanism by which Parkin inhibits BMSC ferroptosis and alleviates GIOP through ubiquitination‐mediated degradation of ACSL4, and provides an innovative therapeutic strategy based on bone‐targeted mRNA delivery. This strategy not only offers a new approach for the treatment of GIOP but also opens new avenues for the intervention of other ferroptosis‐related bone metabolic diseases, such as SONFH and postmenopausal osteoporosis. Future research should focus on further optimizing the LNP formulation, as well as exploring its long‐term safety and translational feasibility.

## 5 Conclusion

In summary, this study demonstrates that Parkin alleviates GIOP by promoting K48‐linked ubiquitination and proteasomal degradation of ACSL4, thereby inhibiting BMSC ferroptosis, restoring osteogenic differentiation, and reducing bone loss. Furthermore, a bone‐targeted LNP delivering Parkin mRNA (Parkin‐LNP@DSS6) effectively ameliorates GIOP, providing a promising therapeutic strategy for hormone‐related bone metabolic diseases.

## Author Contributions

**Li‐jiang Han, Jian‐sen Miao, Yi‐ting Tu, and Jian Xiao**: data curation, formal analysis, writing – original draft, and visualization. **Yi‐feng Shi and Yi‐ting Tu**: conceptualization, methodology, and data curation. **Yu‐zhe Lin and Bing‐hao Lin**: data curation, visualization, and software. **Hua‐lin Li and Xuan‐qi Zheng**: methodology and data curation. **Hong‐qiang Wu and Lin‐jie Chen**: formal analysis and methodology. **Ai‐min Wu, Zhen Lin, Jian Xiao, and Hai‐xiao Liu**: validation and software. **Chen Jin and Gang Zheng**: supervision, writing – review, and editing. **Chen Jin, Hong‐qiang Wu, Jian Xiao, and Lei Yang**: funding acquisition, supervision, writing – review, and editing.

## Conflicts of Interest

The authors declare no conflicts of interest.

## Supporting information




**Supporting File 1**: advs76586‐sup‐0001‐SuppMat.docx.

**Supporting File 2**: advs76586‐sup‐0002‐Data.zip.

## Data Availability

The data that support the findings of this study are available from the corresponding author upon reasonable request.

## References

[1] D. Czock , F. Keller , F. M. Rasche , and U. Häussler , “Pharmacokinetics and Pharmacodynamics of Systemically Administered Glucocorticoids,” Clinical Pharmacokinetics 44, no. 1 (2005): 61–98, 10.2165/00003088-200544010-00003. 15634032

[2] A. Rahman and M. F. Haider , “A Comprehensive Review on Glucocorticoids Induced Osteoporosis: A Medication Caused Disease,” Steroids 207 (2024): 109440, 10.1016/j.steroids.2024.109440. 38754651

[3] Y. Shi , Q. Tang , S. Sheng , et al., “PSMD14 Stabilizes SLC7A11 to Ameliorate Glucocorticoid‐Induced Osteoporosis by Suppressing Osteocyte Ferroptosis,” Advanced Science 12, no. 31 (2025): 14902, 10.1002/advs.202414902. PMC12376700 40444470

[4] M. Li , W. Zhu , M. Hu , et al., “Dynamic Profiling of BMSC‐dECM Reveals Accumulation of Core Matrisome Proteins Suppresses Osteogenic Differentiation and Bone Regeneration,” Journal of Advanced Research 80 (2026): 393–410, 10.1016/j.jare.2025.05.023. PMC12869295 40349957

[5] Y. Zhang , Y. Deng , W. Yao , K. Xia , L. Zhang , and G. Wang , “microRNA‐576‐5p Ameliorates Dexamethasone‐Induced BMSC Injury by Suppressing ANXA2,” Scientific Reports 15, no. 1 (2025): 30612, 10.1038/s41598-025-16883-9. PMC12368243 40836105

[6] H. Kang , H. Chen , P. Huang , et al., “Glucocorticoids Impair Bone Formation of Bone Marrow Stromal Stem Cells by Reciprocally Regulating microRNA‐34a‐5p,” Osteoporosis International 27, no. 4 (2016): 1493–1505, 10.1007/s00198-015-3381-x. 26556739

[7] Y. Zhang , W. Si , Y. Mao , et al., “Upregulation of Ferroptosis in Glucocorticoids‐Induced Posterior Subcapsular Cataracts,” Communications Biology 8, no. 1 (2025): 613, 10.1038/s42003-025-08067-y. PMC12000516 40234585

[8] D. Wang , C. Zhang , F. Yang , et al., “AMPK‐ULK1‐Mediated Ferritinophagy Drives Ferroptosis in GLA‐Induced Testicular Toxicity,” Research 8 (2025): 0860, 10.34133/research.0860. PMC12377500 40862056

[9] C. Jin , D. P. Zhang , Z. Lin , et al., “Piezo1‐Mediated Ferroptosis Delays Wound Healing in Aging Mice by Regulating the Transcriptional Activity of SLC7A11 Through Activating Transcription Factor 3,” Research 8 (2025): 0718. 10.34133/research.0718 PMC12133029 40463502

[10] Y. Chen , W. Zhao , A. Hu , et al., “Type 2 Diabetic Mellitus Related Osteoporosis: Focusing on Ferroptosis,” Journal of Translational Medicine 22, no. 1 (2024): 409, 10.1186/s12967-024-05191-x. PMC11064363 38693581

[11] Z. Jiang , G. Qi , X. He , et al., “Ferroptosis in Osteocytes as a Target for Protection against Postmenopausal Osteoporosis,” Advanced Science 11, no. 12 (2024): 2307388, 10.1002/advs.202307388. PMC10966575 38233202

[12] D. P. Narendra and R. J. Youle , “The Role of PINK1–Parkin in Mitochondrial Quality Control,” Nature Cell Biology 26, no. 10 (2024): 1639–1651, 10.1038/s41556-024-01513-9. 39358449

[13] T. N. Nguyen , B. S. Padman , and M. Lazarou , “Deciphering the Molecular Signals of PINK1/Parkin Mitophagy,” Trends in Cell Biology 26, no. 10 (2016): 733–744, 10.1016/j.tcb.2016.05.008. 27291334

[14] D. Xiao , W. Chang , X. Ao , et al., “Parkin Inhibits Iron Overload‐Induced Cardiomyocyte Ferroptosis by Ubiquitinating ACSL4 and Modulating PUFA‐Phospholipids Metabolism,” Acta Pharmaceutica Sinica B 15, no. 3 (2025): 1589–1607, 10.1016/j.apsb.2024.12.027. PMC12069115 40370554

[15] M. Zhang , H. Liu , R. Yin , et al., “PRMT5‐Mediated Arginine Methylation of ACSL4 Attenuates Its Stability and Suppresses Ferroptosis in Renal Cancer,” Research 8 (2025): 0789. 10.34133/research.0789 PMC12314280 40756764

[16] Q. Gao , J. Liu , M. Wang , X. Liu , Y. Jiang , and J. Su , “Biomaterials Regulates BMSCs Differentiation via Mechanical Microenvironment,” Biomaterials Advances 157 (2024): 213738, 10.1016/j.bioadv.2023.213738. 38154401

[17] L. Pinzi and G. Rastelli , “Molecular Docking: Shifting Paradigms in Drug Discovery,” International Journal of Molecular Sciences 20, no. 18 (2019): 4331, 10.3390/ijms20184331. PMC6769923 31487867

[18] L. Gui , Q. Ye , L. Yu , et al., “Bone‐Targeting Peptide and RNF146 Modified Apoptotic Extracellular Vesicles Alleviate Osteoporosis,” International Journal of Nanomedicine 19 (2024): 471–488, 10.2147/IJN.S433511. PMC10800117 38250192

[19] C. Jin , K. Tan , Z. Yao , et al., “A Novel Anti‐Osteoporosis Mechanism of VK2: Interfering With Ferroptosis via AMPK/SIRT1 Pathway in Type 2 Diabetic Osteoporosis,” Journal of Agricultural and Food Chemistry 71, no. 6 (2023): 2745–2761, 10.1021/acs.jafc.2c05632. 36719855

[20] J. Lam , P. Katti , M. Biete , et al., “A Universal Approach to Analyzing Transmission Electron Microscopy With ImageJ,” Cells 10, no. 9 (2021): 2177. 10.3390/cells10092177 PMC8465115 34571826

[21] M. Merkel , B. Goebel , M. Boll , et al., “Mitochondrial Reactive Oxygen Species Formation Determines ACSL4/LPCAT2‐Mediated Ferroptosis,” Antioxidants 12, no. 8 (2023): 1590, 10.3390/antiox12081590. PMC10451816 37627584

[22] L. Schoenmaker , D. Witzigmann , J. A. Kulkarni , et al., “mRNA‐Lipid Nanoparticle COVID‐19 Vaccines: Structure and Stability,” International Journal of Pharmaceutics 601 (2021): 120586, 10.1016/j.ijpharm.2021.120586. PMC8032477 33839230

[23] L. Guo , X. M. Zhang , Y. B. Zhang , X. Huang , and M. H. Chi , “Association of 4‐hydroxynonenal With Classical Adipokines and Insulin Resistance in a Chinese Non‐Diabetic Obese Population,” Nutrición Hospitalaria 34, no. 2 (2017): 363–368, 10.20960/nh.212. 28421791

[24] L. Li , S. Zhao , Z. Leng , et al., “Pathological Mechanisms and Related Markers of Steroid‐Induced Osteonecrosis of the Femoral Head,” Annals of Medicine 56, no. 1 (2024): 2416070, 10.1080/07853890.2024.2416070. PMC11559024 39529511

[25] M. B. Humphrey , L. Russell , M. I. Danila , et al., “2022 American College of Rheumatology Guideline for the Prevention and Treatment of Glucocorticoid‐Induced Osteoporosis,” Arthritis & Rheumatology 75, no. 12 (2023): 2088–2102, 10.1002/art.42646. 37845798

[26] G. Lu , H.‐X. Li , Z.‐W. Song , et al., “Combination of Bone Marrow Mesenchymal Stem Cells and Moxibustion Restores Cyclophosphamide‐Induced Premature Ovarian Insufficiency by Improving Mitochondrial Function and Regulating Mitophagy,” Stem Cell Research & Therapy 15, no. 1 (2024): 102, 10.1186/s13287-024-03709-0. PMC11003045 38589967

[27] Y. Liu , Z. Zhang , Y. Fang , C. Liu , and H. Zhang , “Ferroptosis in Osteoarthritis: Current Understanding,” Journal of Inflammation Research 17 (2024): 8471–8486, 10.2147/JIR.S493001. PMC11552513 39529997

[28] G. Wang , Z. Li , W. Han , et al., “Itaconate Promotes Mitophagy to Inhibit Neuronal Ferroptosis After Subarachnoid Hemorrhage,” Apoptosis 30, no. 3‐4 (2025): 991–1004, 10.1007/s10495-025-02077-1. 39924585

[29] Q. Xue , R. Kang , D. J. Klionsky , D. Tang , J. Liu , and X. Chen , “Copper Metabolism in Cell Death and Autophagy,” Autophagy 19, no. 8 (2023): 2175–2195, 10.1080/15548627.2023.2200554. PMC10351475 37055935

[30] D. Wu , C. B. Spencer , L. Ortoga , H. Zhang , and C. Miao , “Histone Lactylation‐Regulated METTL3 Promotes Ferroptosis via m6A‐Modification on ACSL4 in Sepsis‐Associated Lung Injury,” Redox Biology 74 (2024): 103194, 10.1016/j.redox.2024.103194. PMC11219935 38852200

[31] K. Ding , C. Liu , L. Li , et al., “Acyl‐CoA Synthase ACSL4: An Essential Target in Ferroptosis and Fatty Acid Metabolism,” Chinese Medical Journal 136, no. 21 (2023): 2521–2537, 10.1097/CM9.0000000000002533. PMC10617883 37442770

[32] Y. Zong , Y. Lin , T. Wei , and Q. Cheng , “Lipid Nanoparticle (LNP) Enables mRNA Delivery for Cancer Therapy,” Advanced Materials 35, no. 51 (2023): 2303261, 10.1002/adma.202303261. 37196221

