# Supplementary material for: Ubiquitination of ACSL4 by Parkin Suppresses Ferroptosis and Rescues Glucocorticoid‐Induced Bone Loss
Source: Adv Sci (Weinh). 2026 Jul 13;13(56):e76586. doi: 10.1002/advs.76586 (PMC13359397; doi:10.1002/advs.76586)
Supplement: Supplementary file 1 [file ADVS-13-e76586-s002.docx]

**Supporting Information for**

**Original article**

**Ubiquitination of ACSL4 by Parkin suppresses ferroptosis and rescues glucocorticoid-induced bone loss**

Supporting Figures S1 to S10

Supporting Tables S1 to S2

**Supplementary Figure S1. Single‑cell transcriptomic analysis reveals ferroptosis activation and apoptosis in GCs‑treated bone microenvironment.**

(A) Addmodule score of single‑cell transcriptomes from control and GCs‑treated mouse femurs, colored by ferroptosis module score. (B) Feature plots showing the expression of ferroptosis‑related genes NCOA4, FTL1, and SLC7A11 in control and GCs groups. (C) Genes associated with ferroptosis and lipid metabolism were significantly altered in the mesenchymal lineage. (D–E) Flow cytometry analysis of BMSCs treated with increasing concentrations of DEX (0–200 µmol/L) using Annexin V‑FITC/PI staining. Data are presented as the mean ± SD from 3 independent experiments. Statistical significance is denoted as ^*^*P* < 0.05, ^**^*P* < 0.01, ^***^*P* < 0.001; ns indicates no significant difference.

**Supplementary Figure S2. DEX impairs osteogenic differentiation and promotes adipogenic differentiation of BMSCs.**

(A–B) ALP staining and quantification after 7 days of osteogenic induction. (C–D) ARS staining and quantification after 14–21 days of osteogenic induction. (E–F) Western blot analysis of osteogenic marker proteins (COL1, RUNX2, OCN) in control and DEX-treated BMSCs. (G–H) Oil Red O staining and quantification after 10–14 days of adipogenic induction. (I–J) Western blot analysis of adipogenic marker proteins (Perilipin, PPARγ) in control and DEX‑treated BMSCs. Data are presented as the mean ± SD from 3 independent experiments. Statistical significance is denoted as ^*^*P* < 0.05, ^**^*P* < 0.01, ^***^*P* < 0.001; ns indicates no significant difference.

**Supplementary Figure S3. Micro‑CT quantification of trabecular bone parameters.**

The *in vivo* groups consisted of the Control, GIOP, and GIOP+Fer‑1 groups. (A–D) Quantitative analysis of BV/TV, Tb.Th, Tb.N, and Tb.Sp among the different groups. (E) Quantitative analysis of the percentage of fatty vacuolation in the bone marrow by H&E staining among the different groups. (F) Quantitative analysis of ACSL4 fluorescence intensity in the distal femur among the different groups. Data are presented as the mean ± SD from 5 independent experiments. Statistical significance is denoted as ^*^*P* < 0.05, ^**^*P* < 0.01, ^***^*P* < 0.001; ns indicates no significant difference.

**Supplementary Figure S4. Validation of Parkin knockdown, Parkin overexpression, and ACSL4 overexpression in BMSCs.**

(A–B) Western blot analysis and quantification of Parkin protein levels in BMSCs transfected with sh‑NC (negative control) or sh‑Parkin. (C–D) Western blot analysis and quantification of Parkin protein levels in BMSCs transfected with OE‑NC (empty vector control) or OE‑Parkin. (E–F) Western blot analysis and quantification of ACSL4 protein levels in BMSCs transfected with OE‑NC or OE‑ACSL4. Data are presented as the mean ± SD from 3 independent experiments. Statistical significance is denoted as ^*^*P* < 0.05, ^**^*P* < 0.01, ^***^*P* < 0.001; ns indicates no significant difference.

**Supplementary Figure S5. Parkin modulates osteogenic and adipogenic protein expression in DEX‑treated BMSCs.**

To verify the role of Parkin in osteogenic and adipogenic differentiation of BMSCs, we set up the following four groups: DEX+sh-NC, DEX+sh-Parkin, DEX+OE-NC, DEX+OE-Parkin. (A–B) Western blot analysis and quantification of osteogenic marker proteins (COL1, RUNX2, OCN) in BMSCs treated with DEX (200 µmol/L) following Parkin knockdown (sh‑Parkin) or overexpression (OE‑Parkin). (C–D) Western blot analysis and quantification of adipogenic marker proteins (Perilipin, PPARγ) under the same conditions. Data are presented as the mean ± SD from 3 independent experiments. Statistical significance is denoted as ^*^*P* < 0.05, ^**^*P* < 0.01, ^***^*P* < 0.001; ns indicates no significant difference.

**Supplementary Figure S6. Validation of Parkin wild‑type and E3 ligase‑defective mutants.**

(A–D) Western blot analysis the effects of DEX on ACSL4 ubiquitinationwith or without MG132. (E) The MDA levels in Parkin wild‑type (pcDNA3.1‑Parkin) and the indicated mutants (T415N, P437L, G430D) wtih DEX treatment. Data are presented as the mean ± SD from 3 independent experiments. Statistical significance is denoted as ^*^*P* < 0.05, ^**^*P* < 0.01, ^***^*P* < 0.001; ns indicates no significant difference.

**Supplementary Figure S7. Validation of Parkin‑ACSL4 interaction and Parkin‑mediated ACSL4 ubiquitination in BMSCs.**

(A–B) Co‑IP in BMSCs validating endogenous interaction between Parkin and ACSL4. (C–D) DEX treatment weakens the Parkin‑ACSL4 interaction. (E–F) BMSCs were transfected with HA-ubiquitin along with Parkin overexpression plasmid or control PcDNA3.1, respectively. Ubiquitination of ACSL4 was analyzed by IP in BMSCs. (G–J) Western blot analysis the effects of DEX on ACSL4 ubiquitinationwith or without MG132 in BMSCs. Data are presented as the mean ± SD from 3 independent experiments.

**Supplementary Figure S8. *In vivo* biodistribution and hydroxyapatite binding of DSS6-modified LNPs.**

(A–C) Quantitative analysis of ex vivo fluorescence intensity of ICG-labeled LNPs in major organs (heart, liver, spleen, lung, kidney, and hindlimb) at 6 h, 24 h, and 48 h post-injection. (D) The fluorescence intensity of LNPs (Vector-LNP or DSS6-LNP, both labeled with ICG). Data are presented as the mean ± SD from 3 independent experiments. Statistical significance is denoted as ^*^*P* < 0.05, ^**^*P* < 0.01, ^***^*P* < 0.001; ns indicates no significant difference.

**Supplementary Figure S9. Parkin‑LNP@DSS6 protects against SONFH.**

(A–B) Representative micro‑CT images of the femoral head in SONFH model mice with different treatments. (C–F) Quantitative analysis of BV/TV, Tb.Th, Tb.N, and Tb.Sp among the different groups. (G) Representative H&E staining images of the femoral head in SONFH model mice with different treatments. Data are presented as the mean ± SD from 5 independent experiments. Statistical significance is denoted as ^*^*P* < 0.05, ^**^*P* < 0.01, ^***^*P* < 0.001; ns indicates no significant difference.

**Supplementary Figure S10. Parkin‑LNP@DSS6 treatment has no toxic effects on major organs in mice.**

(A) H&E staining of heart, lung, liver, spleen, and kidney in control and Parkin-LNP@DSS6 group. (B–C) Serum ALT/AST levels in control and Parkin-LNP@DSS6 group. (D) Serum BUN levels in control and Parkin-LNP@DSS6 group. Data are presented as the mean ± SD from 3 independent experiments. Statistical significance is denoted as ^*^*P* < 0.05, ^**^*P* < 0.01, ^***^*P* < 0.001; ns indicates no significant difference.

**Supplementary Table S1. The Primary Antibodies**

| Antibody | Isotype | Manufacturer | Cat.No | Dilution |
| --- | --- | --- | --- | --- |
| TOM20 | Mouse | abcam | ab186735 | 1:1000 |
| DRP1 | Mouse | abcam | ab184247 | 1:1000 |
| FTH1 | Rabbit | Cell Signaling Technology | #3998 | 1:1000 |
| MFN1 | Rabbit | Cell Signaling Technology | #14739 | 1:1000 |
| PPARγ | Rabbit | Cell Signaling Technology | #2435 | 1:1000 |
| Perilipin | Rabbit | Cell Signaling Technology | #3470 | 1:1000 |
| β-actin | Rabbit | Cell Signaling Technology | #4970 | 1:2000 |
| PTGS2 | Rabbit | Proteintech | 27308-1-AP | 1:1000 |
| ACSL4 | Rabbit | Proteintech | 22401-1-AP | 1:1000 |
| GPX4 | Mouse | Proteintech | 67763-1-Ig | 1:1000 |
| Parkin | Rabbit | Proteintech | 14060-1-AP | 1:1000 |
| OPA1 | Mouse | Proteintech | 66583-1-Ig | 1:1000 |
| MFN2 | Mouse | Proteintech | 67487-1-Ig | 1:500 |
| RUNX2 | Rabbit | Proteintech | 20700-1-AP | 1:1000 |
| OCN | Rabbit | Proteintech | 23418-1-AP | 1:500 |
| COL1A1 | Rabbit | Proteintech | 86093-1-RR | 1:2000 |
| 4-HNE | Mouse | Proteintech | 68538-1-Ig | 1:1000 |
| Ubiquitin | Rabbit | Proteintech | 10201-2-AP | 1:1000 |

**Supplementary Table S2. Primers used for Gene**

| Gene name | Species |  |
| --- | --- | --- |
| PTGS2 - Forward | Mouse | TGAGTACCGCAAACGCTTCT |
| PTGS2- Reverse | Mouse | CAGCCATTTCCTTCTCTCCTGT |
| ACSL4- Forward | Mouse | GGAAAGCAAACTGAAGGCGG |
| ACSL4- Reverse | Mouse | CCCTCAGGGTACTCTGCTCT |
| Parkin- Forward | Mouse | AGCCAGAGGTCCAGCAGTTA |
| Parkin- Reverse | Mouse | GAGGGTTGCTTGTTTGCAGG |
| LPCAT3- Forward | Mouse | CTTCTTGGTAGGGCCCCAAT |
| LPCAT3- Reverse | Mouse | ACCAGAAAGGGCGGTTATCA |
| FASN- Forward | Mouse | AGATGGAAGGCTGGGCTCTA |
| FASN- Reverse | Mouse | CCTCTGAACCACTCACACCC |
| SCD1- Forward | Mouse | CACTTGGGAGGCCTGTACG |
| SCD1- Reverse | Mouse | TGGTCATGTAGTAGAAAATCCCGAA |
| ELOVL6- Forward | Mouse | AGCACCCGAACTAGGTGACA |
| ELOVL6- Reverse | Mouse | CCGCAAGGCGTAGTAAGAGT |
| GAPDH- Forward | Mouse | CAGGAGAGTGTTTCCTCGTCC |
| GAPDH- Reverse | Mouse | GATGGGCTTCCCGTTGATGA |
