# Supplementary material for: Ubiquitination of ACSL4 by Parkin Suppresses Ferroptosis and Rescues Glucocorticoid‐Induced Bone Loss
Source: Adv Sci (Weinh). 2026 Jul 13:e76586. Online ahead of print. doi: 10.1002/advs.76586 (PMC13359397; doi:10.1002/advs.76586)
Supplement: Supplementary file 2 — Supporting File 2: advs76586‐sup‐0002‐Data.zip. [file ADVS-9999-e76586-s001.zip › Supplemental Data 2.pdf]

Fig. 1C

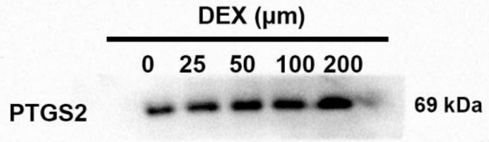

Fig. 1C

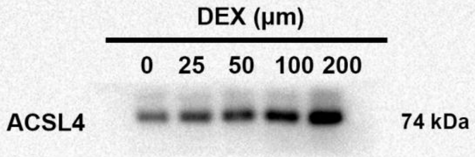

Fig. 1C

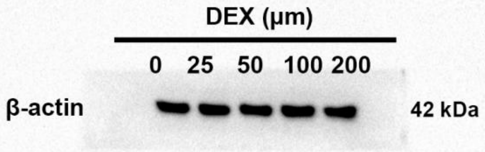

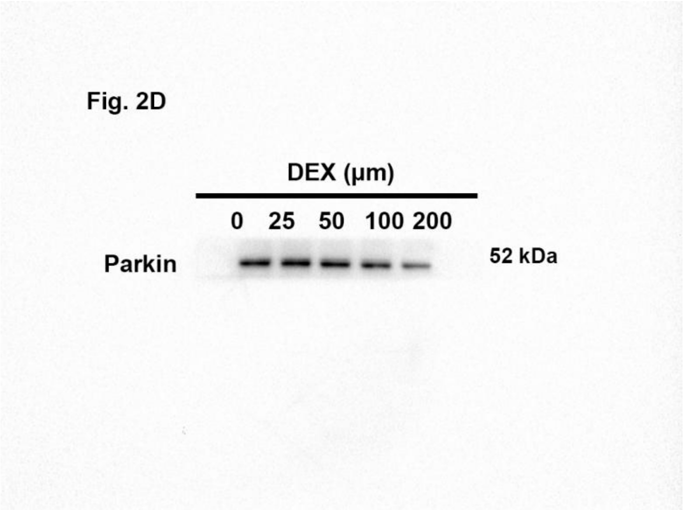
Fig. 2D
DEX (µm)
0 25 50 100 200
Parkin
52 kDa

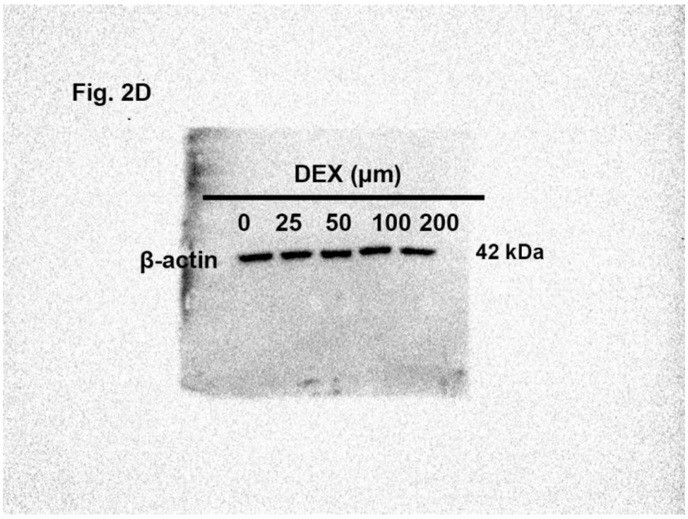
Fig. 2D
DEX (µm)
0 25 50 100 200
β-actin
42 kDa

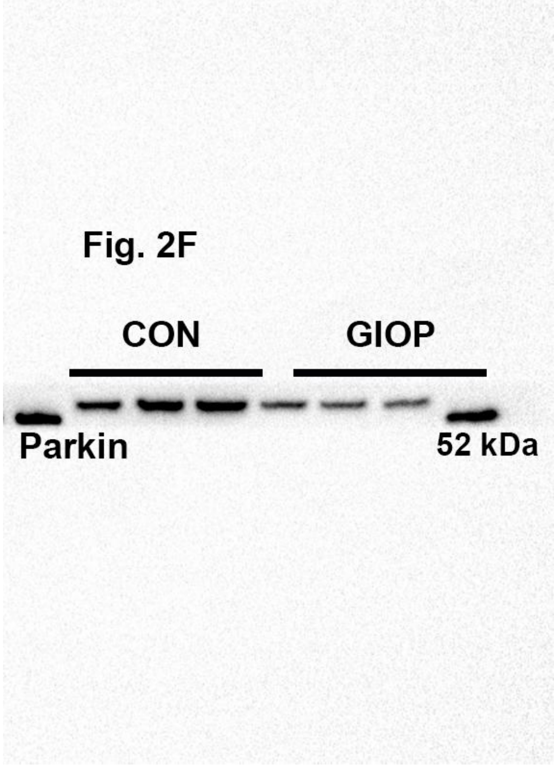
Fig. 2F
CON
GIOP
Parkin
52 kDa

Fig. 2F

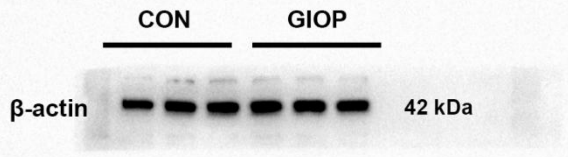

Fig. 2S

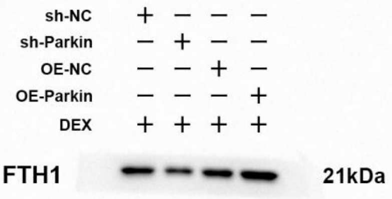

Fig. 2S

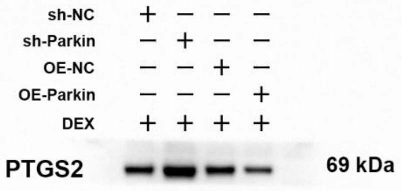

Fig. 2S

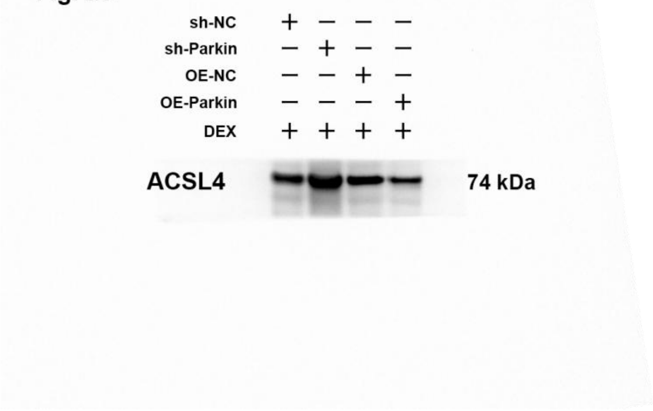

Fig. 2S

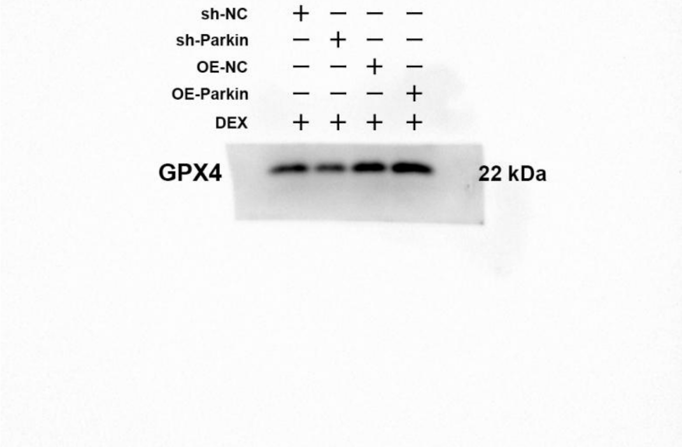

Fig. 2S

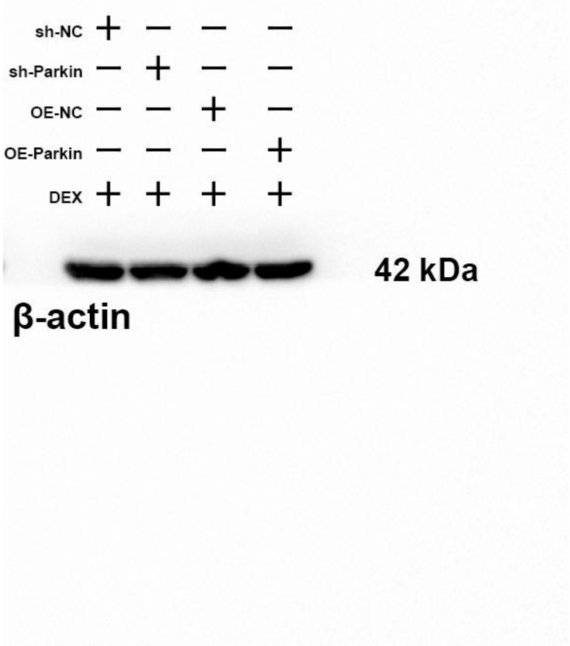

Fig. 3I

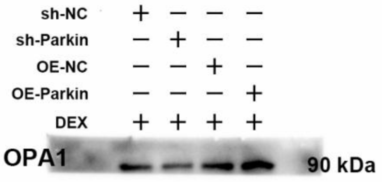

Fig. 3I

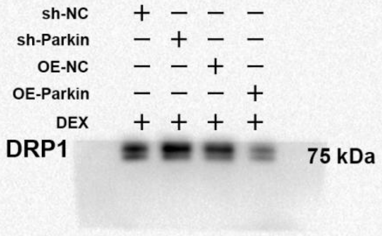

Fig. 3I

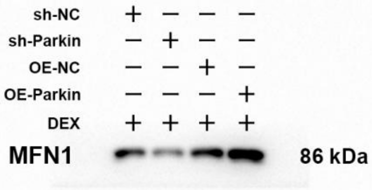

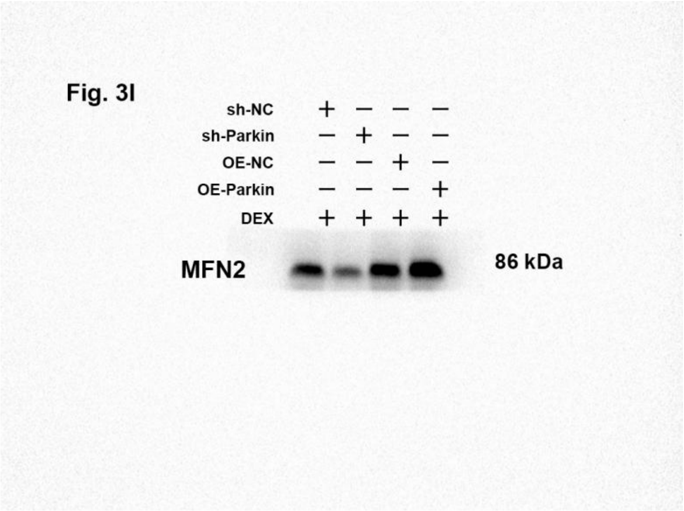
Fig. 3I
sh-NC + − − −
sh-Parkin − + − −
OE-NC − − + −
OE-Parkin − − − +
DEX + + + +
MFN2
86 kDa

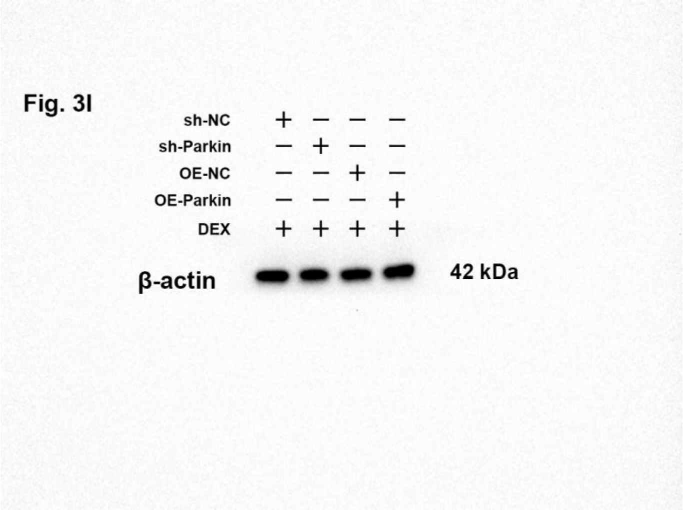
Fig. 3I
sh-NC + − − −
sh-Parkin − + − −
OE-NC − − + −
OE-Parkin − − − +
DEX + + + +
β-actin
42 kDa

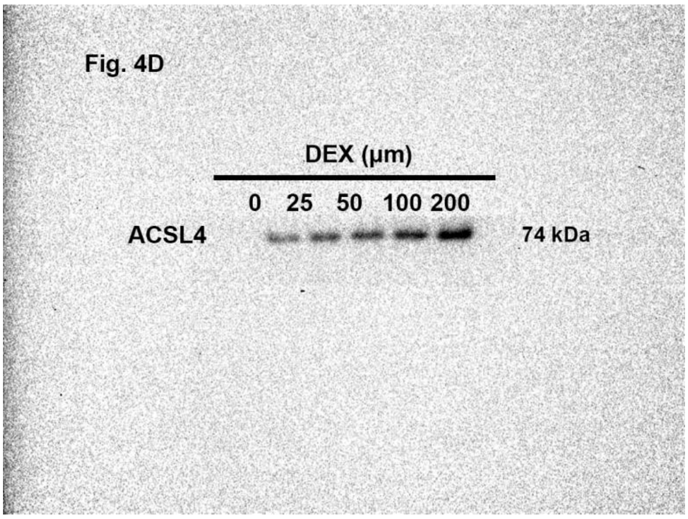
Fig. 4D
DEX (μm)
0 25 50 100 200
ACSL4
74 kDa

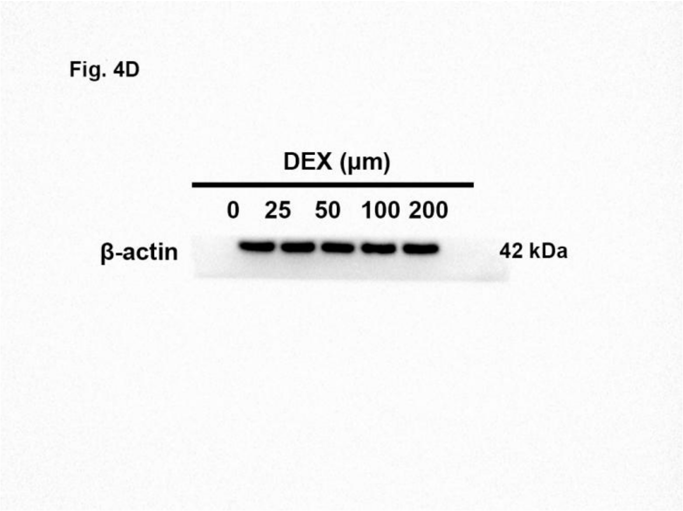
Fig. 4D
DEX (μm)
0 25 50 100 200
β-actin
42 kDa

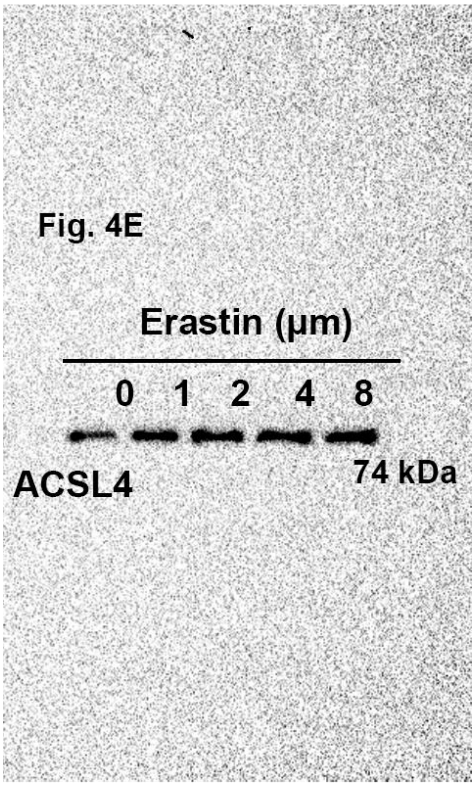
Fig. 4E
Erastin (μm)
0 1 2 4 8
ACSL4
74 kDa

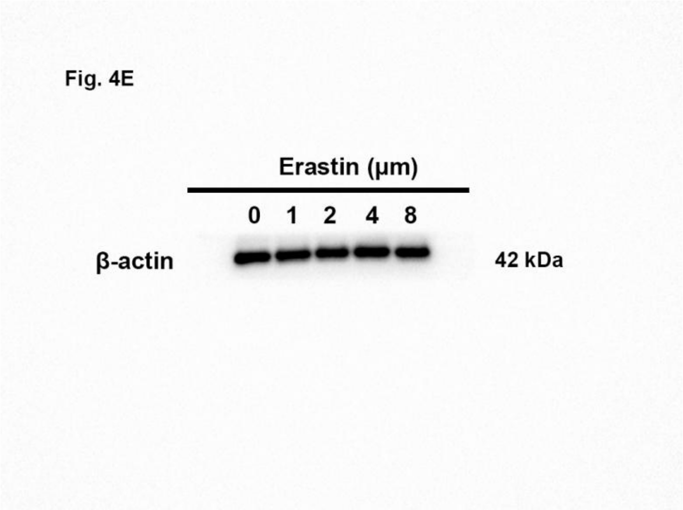
Fig. 4E
Erastin (μm)
0 1 2 4 8
β-actin
42 kDa

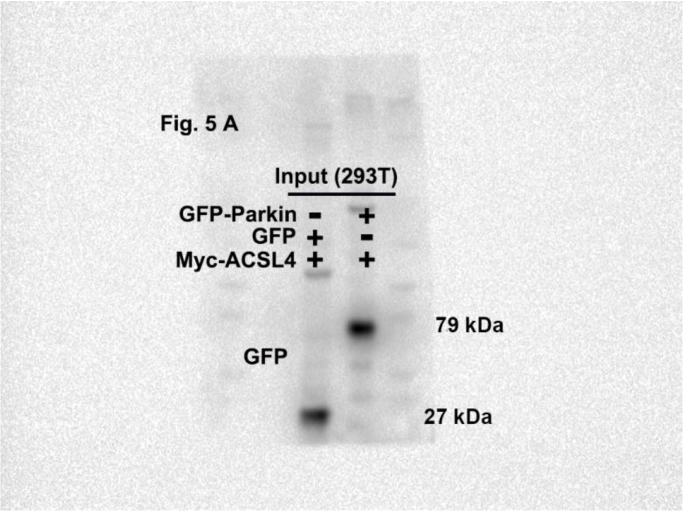
Fig. 5 A
Input (293T)
GFP-Parkin - +
GFP + -
Myc-ACSL4 + +
GFP
79 kDa
27 kDa

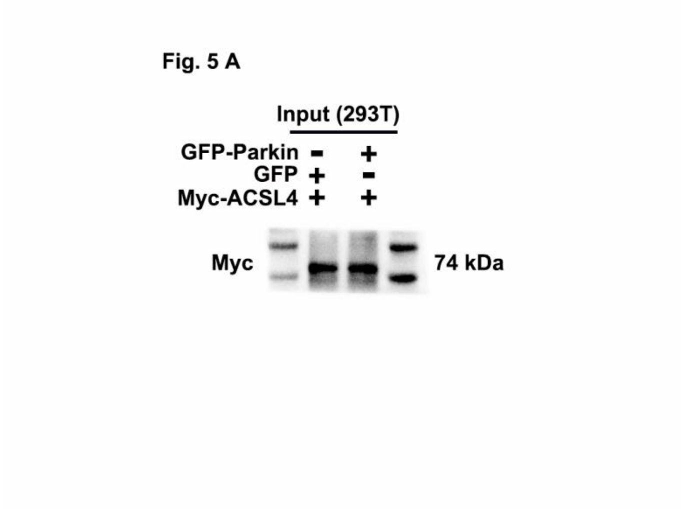
Fig. 5 A
Input (293T)
GFP-Parkin - +
GFP + -
Myc-ACSL4 + +
Myc
74 kDa

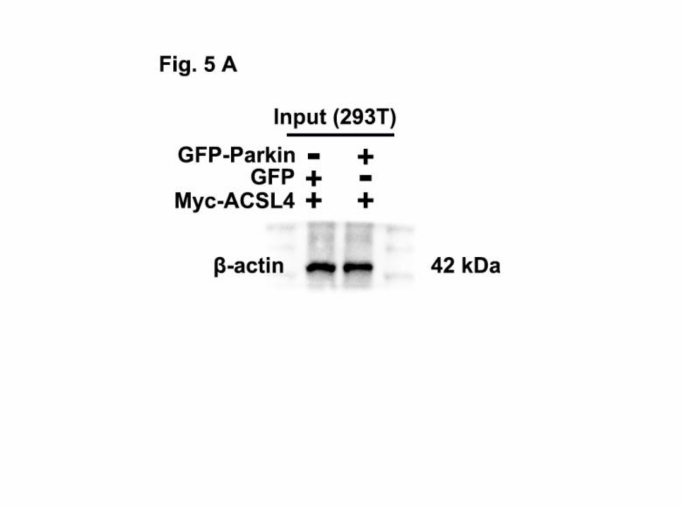
Fig. 5 A
Input (293T)
GFP-Parkin - +
GFP + -
Myc-ACSL4 + +
β-actin
42 kDa

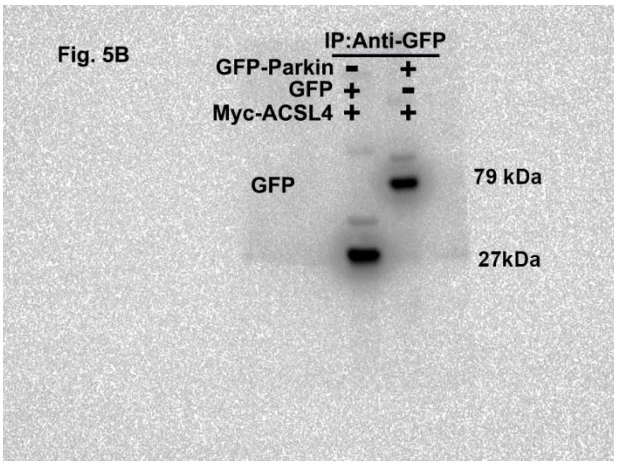
Fig. 5B
IP:Anti-GFP
GFP-Parkin - +
GFP + -
Myc-ACSL4 + +
GFP
79 kDa
27kDa

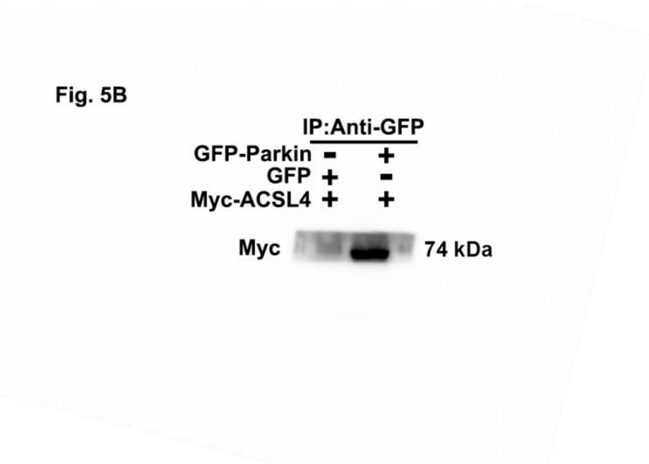
Fig. 5B
IP:Anti-GFP
GFP-Parkin - +
GFP + -
Myc-ACSL4 + +
Myc
74 kDa

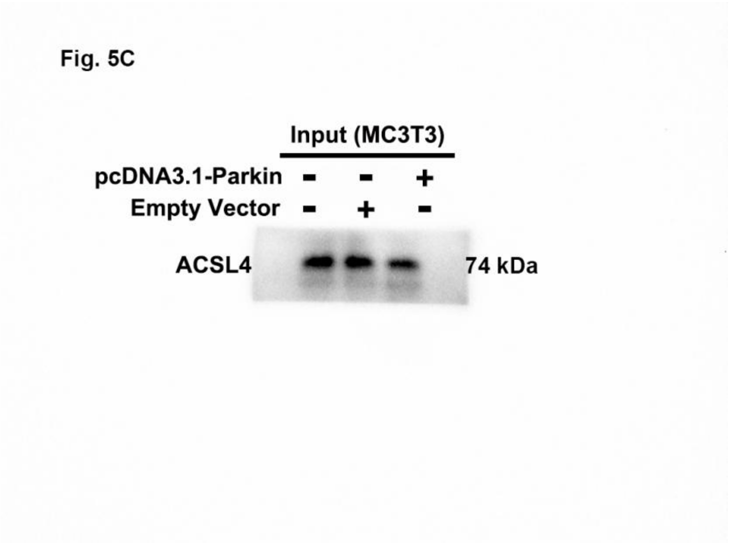
Fig. 5C
Input (MC3T3)
pcDNA3.1-Parkin - - +
Empty Vector - + -
ACSL4
74 kDa

Fig. 5C

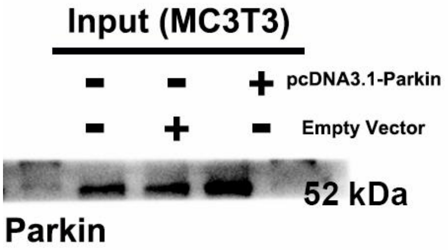

Fig. 5C

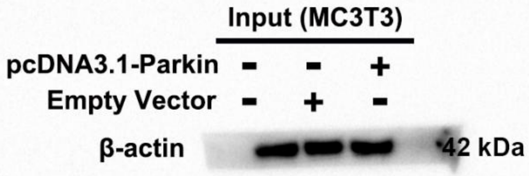

Fig. 5D

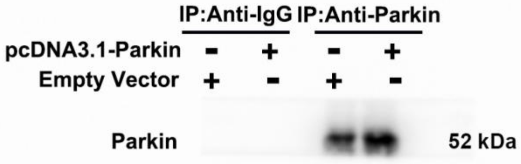

Fig. 5D

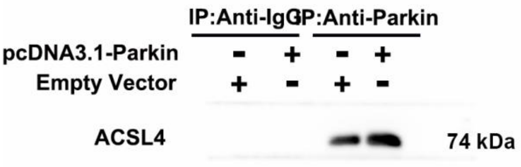
IP:Anti-IgG
IP:Anti-Parkin
pcDNA3.1-Parkin - + - +
Empty Vector + - + -
ACSL4
74 kDa

Fig. 5E

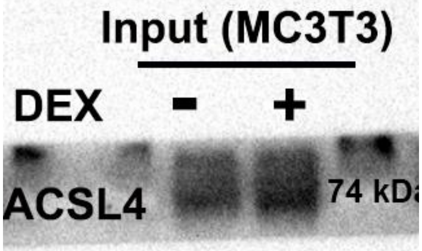
Input (MC3T3)
DEX - +
ACSL4
74 kDa

Fig. 5E

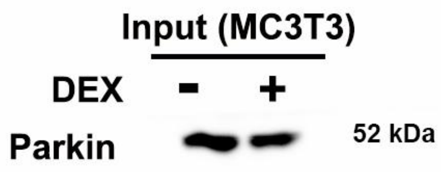
Input (MC3T3)
DEX - +
Parkin
52 kDa

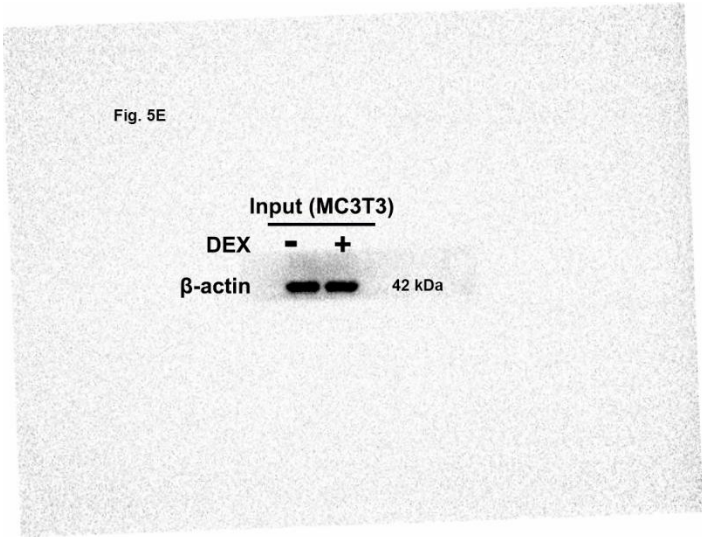
Fig. 5E
Input (MC3T3)
DEX - +
β-actin
42 kDa

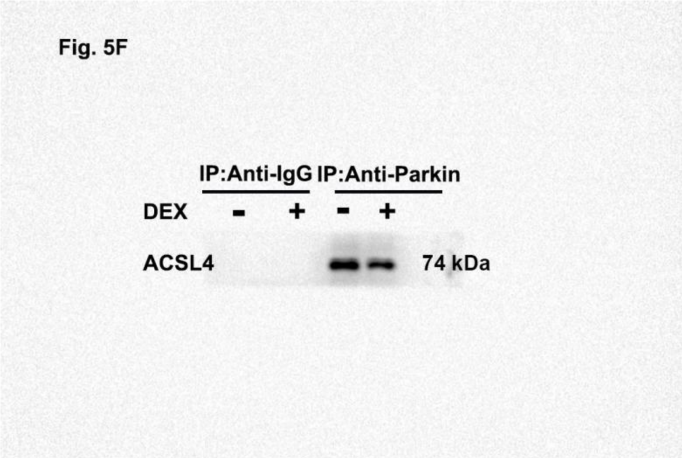
Fig. 5F
IP:Anti-IgG
IP:Anti-Parkin
DEX - + - +
ACSL4
74 kDa

Fig. 5F

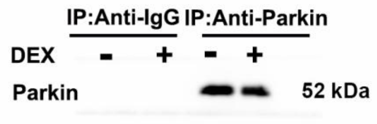

Fig. 5G

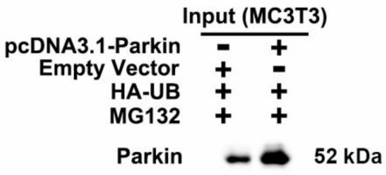

Fig. 5G

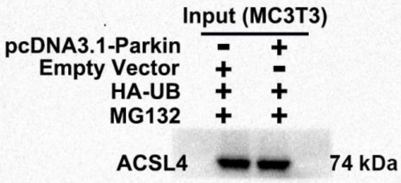

Fig. 5G

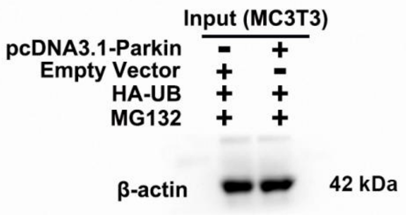

Fig. 5H

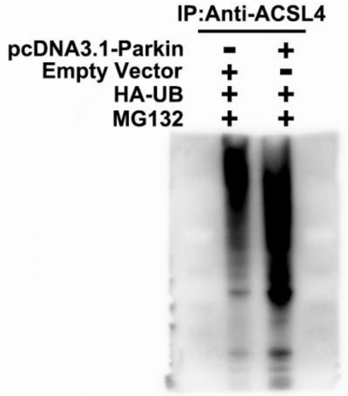

Fig. 5H

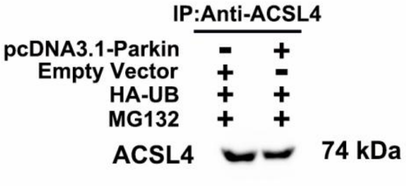

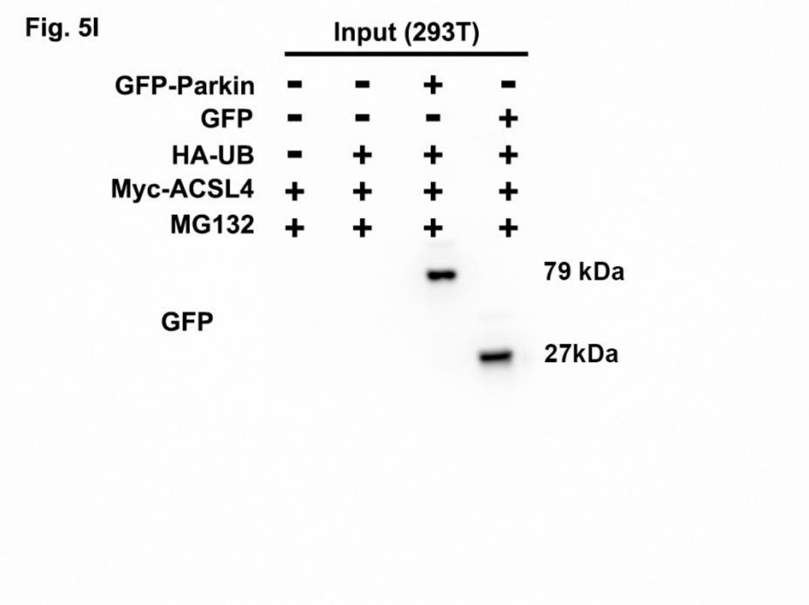

Fig. 5I
Input (293T)
GFP-Parkin - - + -
GFP - - - +
HA-UB - + + +
Myc-ACSL4 + + + +
MG132 + + + +
79 kDa
GFP
27kDa

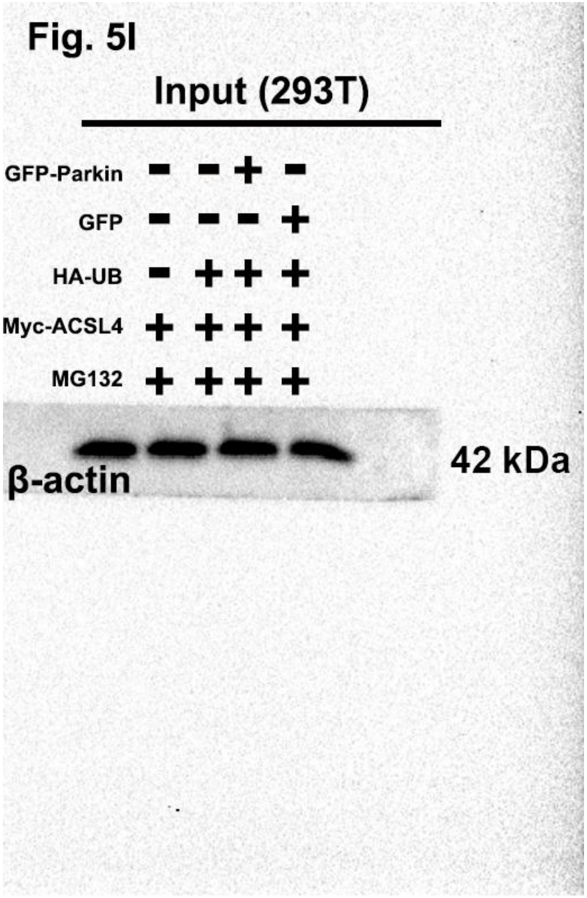

Fig. 5I
Input (293T)
GFP-Parkin - - + -
GFP - - - +
HA-UB - + + +
Myc-ACSL4 + + + +
MG132 + + + +
β-actin
42 kDa

Fig. 5I

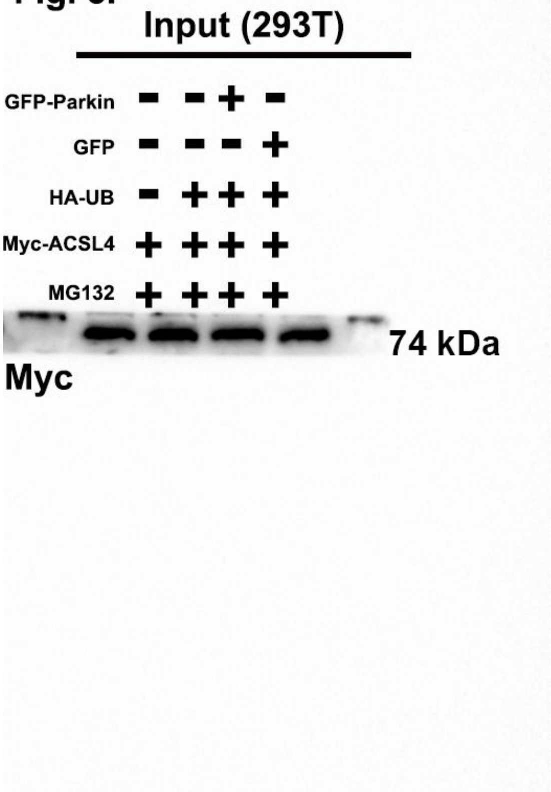
Input (293T)
GFP-Parkin - - + -
GFP - - - +
HA-UB - + + +
Myc-ACSL4 + + + +
MG132 + + + +
74 kDa
Myc

Fig. 5J

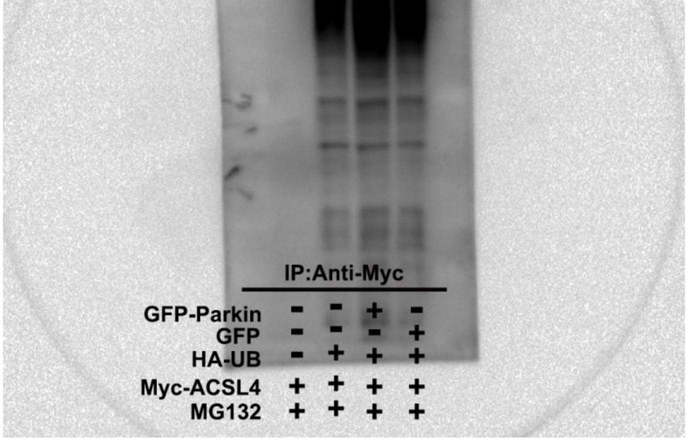
IP:Anti-Myc
GFP-Parkin - - + -
GFP - - - +
HA-UB - + + +
Myc-ACSL4 + + + +
MG132 + + + +

Fig. 5J

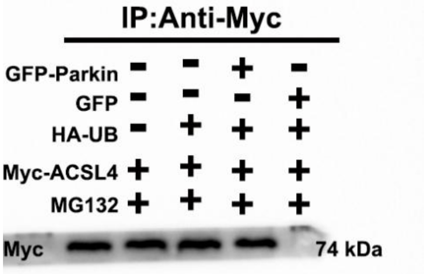
IP:Anti-Myc
GFP-Parkin - - + -
GFP - - - +
HA-UB - + + +
Myc-ACSL4 + + + +
MG132 + + + +
Myc
74 kDa

Fig. 5K

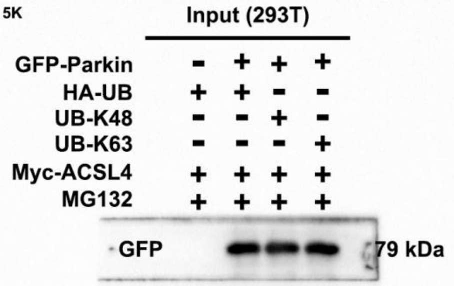
Input (293T)
GFP-Parkin - + + +
HA-UB + + - -
UB-K48 - - + -
UB-K63 - - - +
Myc-ACSL4 + + + +
MG132 + + + +
GFP
79 kDa

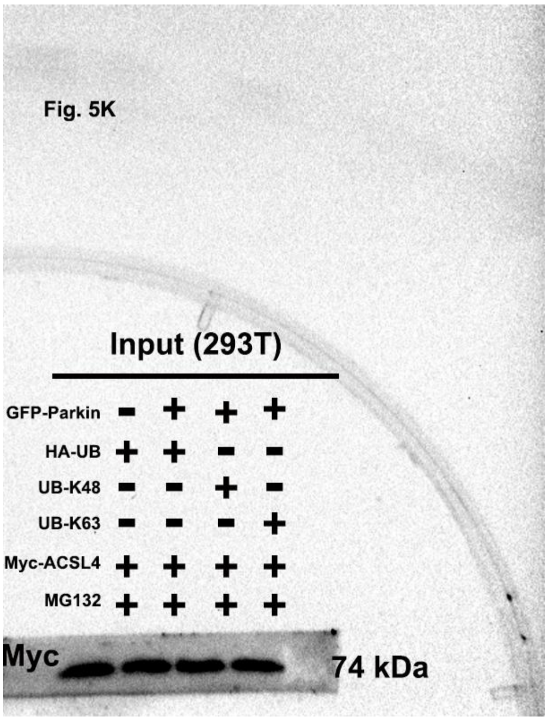
Fig. 5K
Input (293T)
GFP-Parkin - + + +
HA-UB + + - -
UB-K48 - - + -
UB-K63 - - - +
Myc-ACSL4 + + + +
MG132 + + + +
Myc
74 kDa

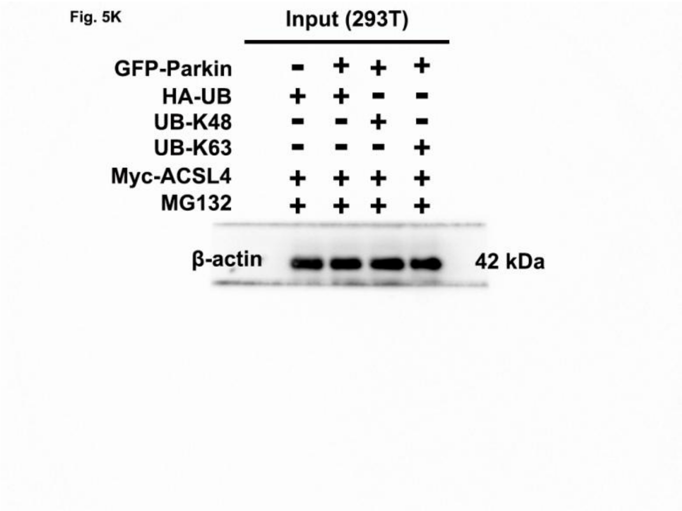
Fig. 5K
Input (293T)
GFP-Parkin - + + +
HA-UB + + - -
UB-K48 - - + -
UB-K63 - - - +
Myc-ACSL4 + + + +
MG132 + + + +
β-actin
42 kDa

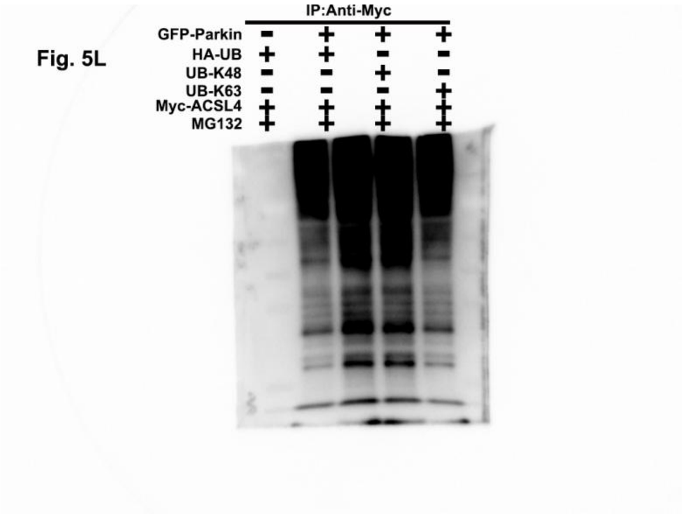
Fig. 5L
IP:Anti-Myc
GFP-Parkin - + + +
HA-UB + + - -
UB-K48 - - + -
UB-K63 - - - +
Myc-ACSL4 + + + +
MG132 + + + +

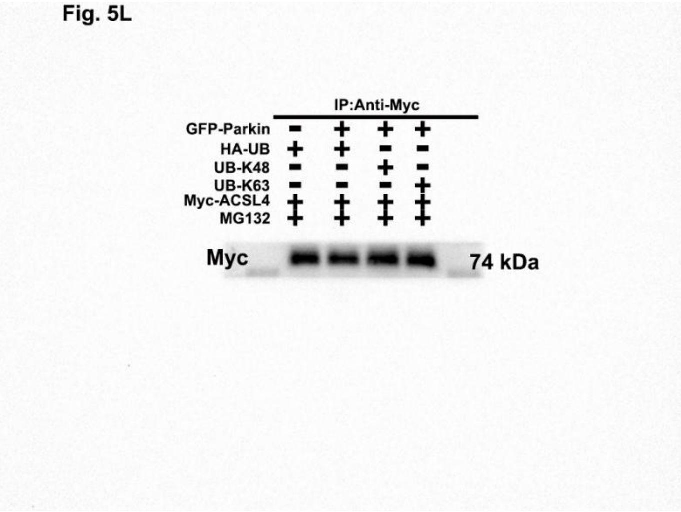

Fig. 5L
IP:Anti-Myc
GFP-Parkin - + + +
HA-UB + + - -
UB-K48 - - + -
UB-K63 - - - +
Myc-ACSL4 + + + +
MG132 + + + +
Myc
74 kDa

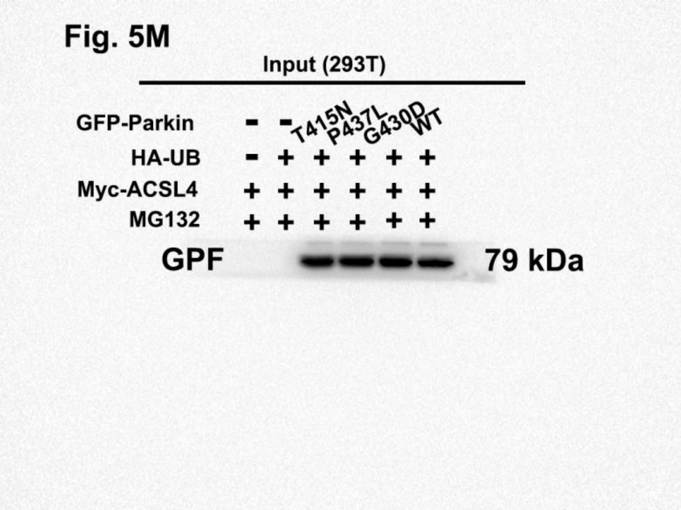

Fig. 5M
Input (293T)
GFP-Parkin - - T415N P437L G430D WT
HA-UB - + + + + +
Myc-ACSL4 + + + + + +
MG132 + + + + + +
GPF
79 kDa

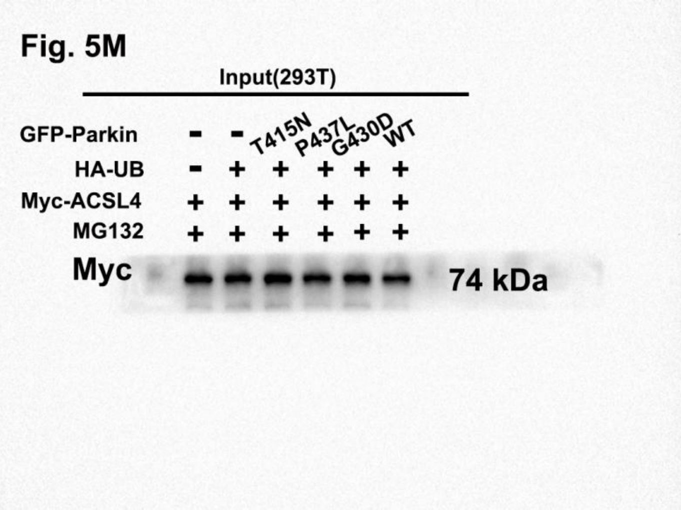

Fig. 5M
Input(293T)
GFP-Parkin - - T415N P437L G430D WT
HA-UB - + + + + +
Myc-ACSL4 + + + + + +
MG132 + + + + + +
Myc
74 kDa

Fig. 5M

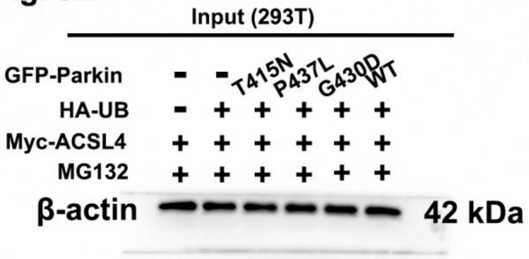

Fig. 5M

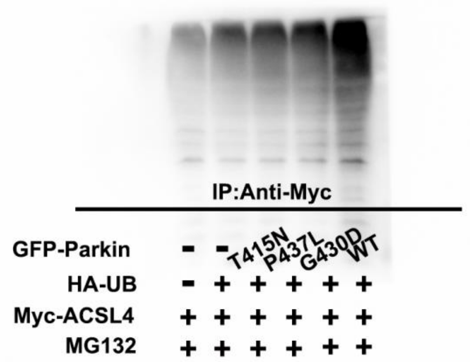

Fig. 5M

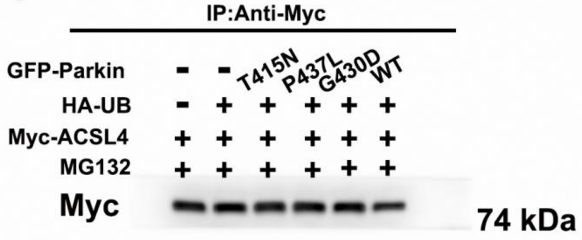

Fig. 6E

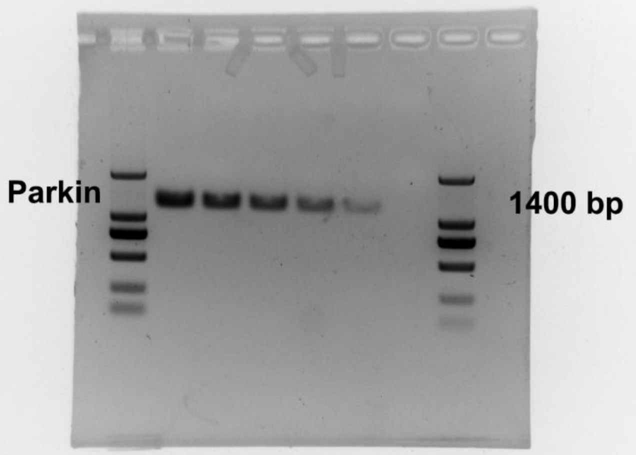

Fig. 6H

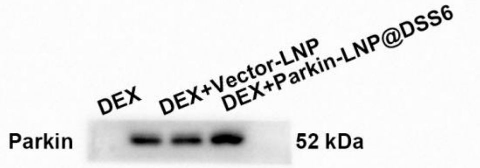

Fig. 6H

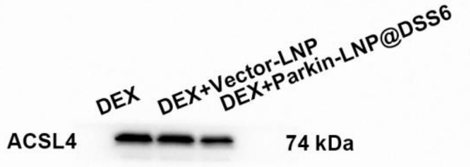

Fig. 6H

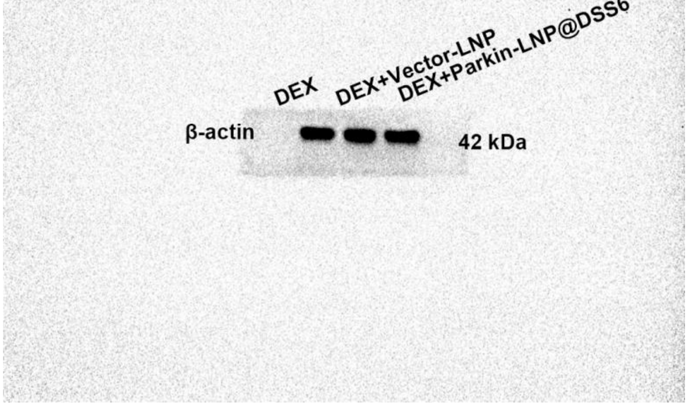

Fig. 6J

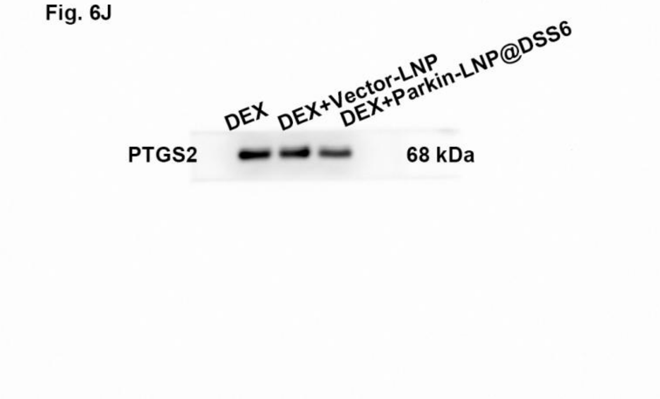

Fig. 6J

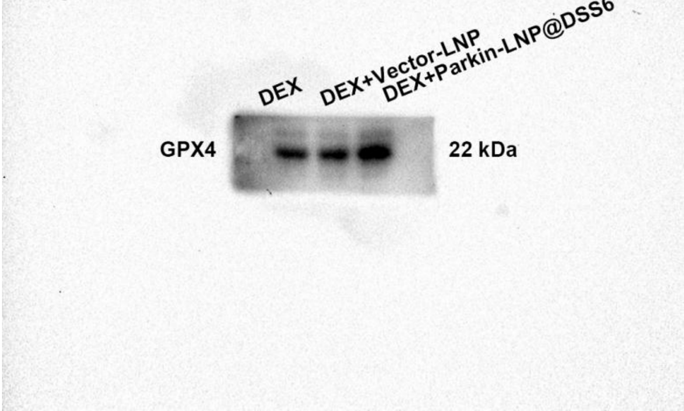

Fig. 6J

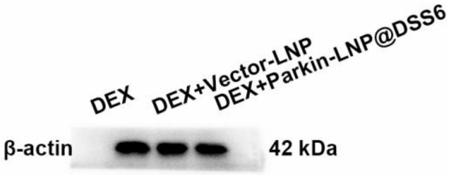

Fig. 6P

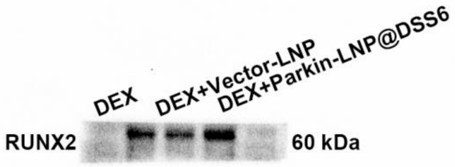

Fig. 6P

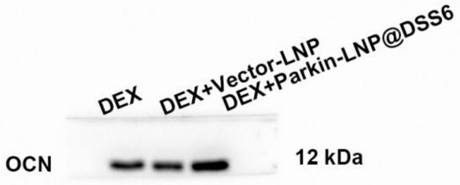

Fig. 6P

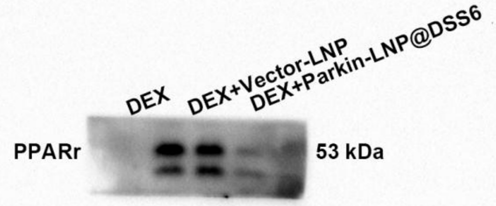

Fig. 6P

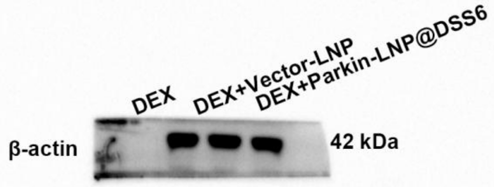

Fig. 6X

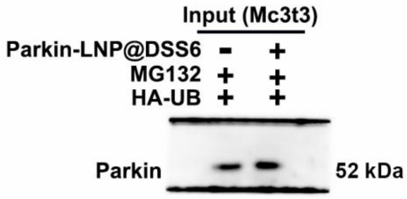

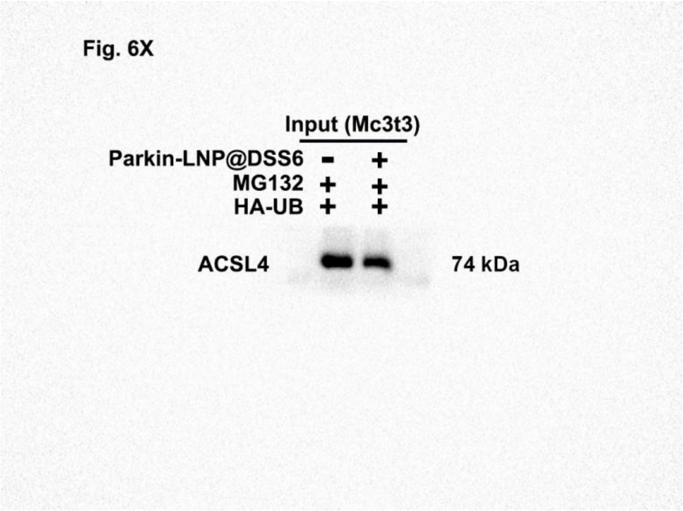
Fig. 6X
Input (Mc3t3)
Parkin-LNP@DSS6 - +
MG132 + +
HA-UB + +
ACSL4
74 kDa

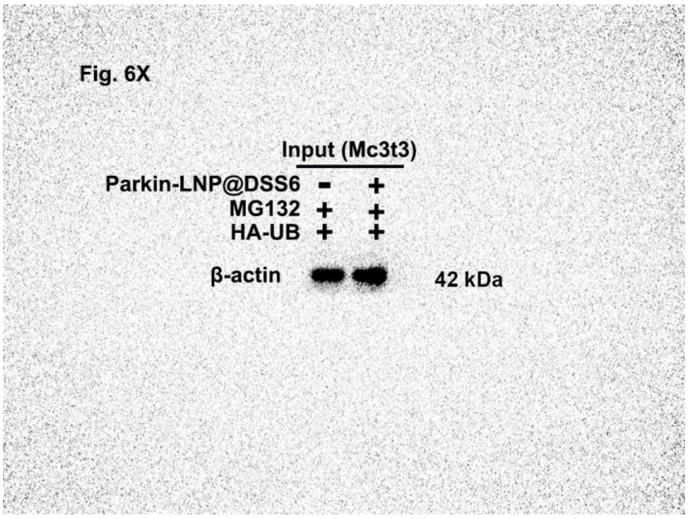
Fig. 6X
Input (Mc3t3)
Parkin-LNP@DSS6 - +
MG132 + +
HA-UB + +
β-actin
42 kDa

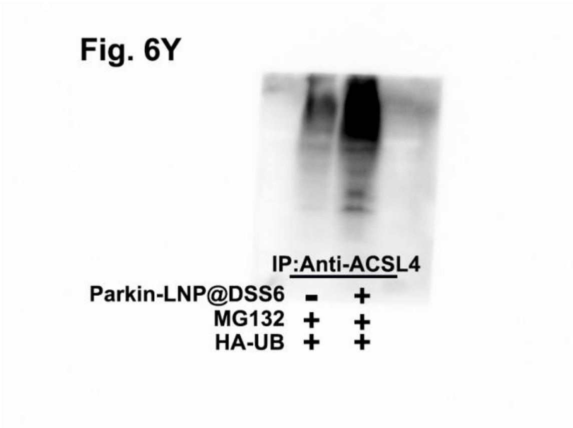
Fig. 6Y
IP:Anti-ACSL4
Parkin-LNP@DSS6 - +
MG132 + +
HA-UB + +

Fig. 6Y

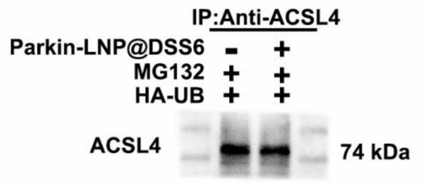

Fig. 8I

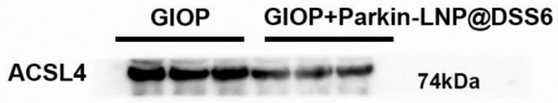

Fig. 8I

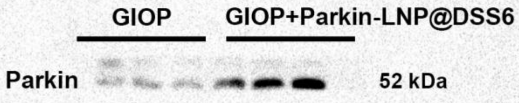

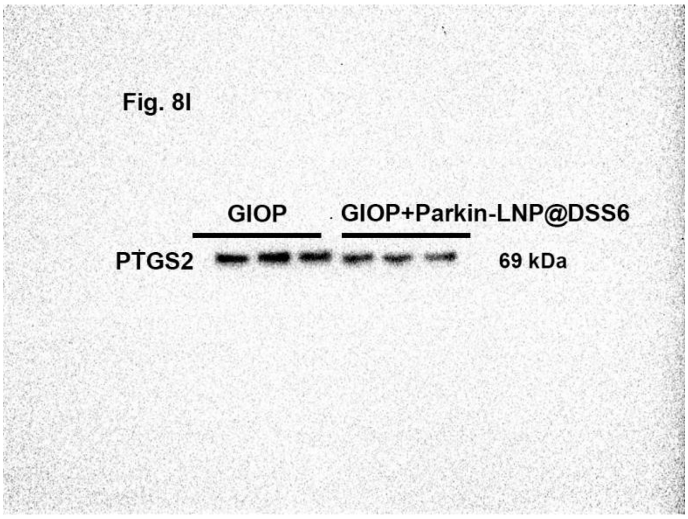
Fig. 8l
GIOP
GIOP+Parkin-LNP@DSS6
PTGS2
69 kDa

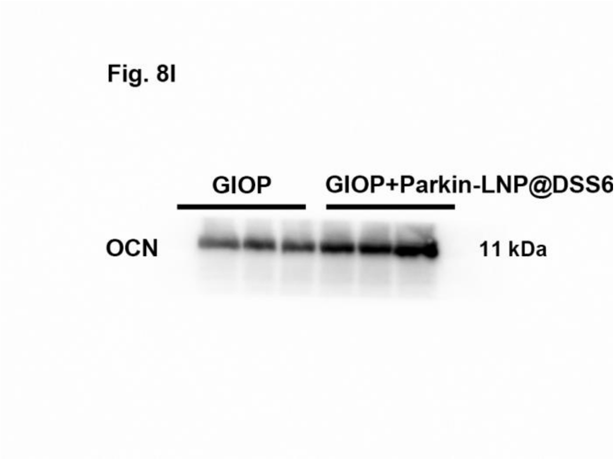
Fig. 8l
GIOP
GIOP+Parkin-LNP@DSS6
OCN
11 kDa

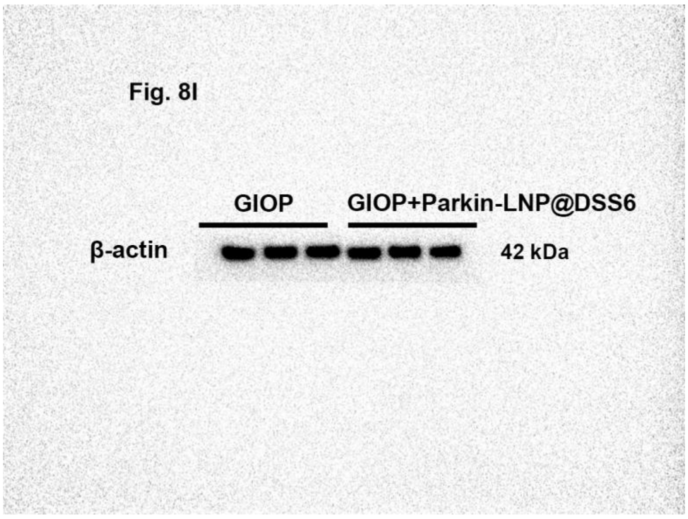
Fig. 8l
GIOP
GIOP+Parkin-LNP@DSS6
β-actin
42 kDa

Fig. S2E

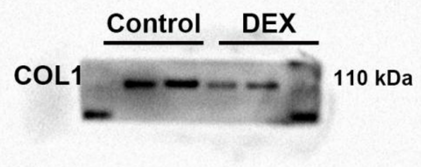

Fig. S2E

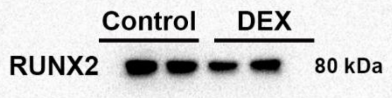

Fig. S2E

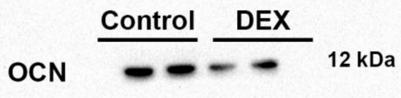

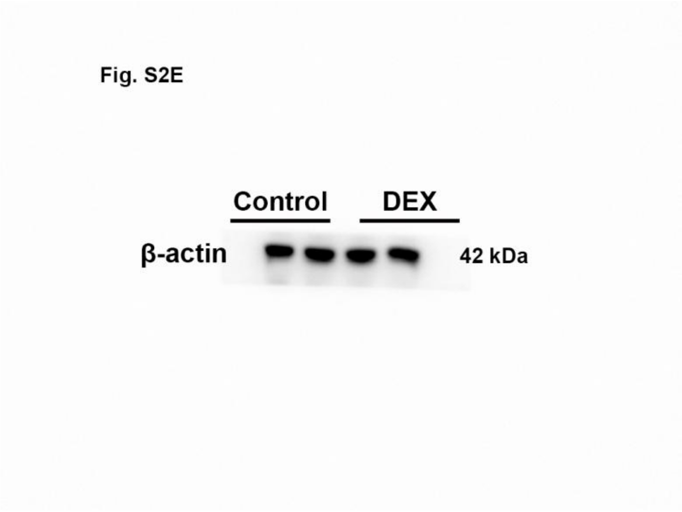
Fig. S2E
Control
DEX
β-actin
42 kDa

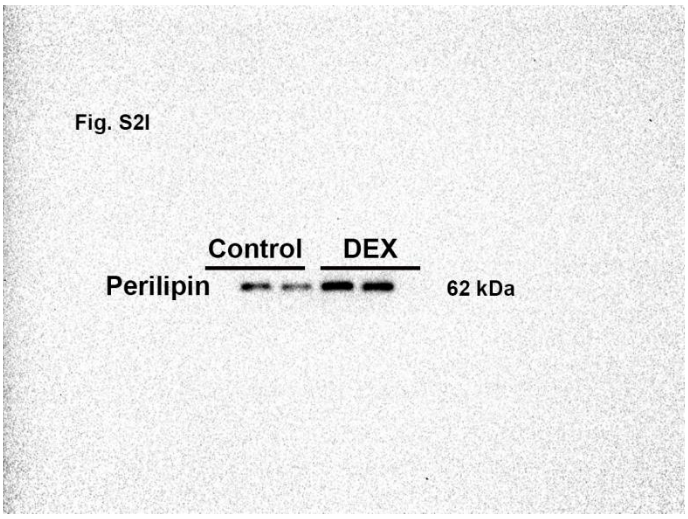
Fig. S2I
Control
DEX
Perilipin
62 kDa

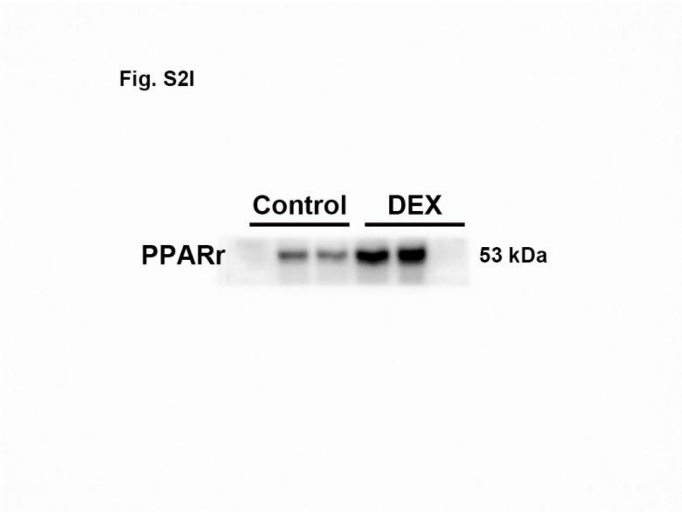
Fig. S2I
Control
DEX
PPARr
53 kDa

Fig. S2I

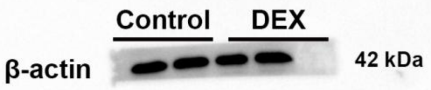

Fig. S4A

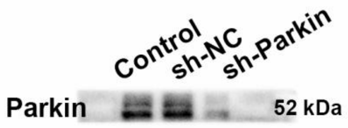

Fig. S4A

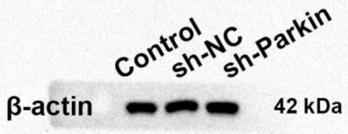

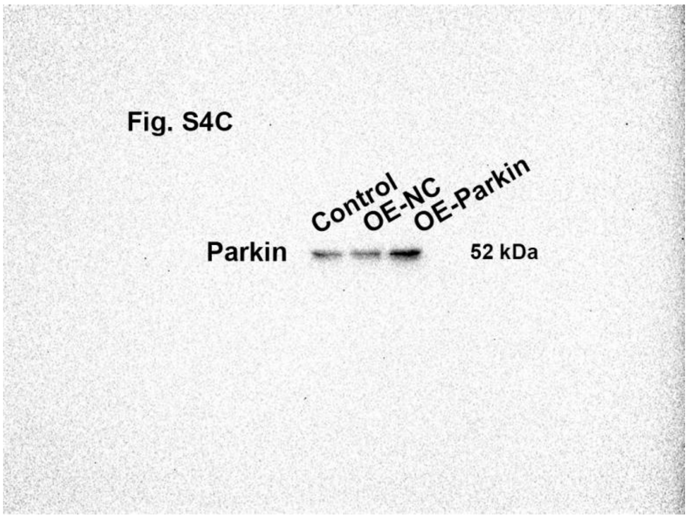
Fig. S4C
Control
OE-NC
OE-Parkin
Parkin
52 kDa

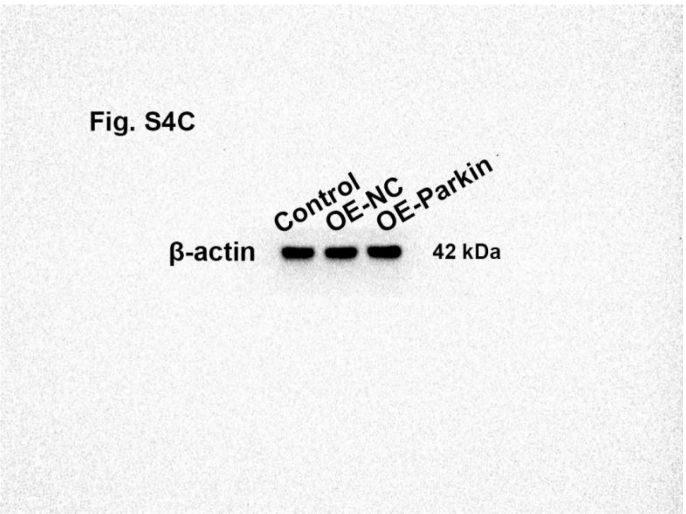
Fig. S4C
Control
OE-NC
OE-Parkin
β-actin
42 kDa

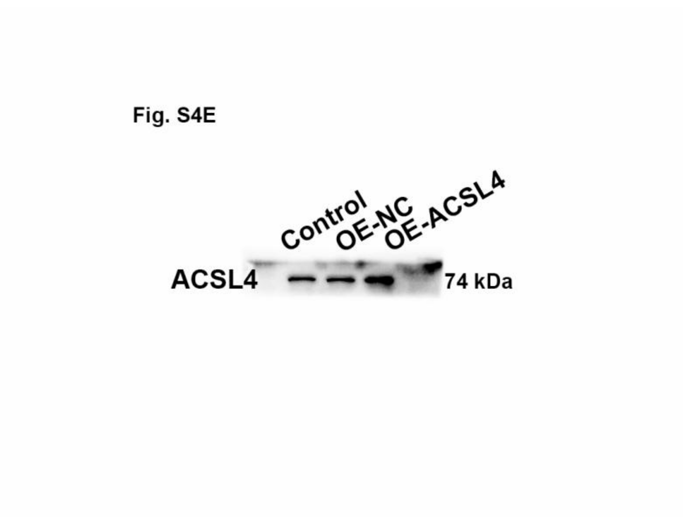
Fig. S4E
Control
OE-NC
OE-ACSL4
ACSL4
74 kDa

Fig. S4E

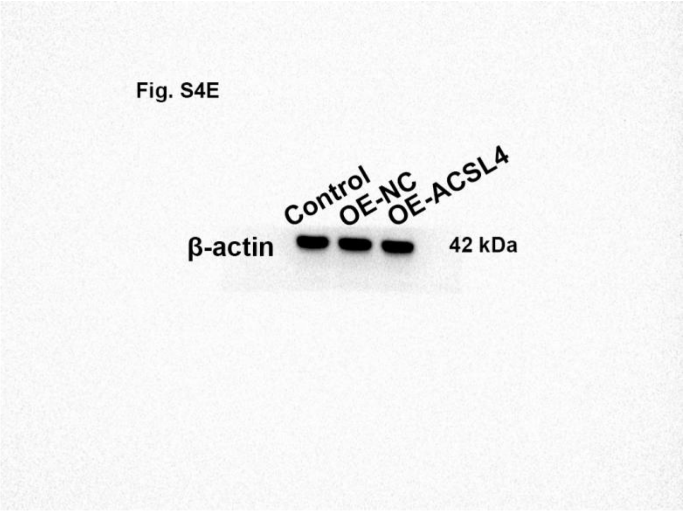

Fig. S5

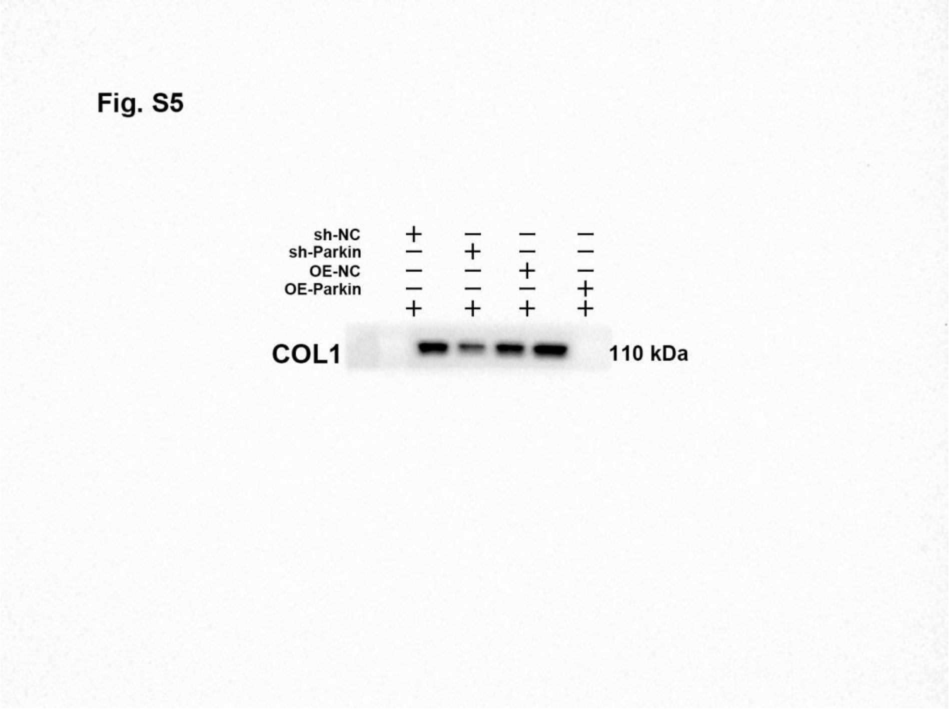

Fig. S5

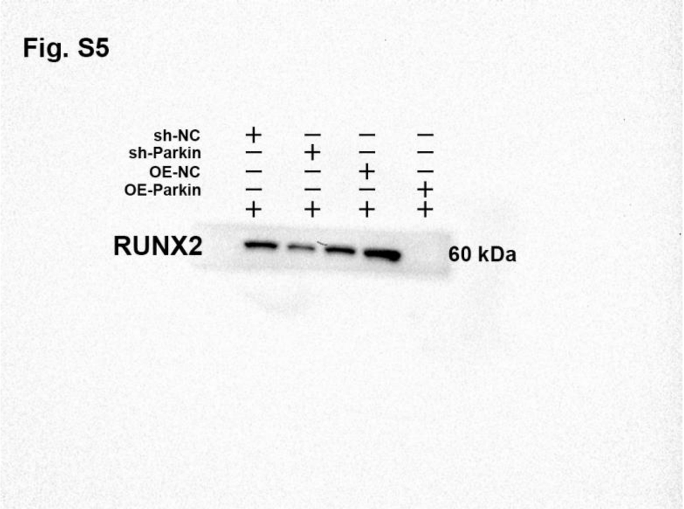

Fig. S5

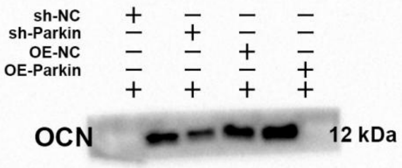

sh-NC
sh-Parkin
OE-NC
OE-Parkin
OCN
12 kDa

Fig. S5

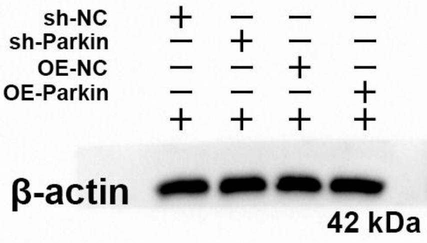

sh-NC
sh-Parkin
OE-NC
OE-Parkin
β-actin
42 kDa

Fig. S5C

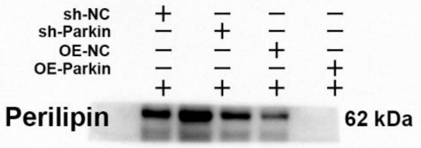

Fig. S5C

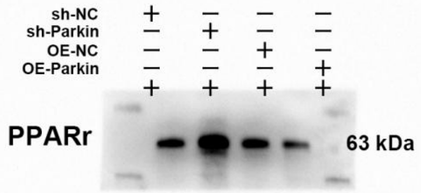

Fig. S5C

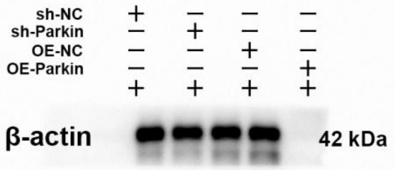

Fig. S6A

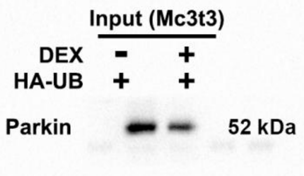

Fig. S6A

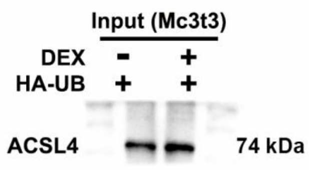

Fig. S6A

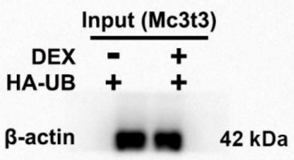

Fig. S6B

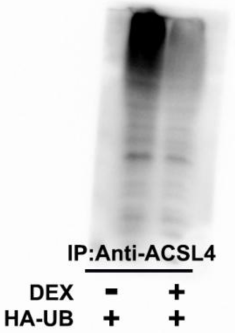

Fig. S6B

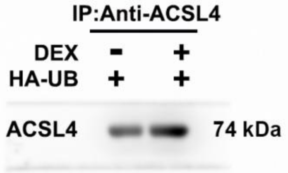

Fig. S6C

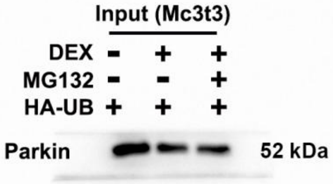

Fig. S6C

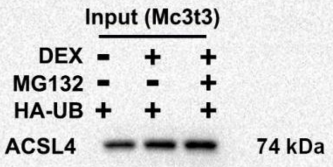

Fig. S6C

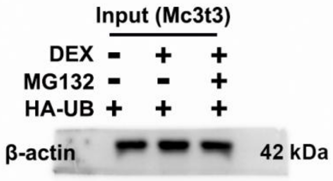

Fig. S6D

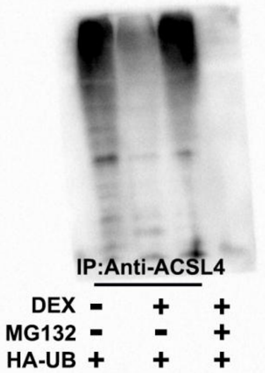

Fig. S6D

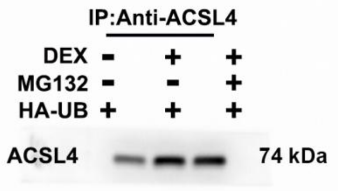

Fig. S7A

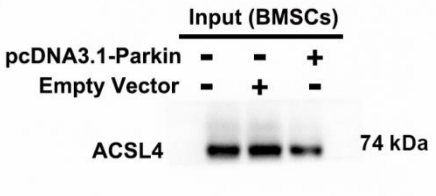

Fig. S7A

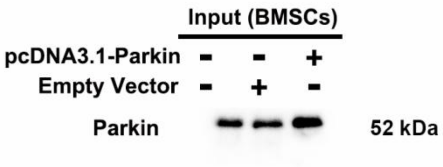

Fig. S7A

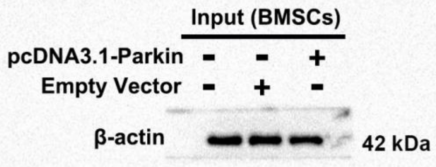

Fig. S7B

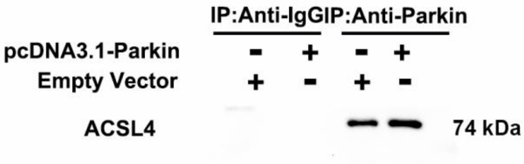

Fig. S7B

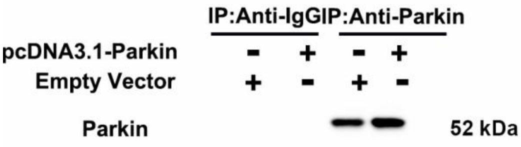

Fig. S7C

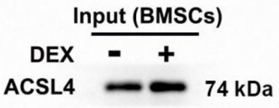

Fig. S7C

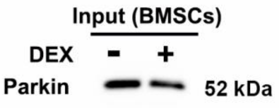

Fig. S7C

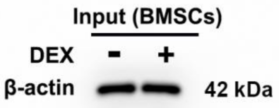

Fig. S7D

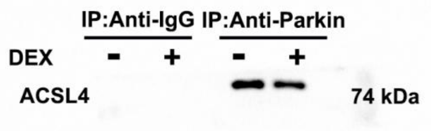

Fig. S7D

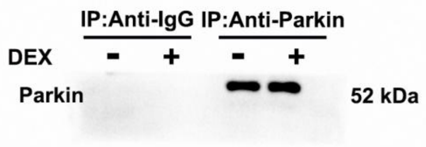

Fig. S7E

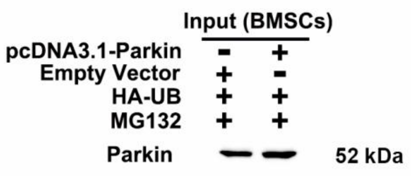

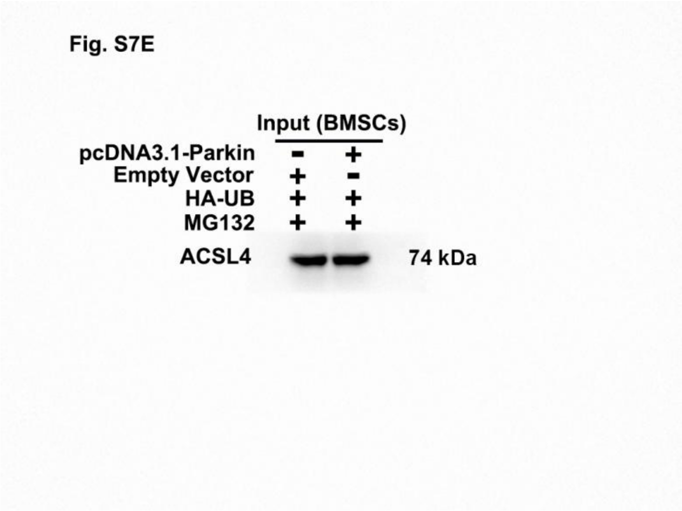
Fig. S7E
Input (BMSCs)
pcDNA3.1-Parkin - +
Empty Vector + -
HA-UB + +
MG132 + +
ACSL4
74 kDa

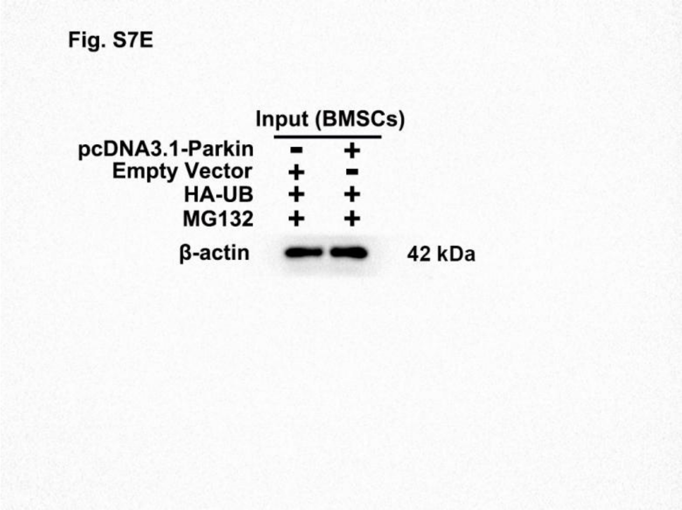
Fig. S7E
Input (BMSCs)
pcDNA3.1-Parkin - +
Empty Vector + -
HA-UB + +
MG132 + +
β-actin
42 kDa

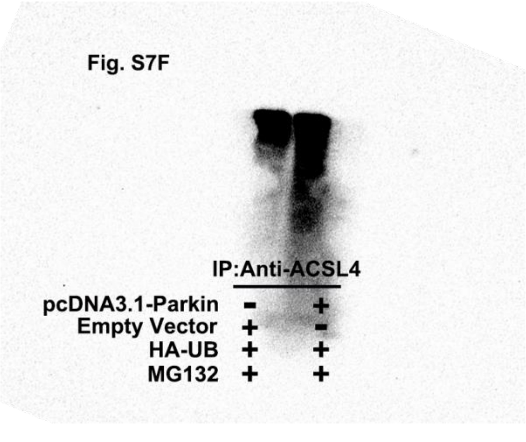
Fig. S7F
IP:Anti-ACSL4
pcDNA3.1-Parkin - +
Empty Vector + -
HA-UB + +
MG132 + +

Fig. S7F

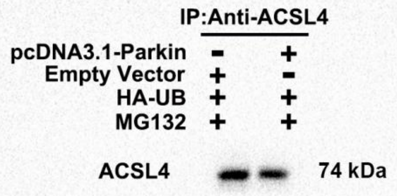

Fig. S7G

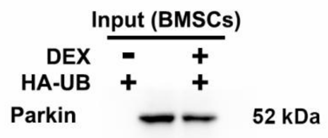

Fig. S7G

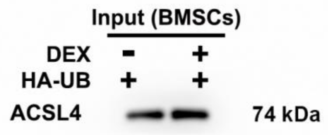

Fig. S7G

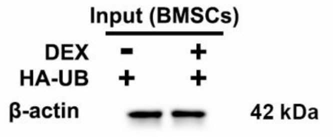

Fig. S7H

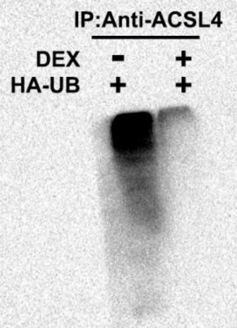

Fig. S7H

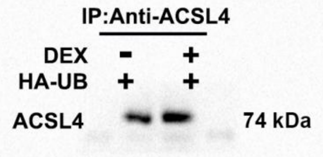

Fig. S7l

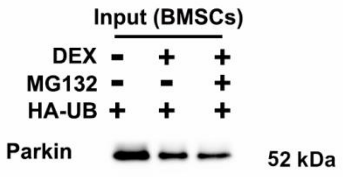

Fig. S7l

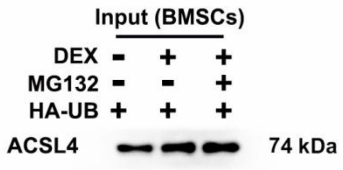

Fig. S7l

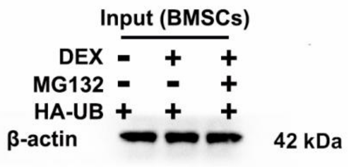

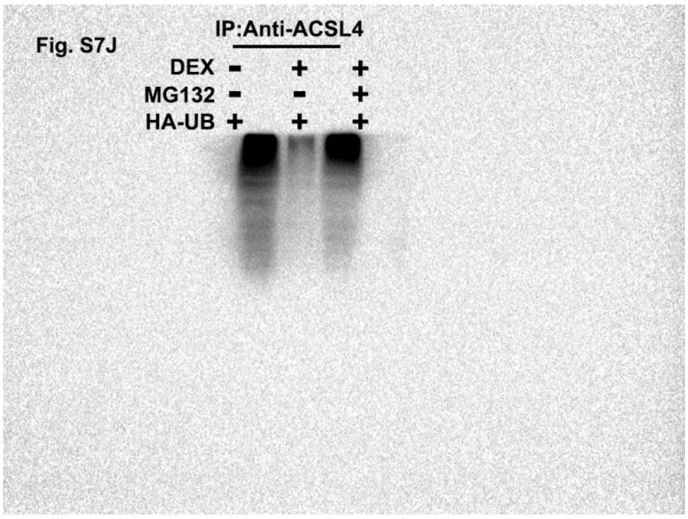
Fig. S7J
IP:Anti-ACSL4
DEX - + +
MG132 - - +
HA-UB + + +

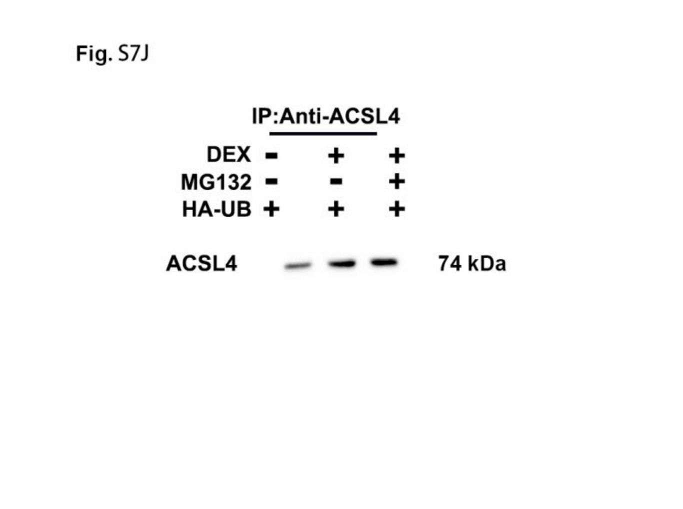
Fig. S7J
IP:Anti-ACSL4
DEX - + +
MG132 - - +
HA-UB + + +
ACSL4
74 kDa
